# Supplementary figures and images for: Glutamine synthetase mRNA releases sRNA from its 3′UTR to regulate carbon/nitrogen metabolic balance in Enterobacteriaceae
Source: eLife. 2022 Nov 28;11:e82411. doi: 10.7554/eLife.82411 (PMC9731577; doi:10.7554/eLife.82411)

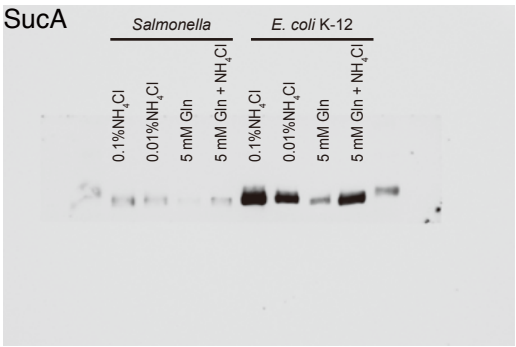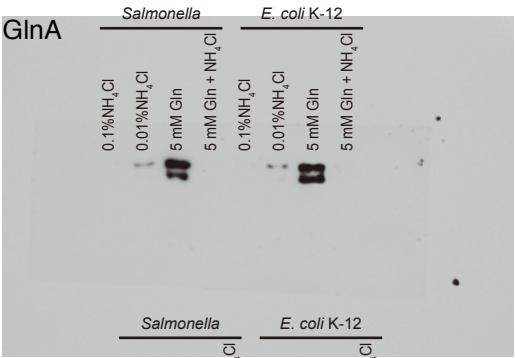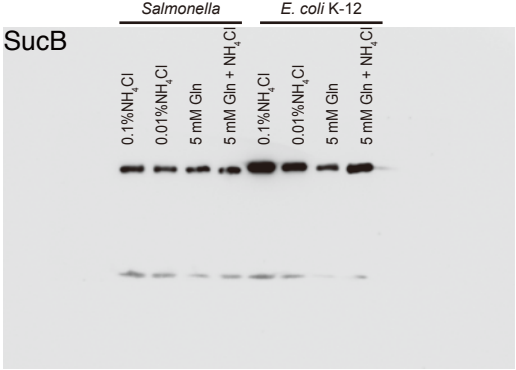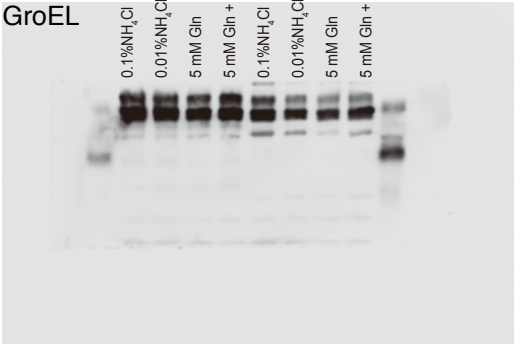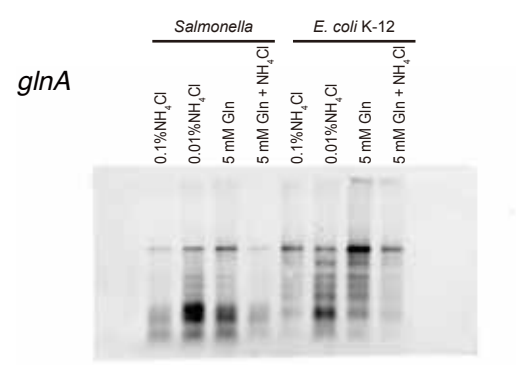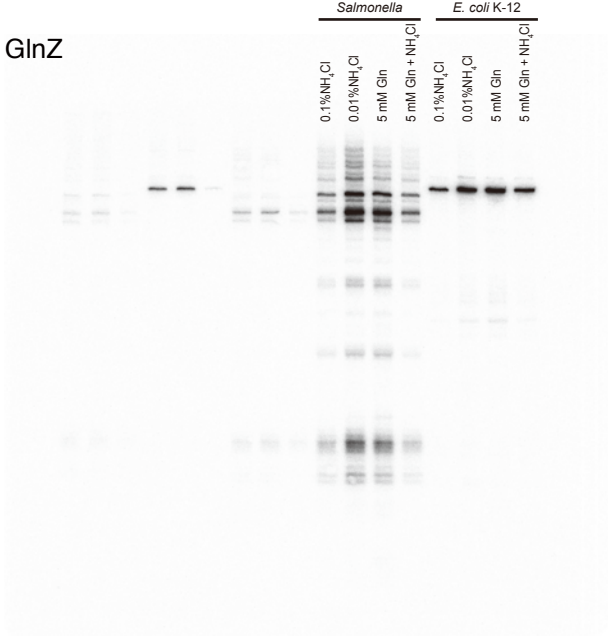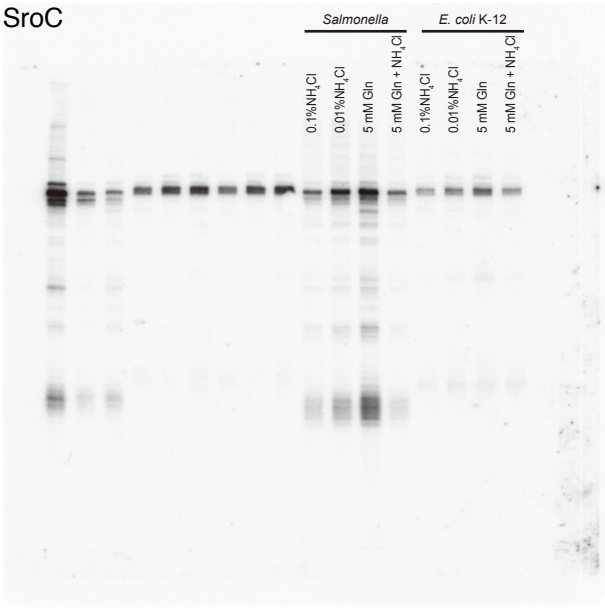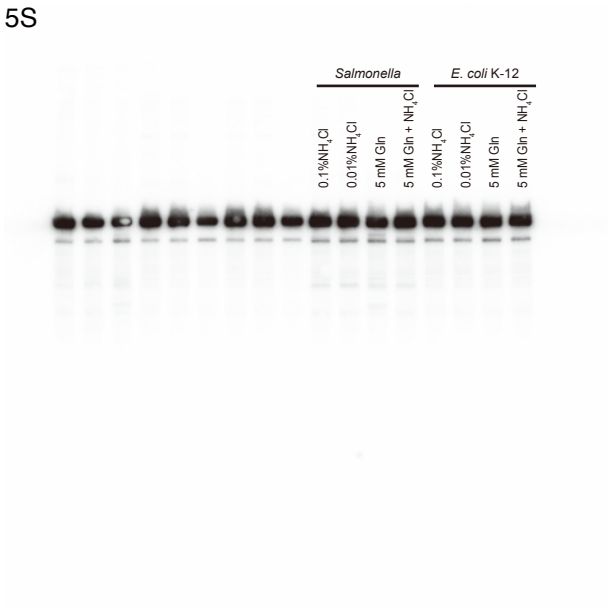

Supplement: Figure 1—source data 1. [file elife-82411-fig1-data1.pdf]

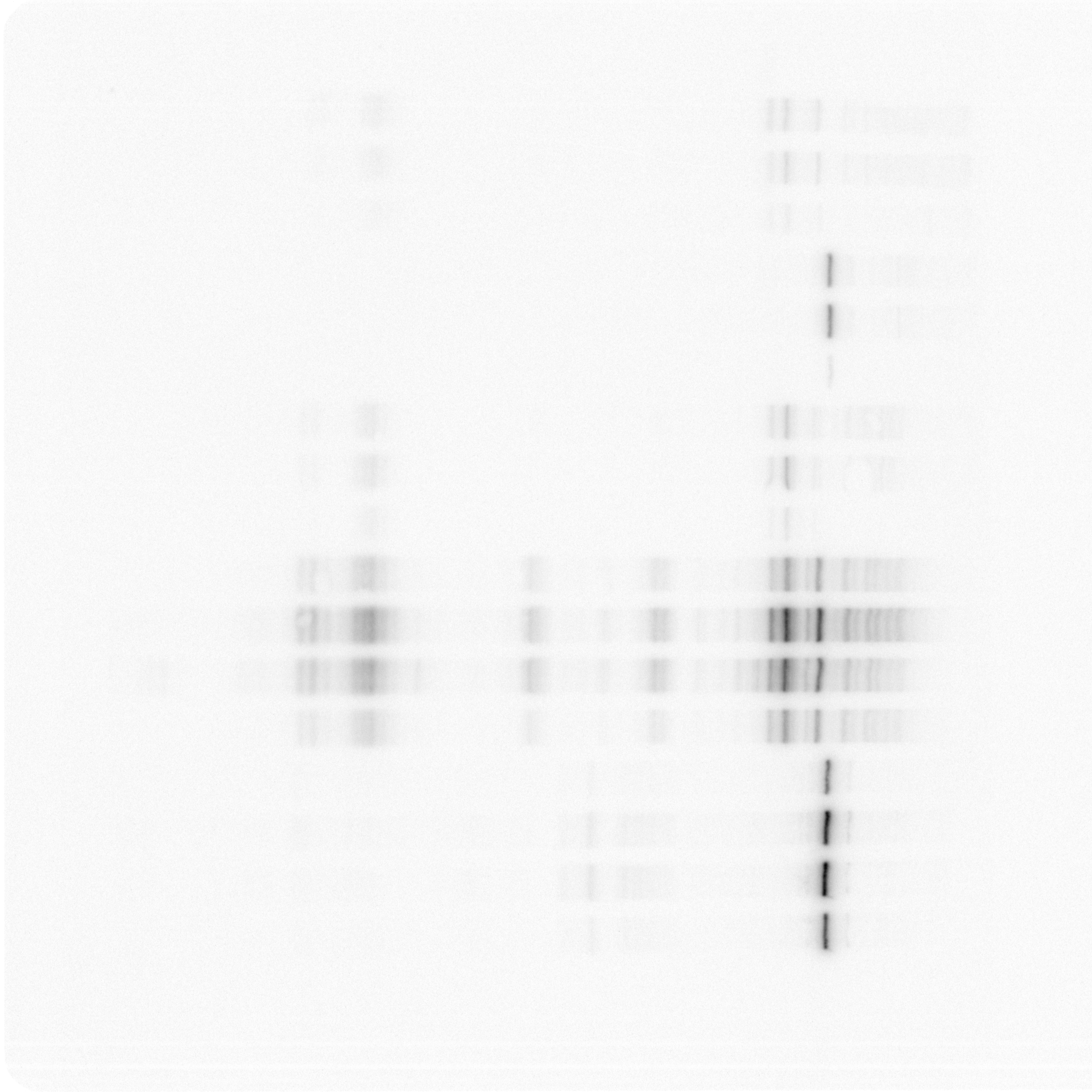

Supplement: Figure 1—source data 2. [file elife-82411-fig1-data2.zip › Fig1_SourceNB/Figure1_GlnZ.tif]

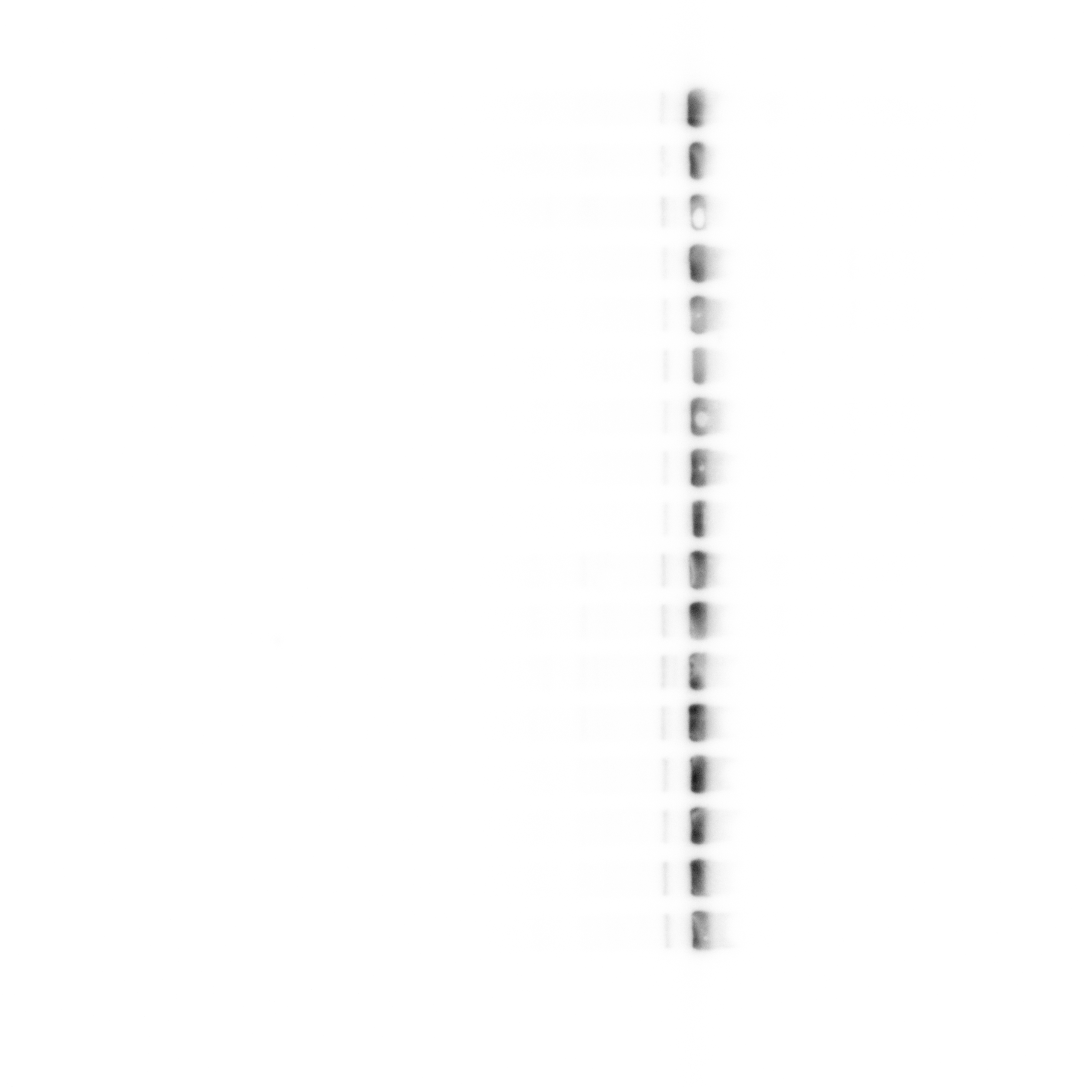

Supplement: Figure 1—source data 2. [file elife-82411-fig1-data2.zip › Fig1_SourceNB/Figure1_5S.tif]

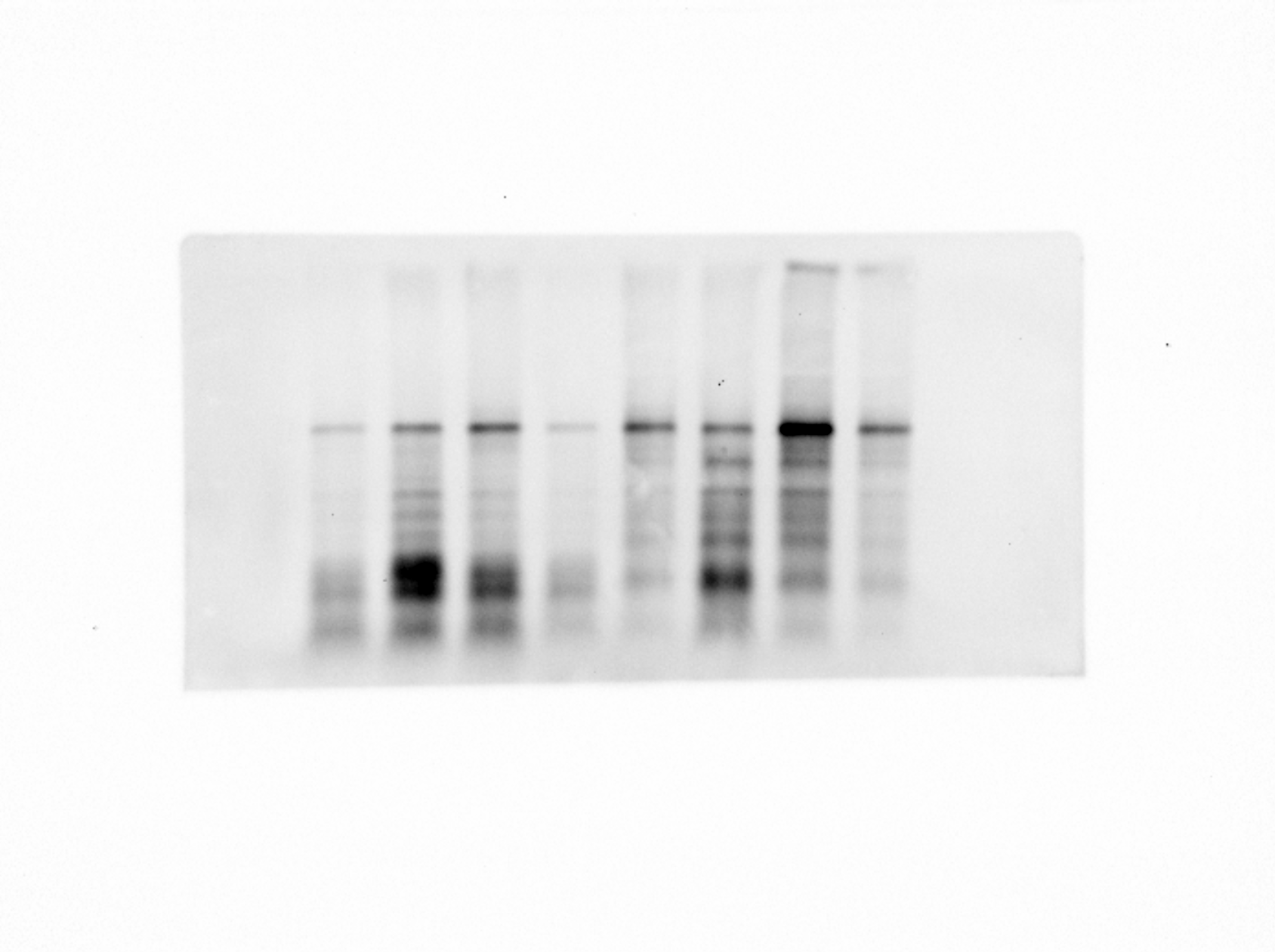

Supplement: Figure 1—source data 2. [file elife-82411-fig1-data2.zip › Fig1_SourceNB/Figure1_glnAmRNA.tif]

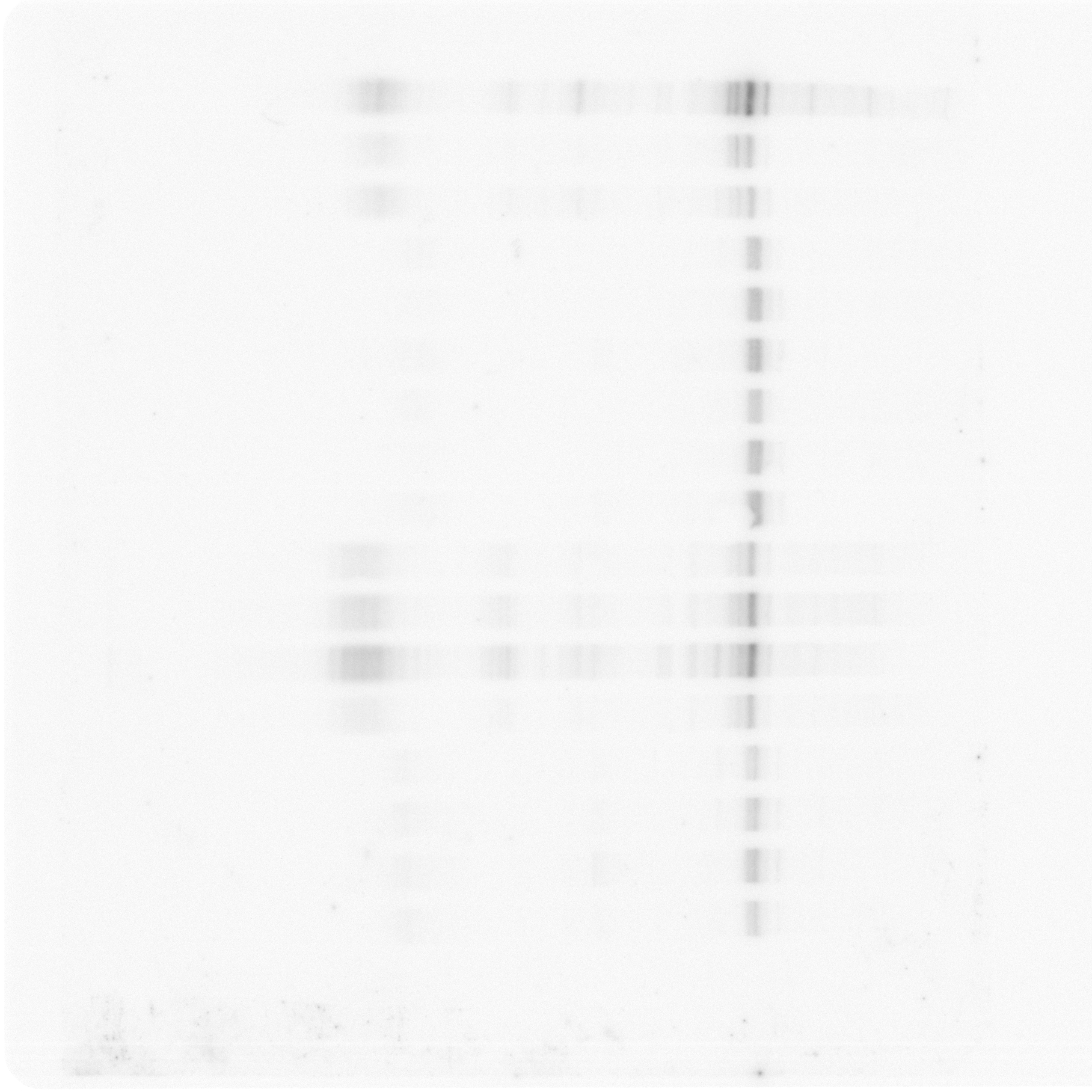

Supplement: Figure 1—source data 2. [file elife-82411-fig1-data2.zip › Fig1_SourceNB/Figure1_SroC.tif]

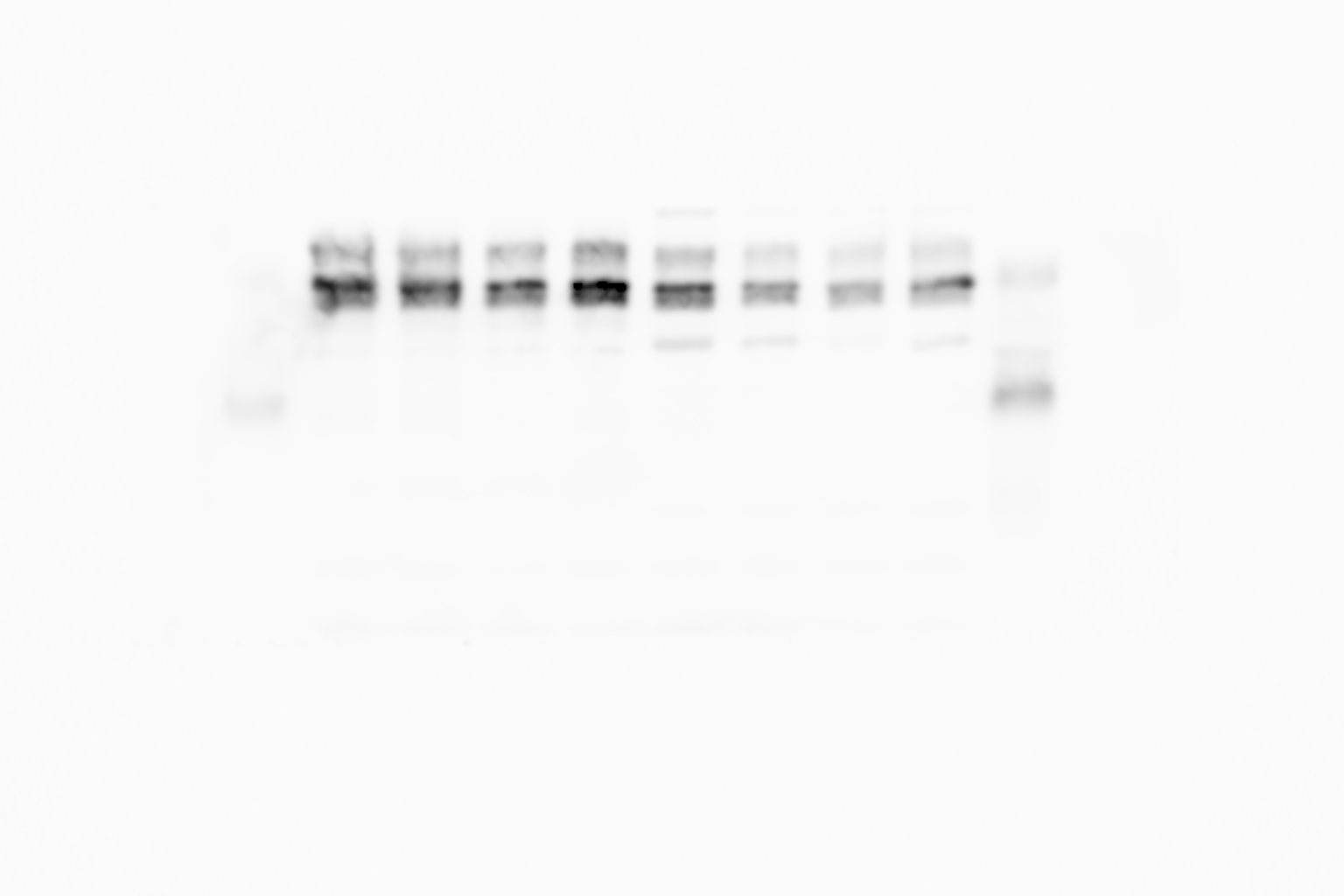

Supplement: Figure 1—source data 3. [file elife-82411-fig1-data3.zip › Fig1_SourceWB/Figure1_GroEL.tif]

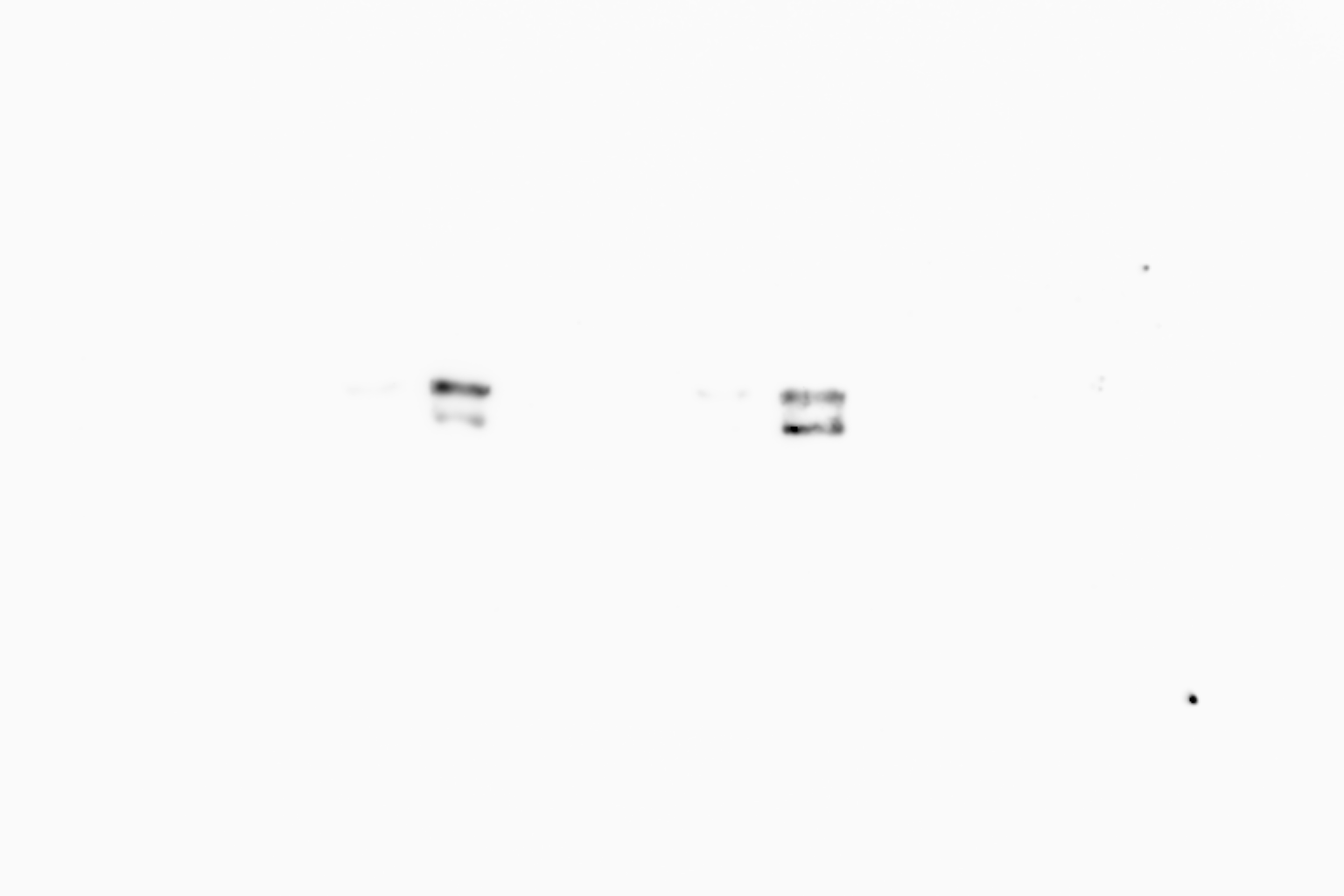

Supplement: Figure 1—source data 3. [file elife-82411-fig1-data3.zip › Fig1_SourceWB/Figure1_GlnA.tif]

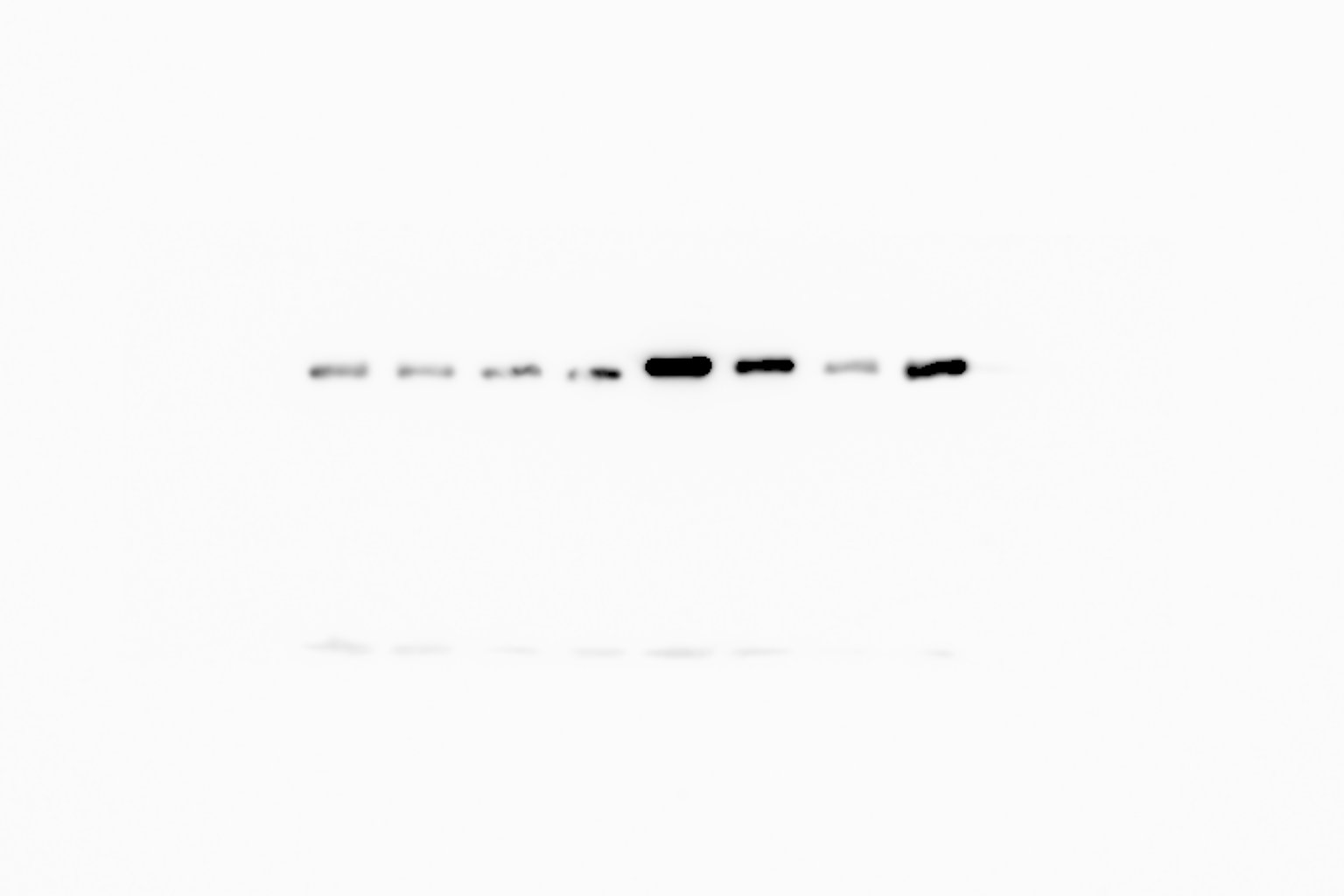

Supplement: Figure 1—source data 3. [file elife-82411-fig1-data3.zip › Fig1_SourceWB/Figure1_SucB.tif]

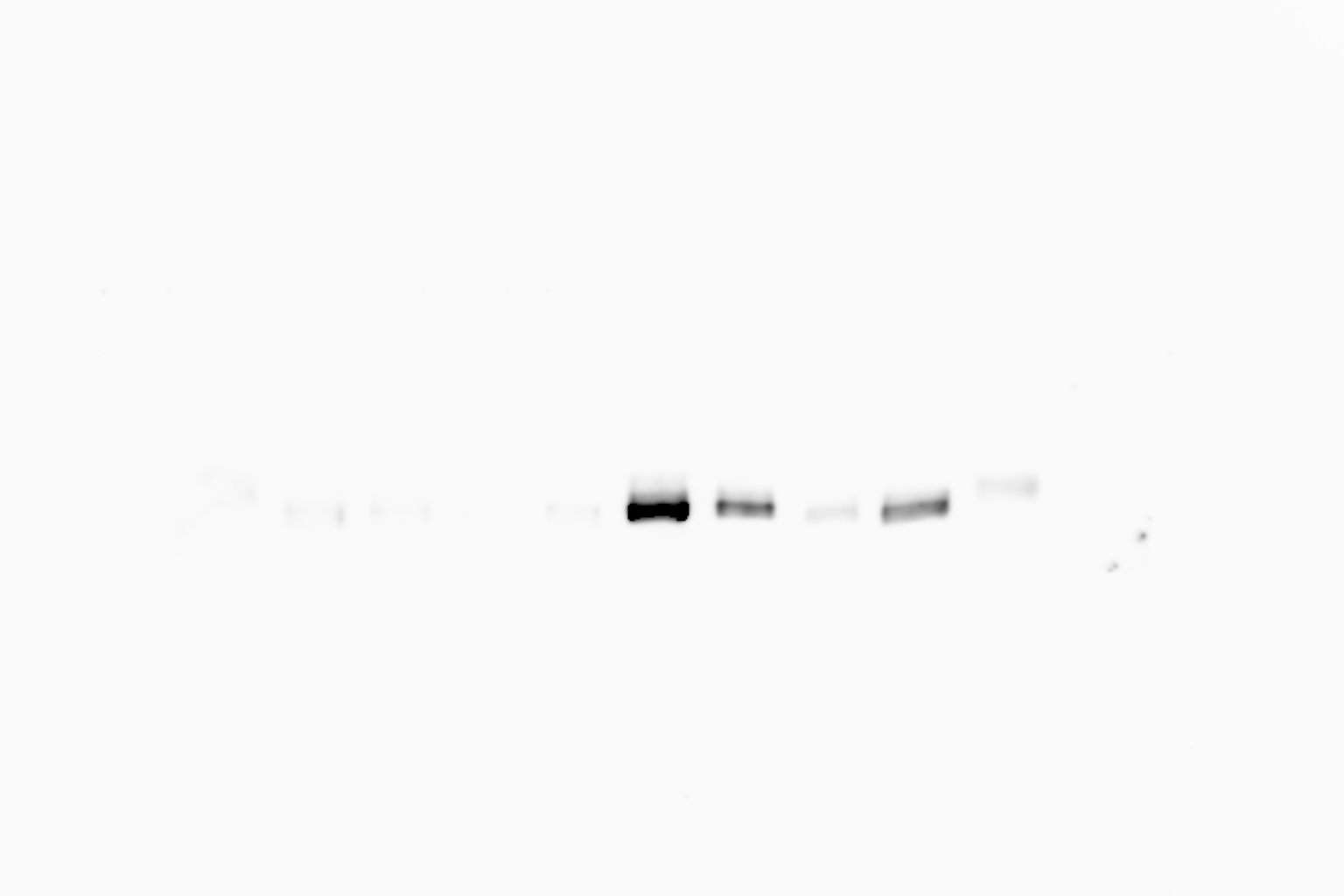

Supplement: Figure 1—source data 3. [file elife-82411-fig1-data3.zip › Fig1_SourceWB/Figure1_SucA.tif]

GlnZ

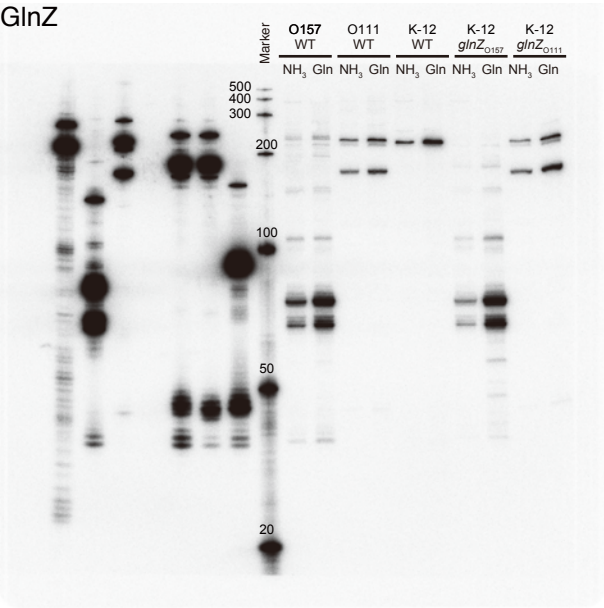

GlnA

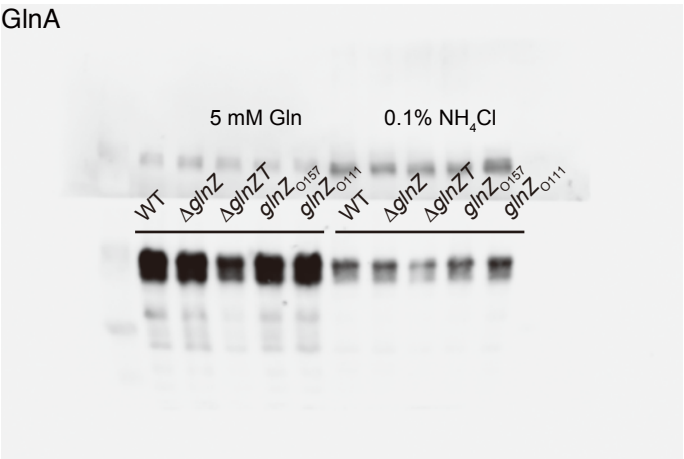

SroC

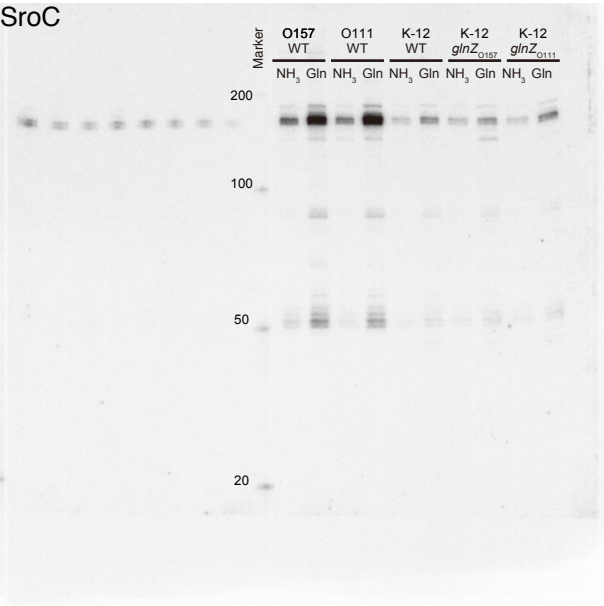

NtrC

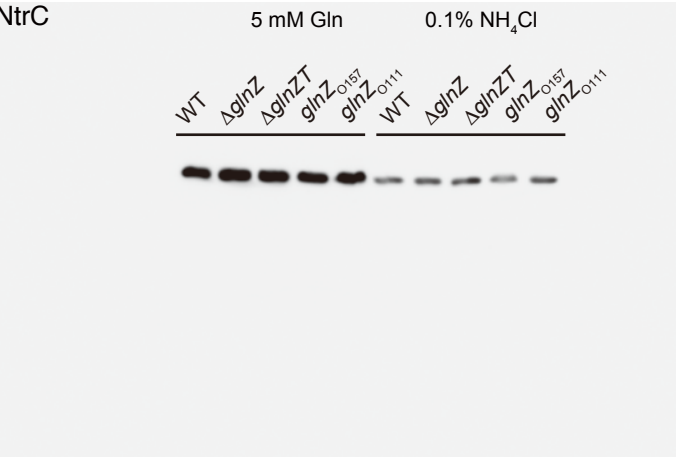

5S

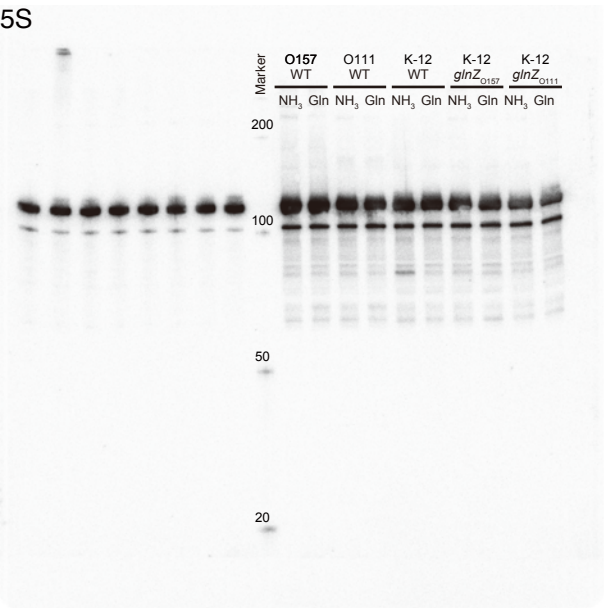

GroEL

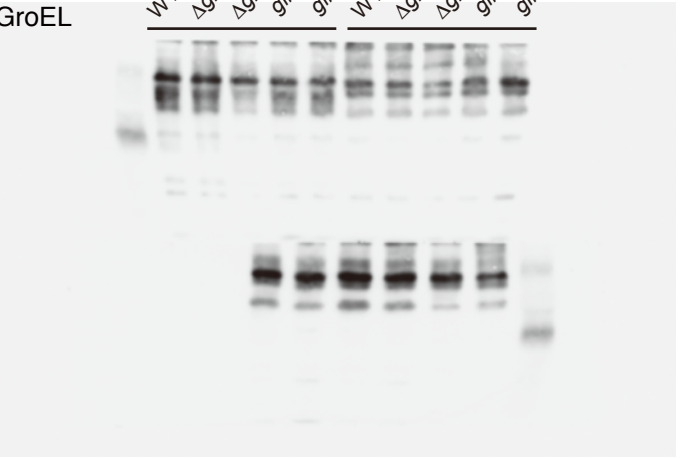

Supplement: Figure 2—source data 1. [file elife-82411-fig2-data1.pdf]

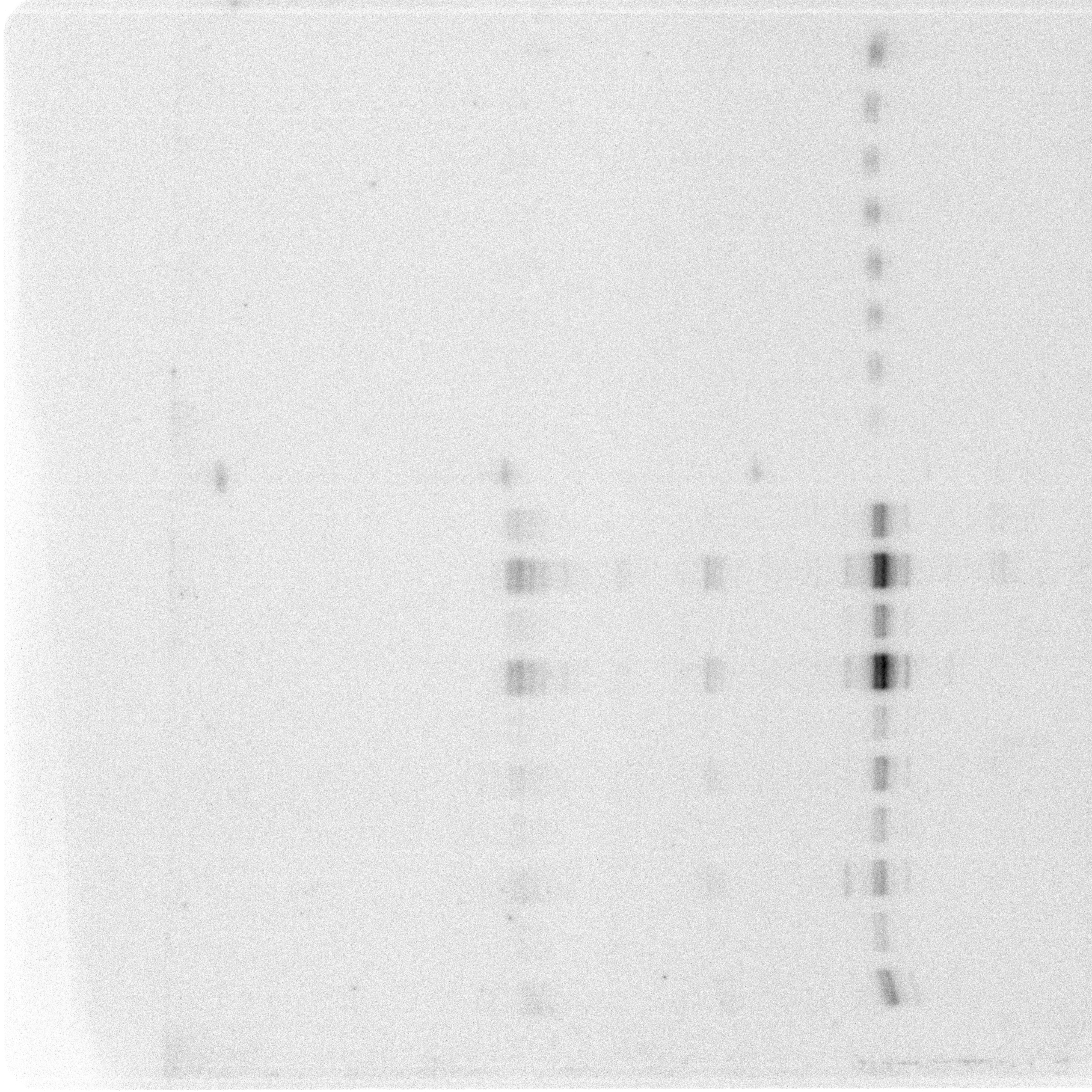

Supplement: Figure 2—source data 2. [file elife-82411-fig2-data2.zip › Fig2_SourceNB/Figure2_SroC.tif]

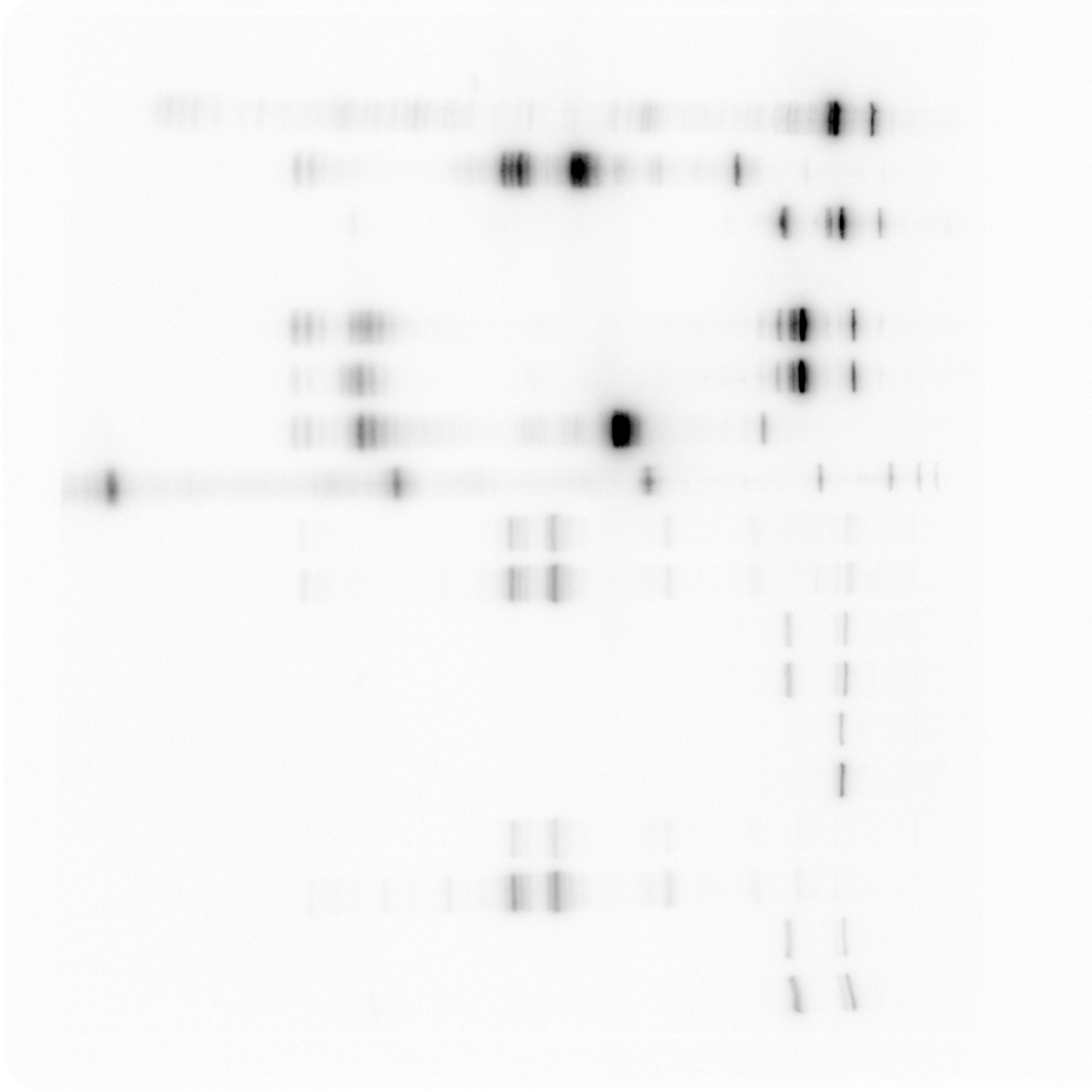

Supplement: Figure 2—source data 2. [file elife-82411-fig2-data2.zip › Fig2_SourceNB/Figure2_GlnZ.tif]

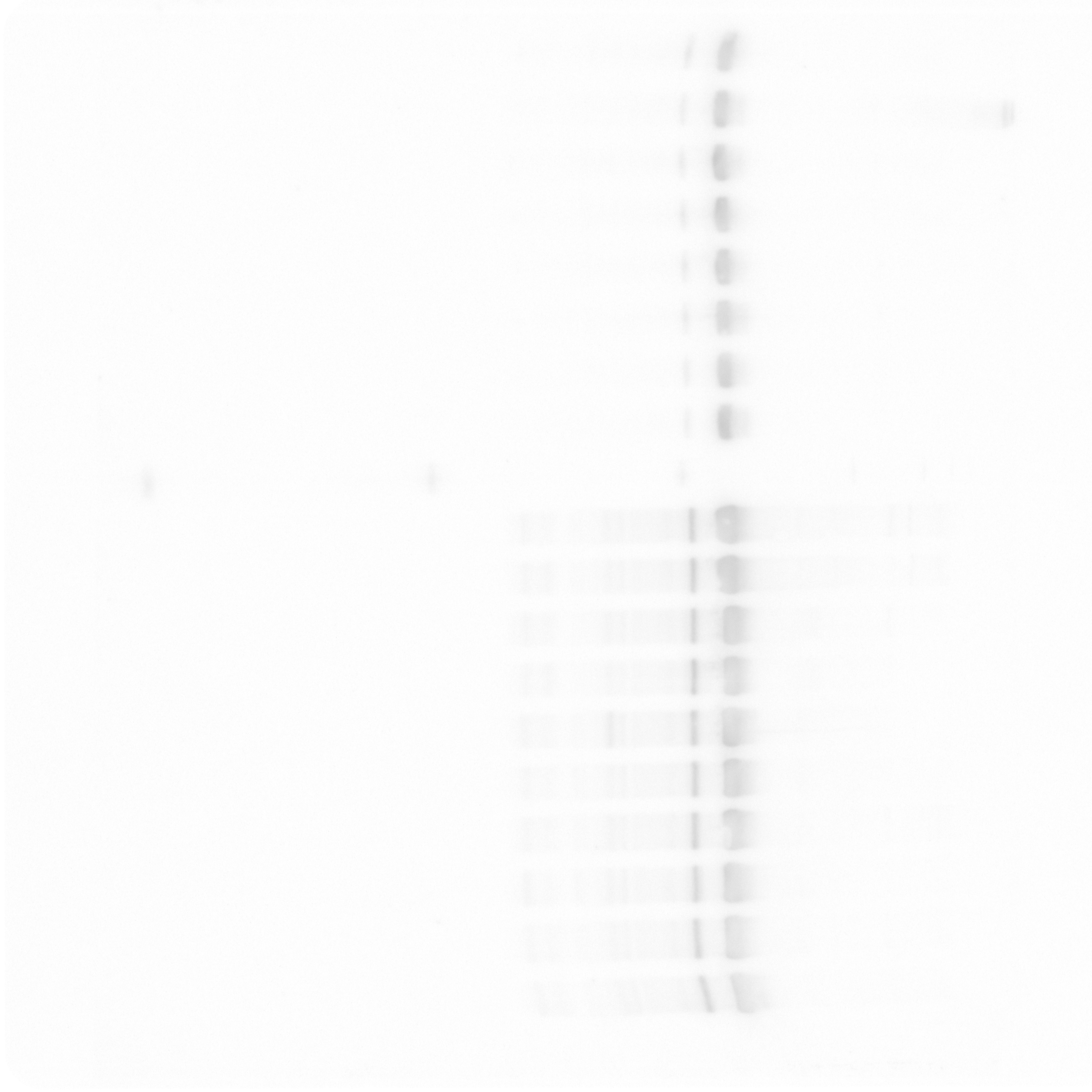

Supplement: Figure 2—source data 2. [file elife-82411-fig2-data2.zip › Fig2_SourceNB/Figure2_5S.tif]

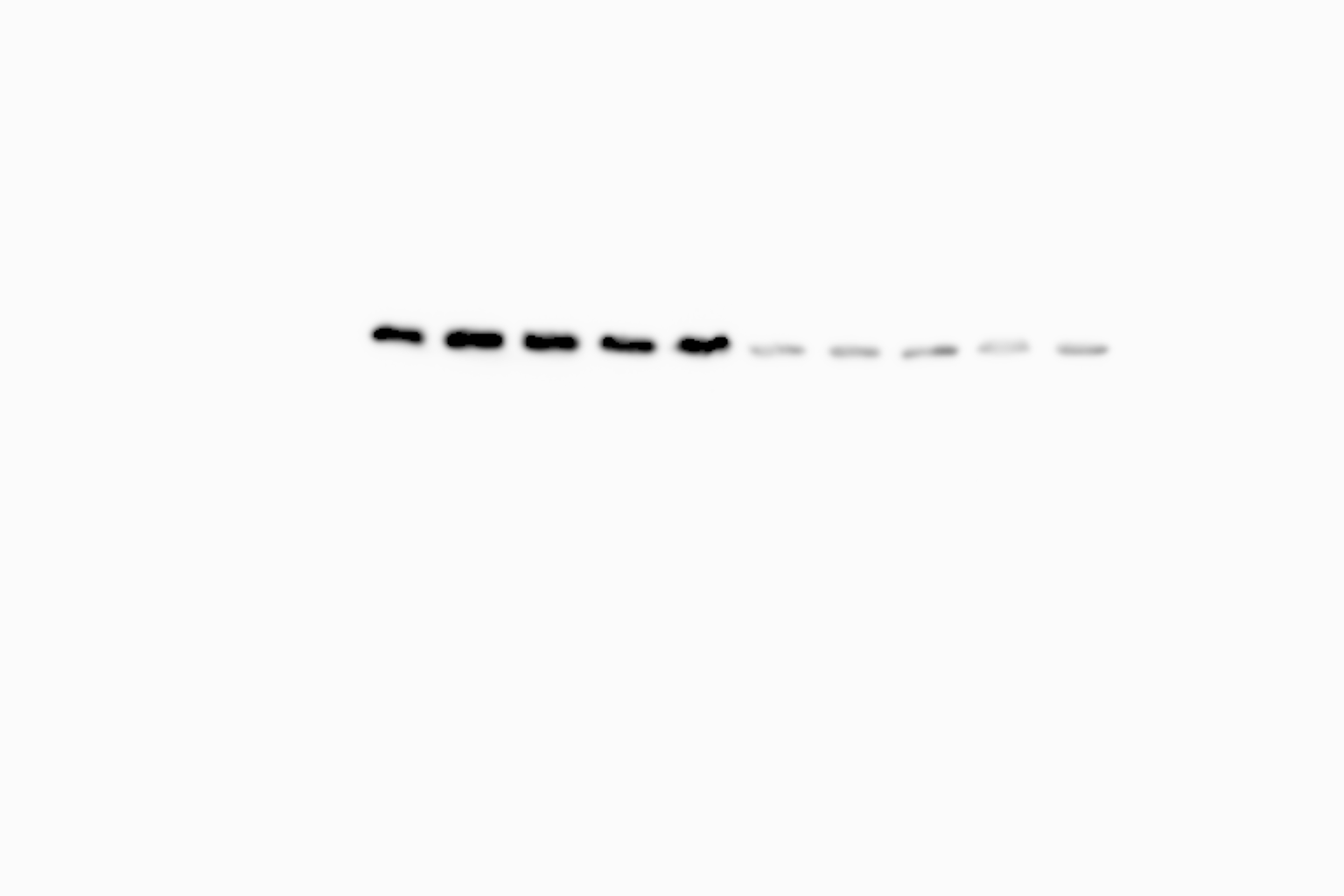

Supplement: Figure 2—source data 3. [file elife-82411-fig2-data3.zip › Fig2_SourceWB/Figure2_NtrC.tif]

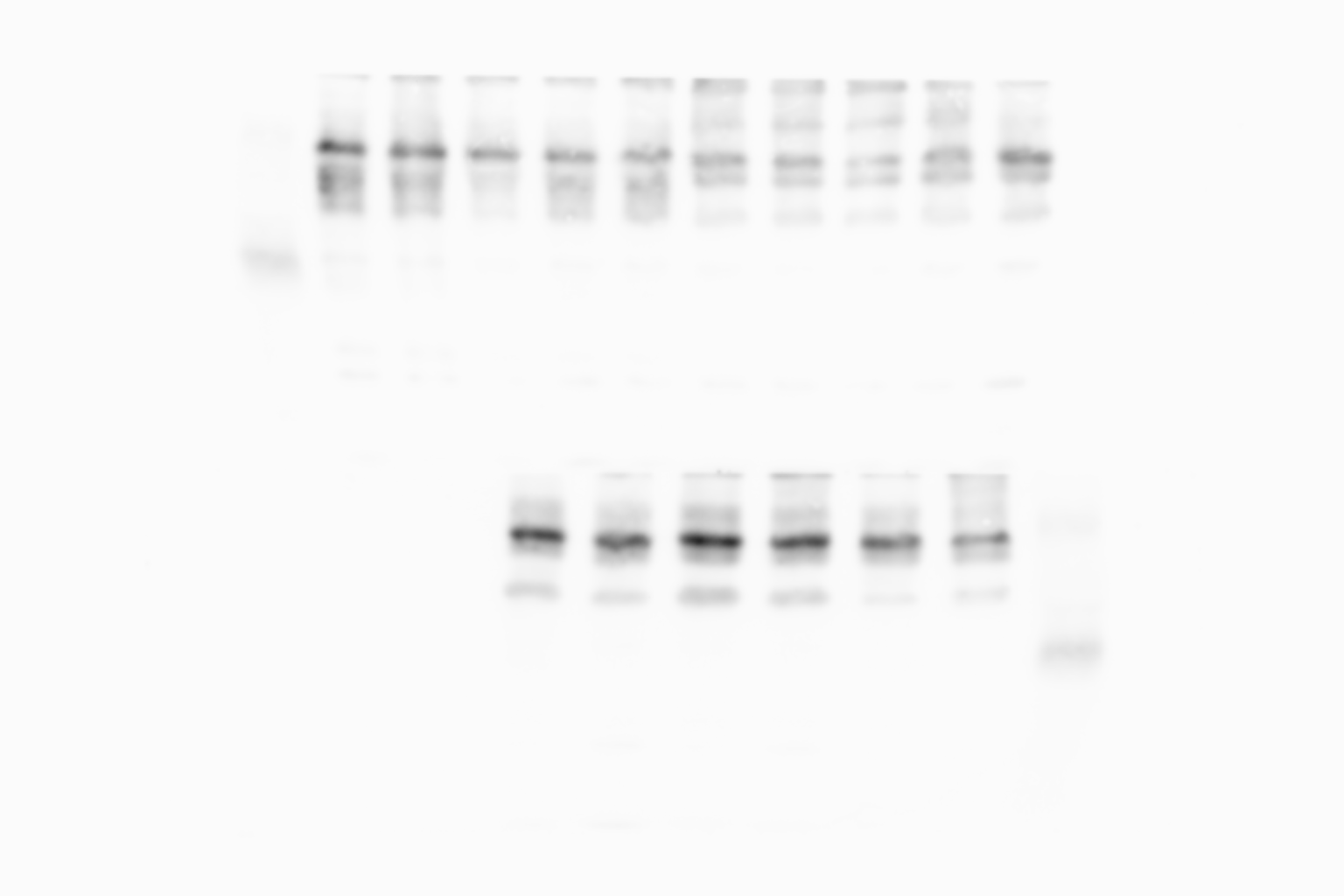

Supplement: Figure 2—source data 3. [file elife-82411-fig2-data3.zip › Fig2_SourceWB/Figure2_GroEL.tif]

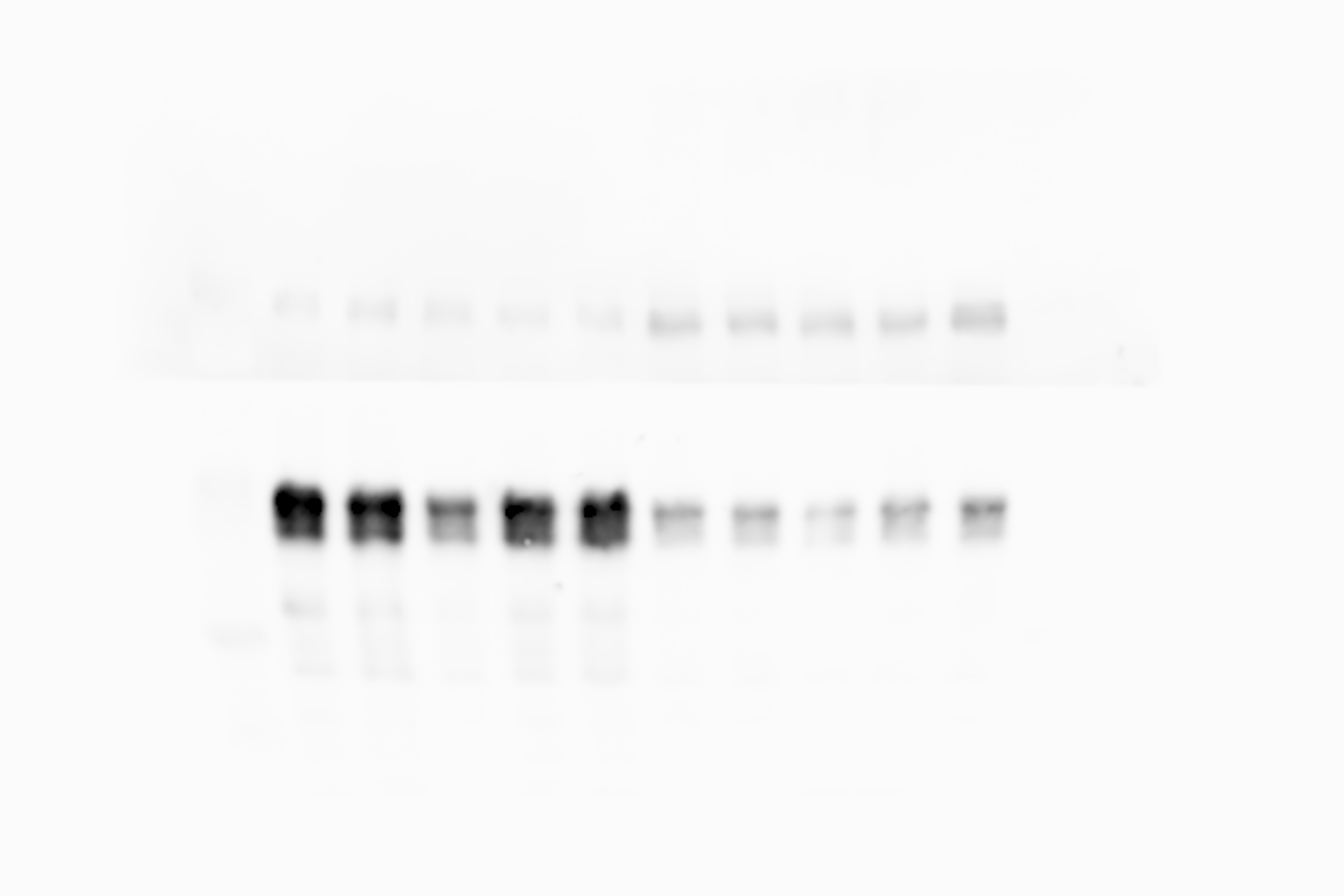

Supplement: Figure 2—source data 3. [file elife-82411-fig2-data3.zip › Fig2_SourceWB/Figure2_GlnA.tif]

A

SucA

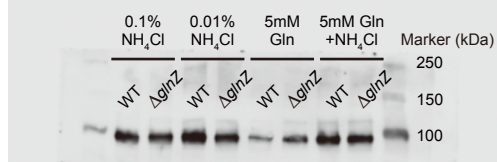

GlnA

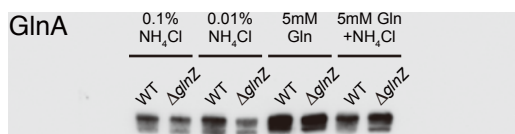

GroEL

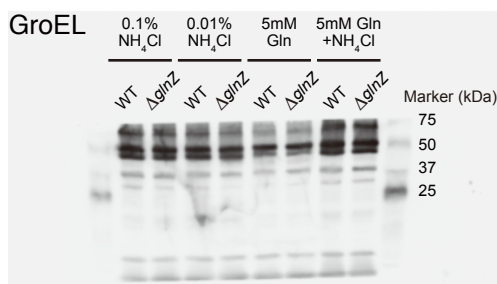

B

SucA

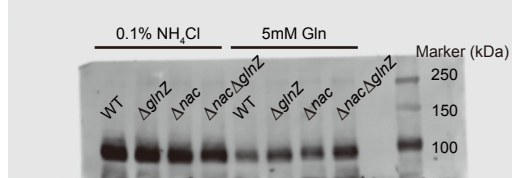

GlnA

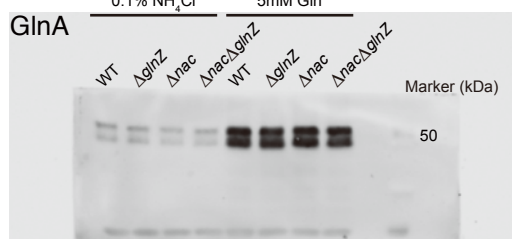

GroEL

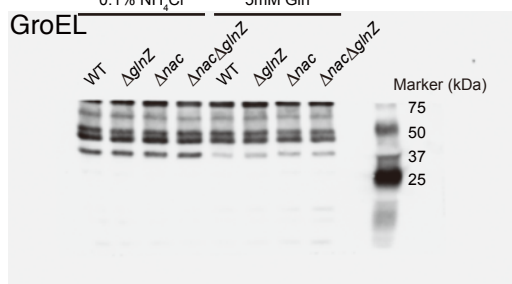

A

B

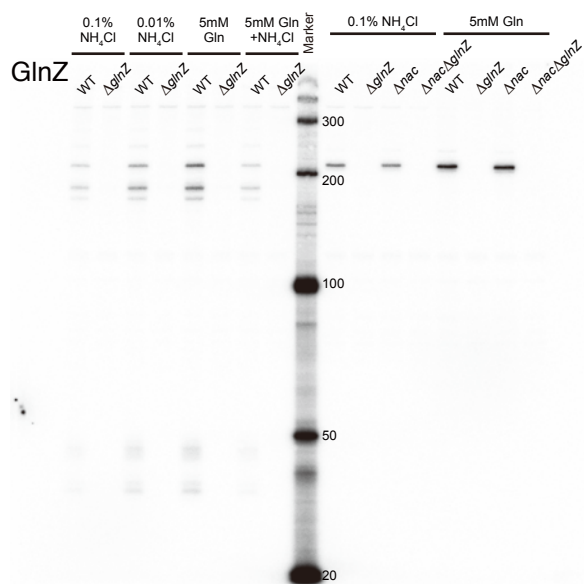

A

B

5S

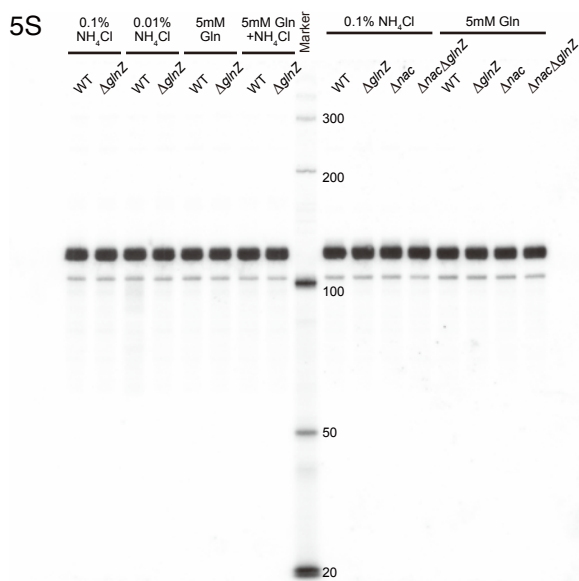

Supplement: Figure 4—source data 1. [file elife-82411-fig4-data1.pdf]

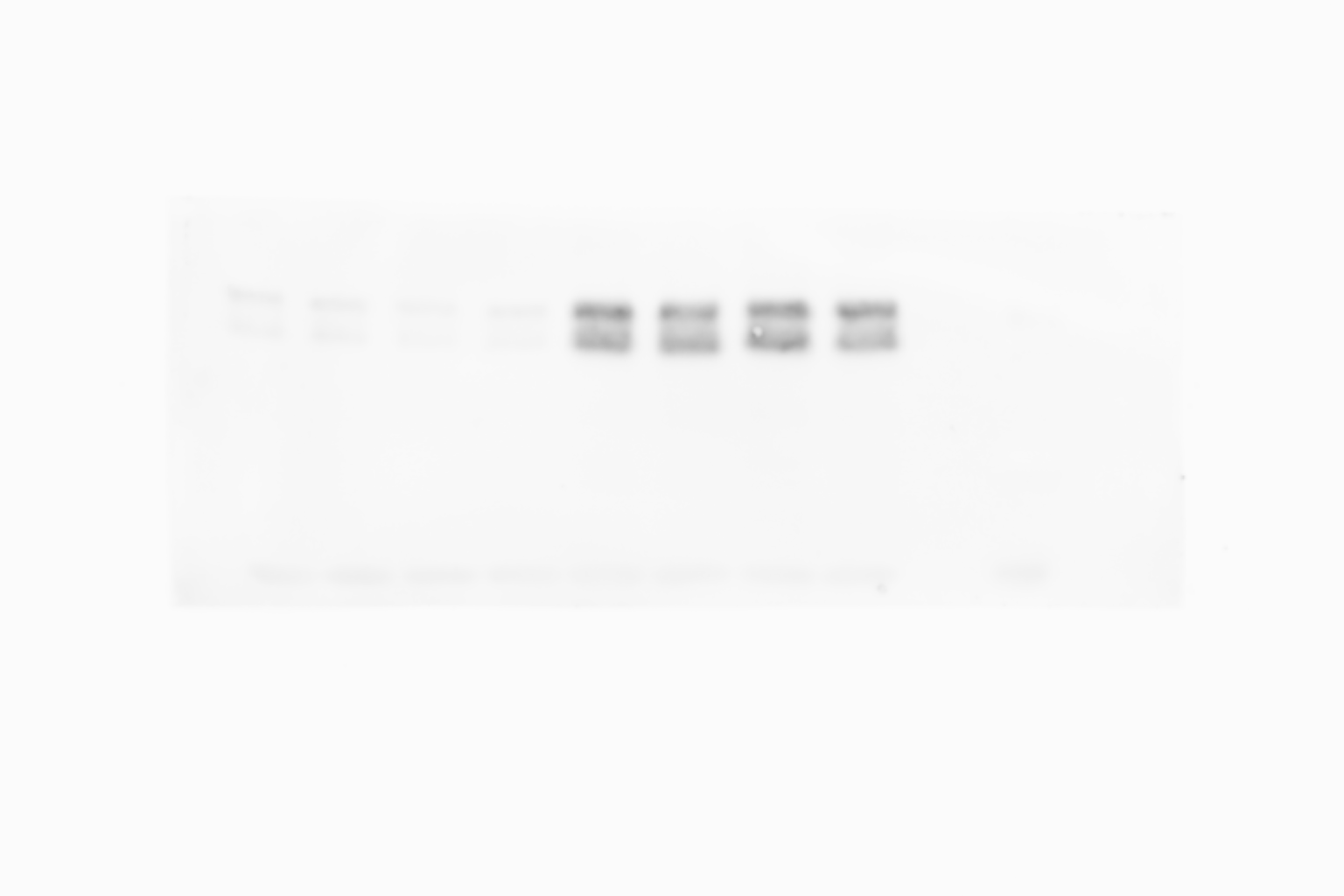

Supplement: Figure 4—source data 2. [file elife-82411-fig4-data2.zip › Fig4_Source/Figure4_GlnAeco.tif]

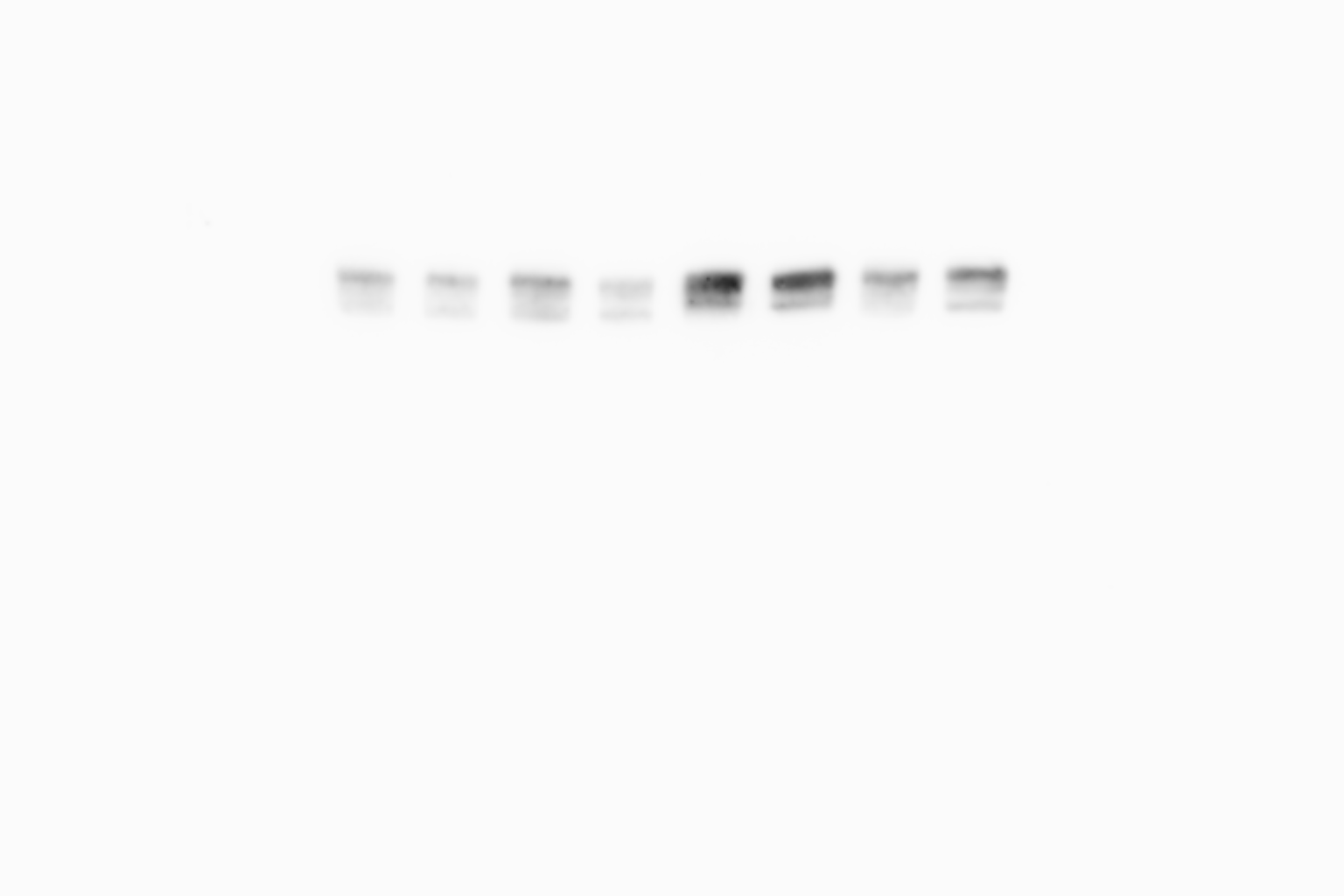

Supplement: Figure 4—source data 2. [file elife-82411-fig4-data2.zip › Fig4_Source/Figure4_GlnAsal.tif]

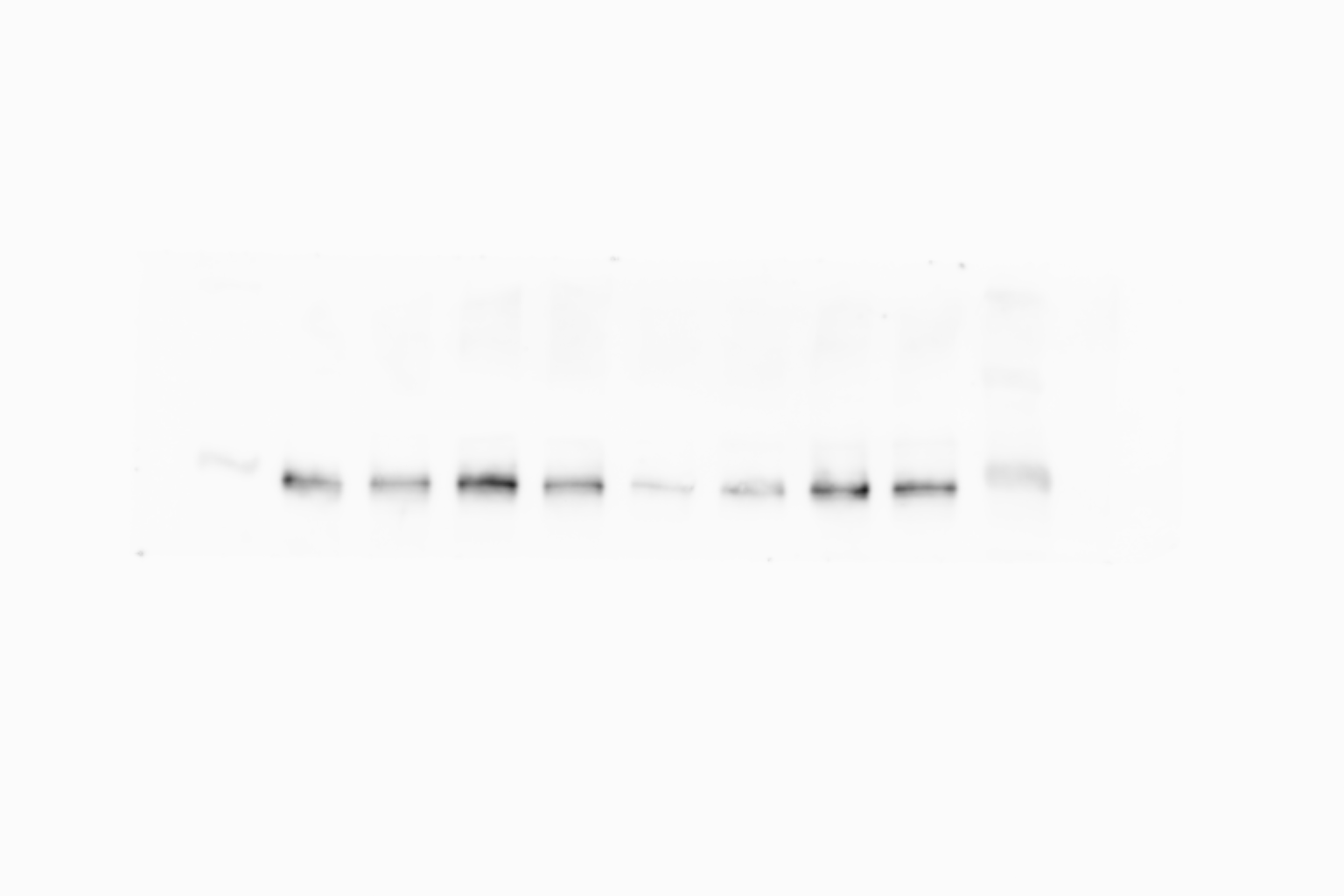

Supplement: Figure 4—source data 2. [file elife-82411-fig4-data2.zip › Fig4_Source/Figure4_SucAsal.tif]

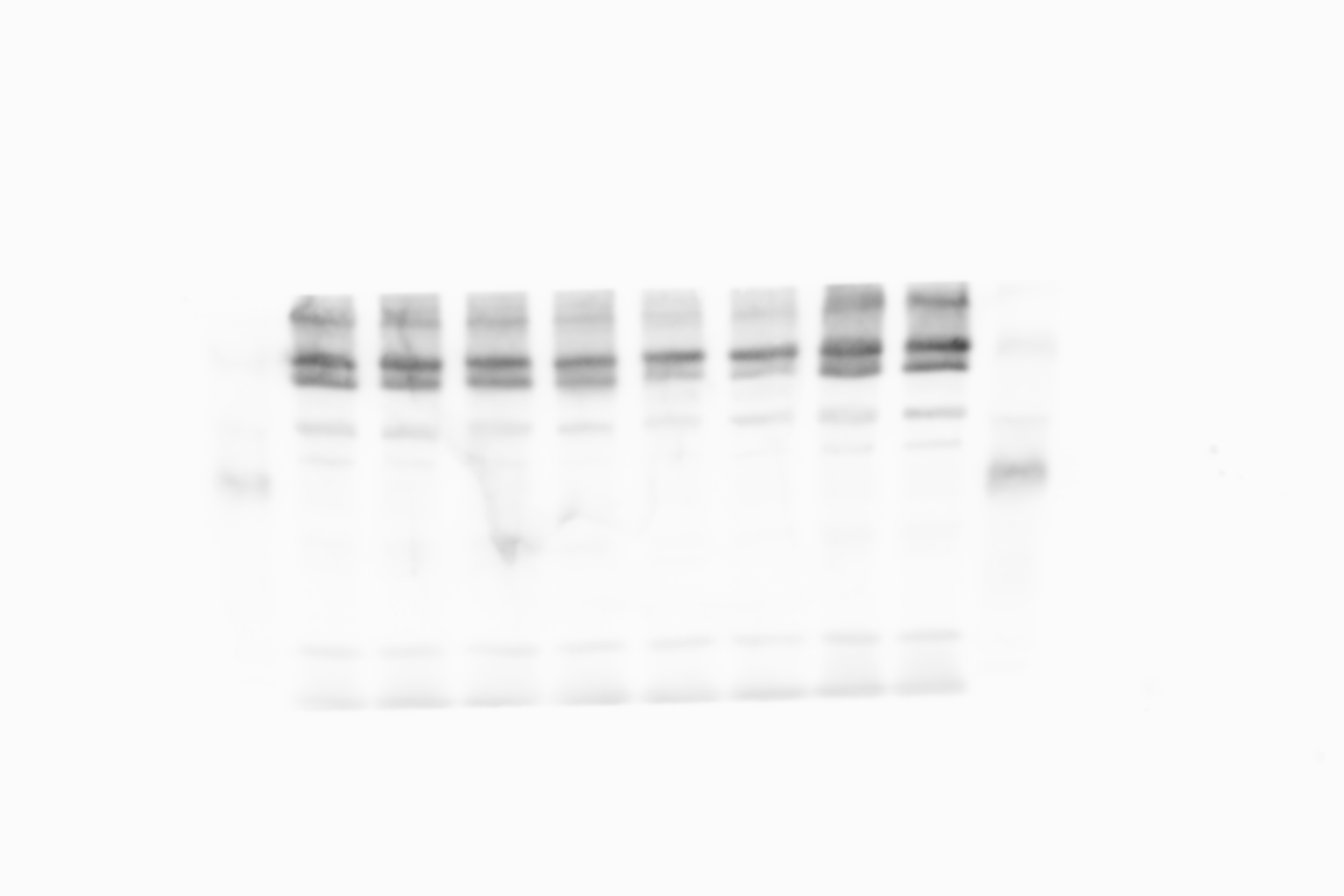

Supplement: Figure 4—source data 2. [file elife-82411-fig4-data2.zip › Fig4_Source/Figure4_GroELsal.tif]

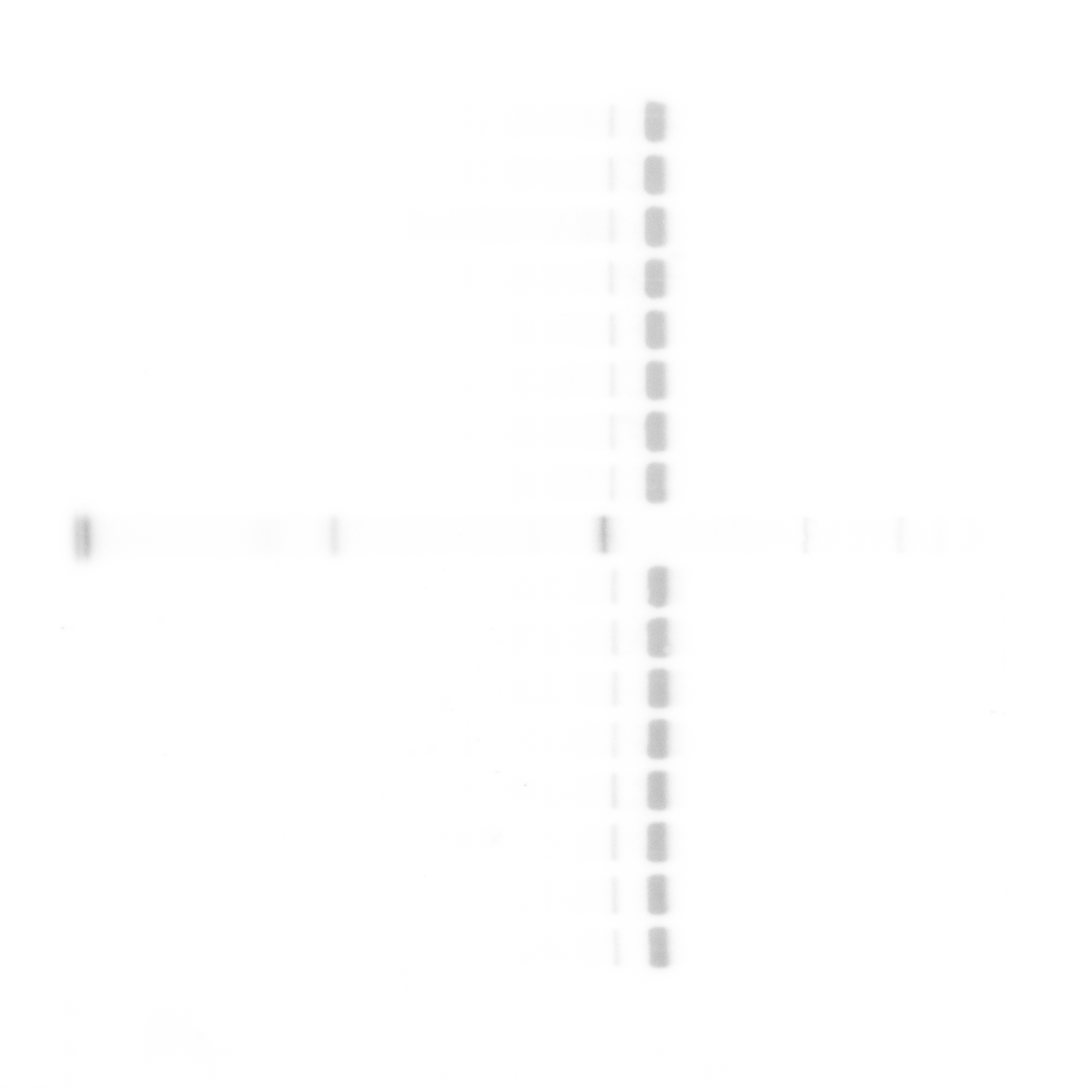

Supplement: Figure 4—source data 2. [file elife-82411-fig4-data2.zip › Fig4_Source/Figure4_5S.tif]

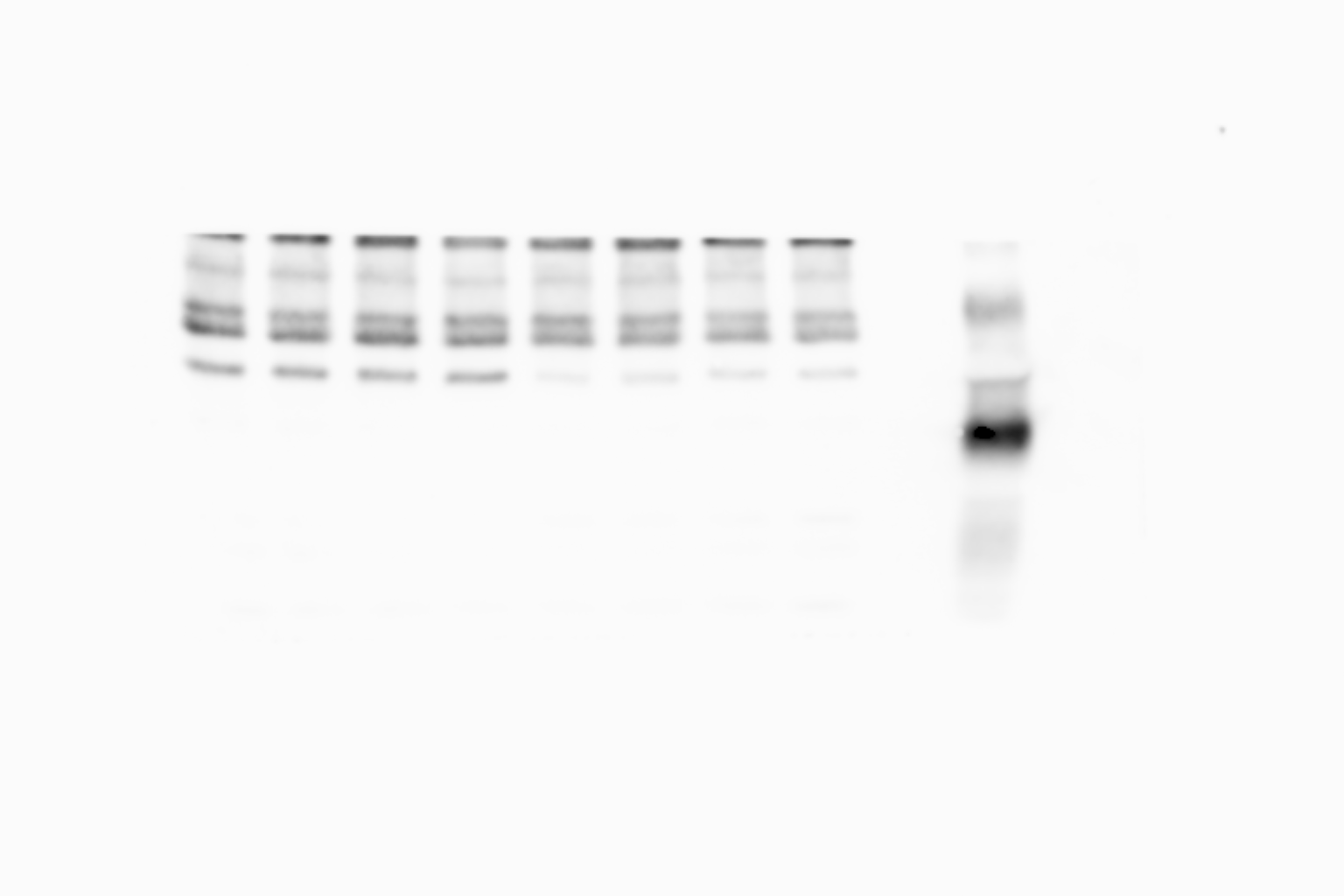

Supplement: Figure 4—source data 2. [file elife-82411-fig4-data2.zip › Fig4_Source/Figure4_GroELeco.tif]

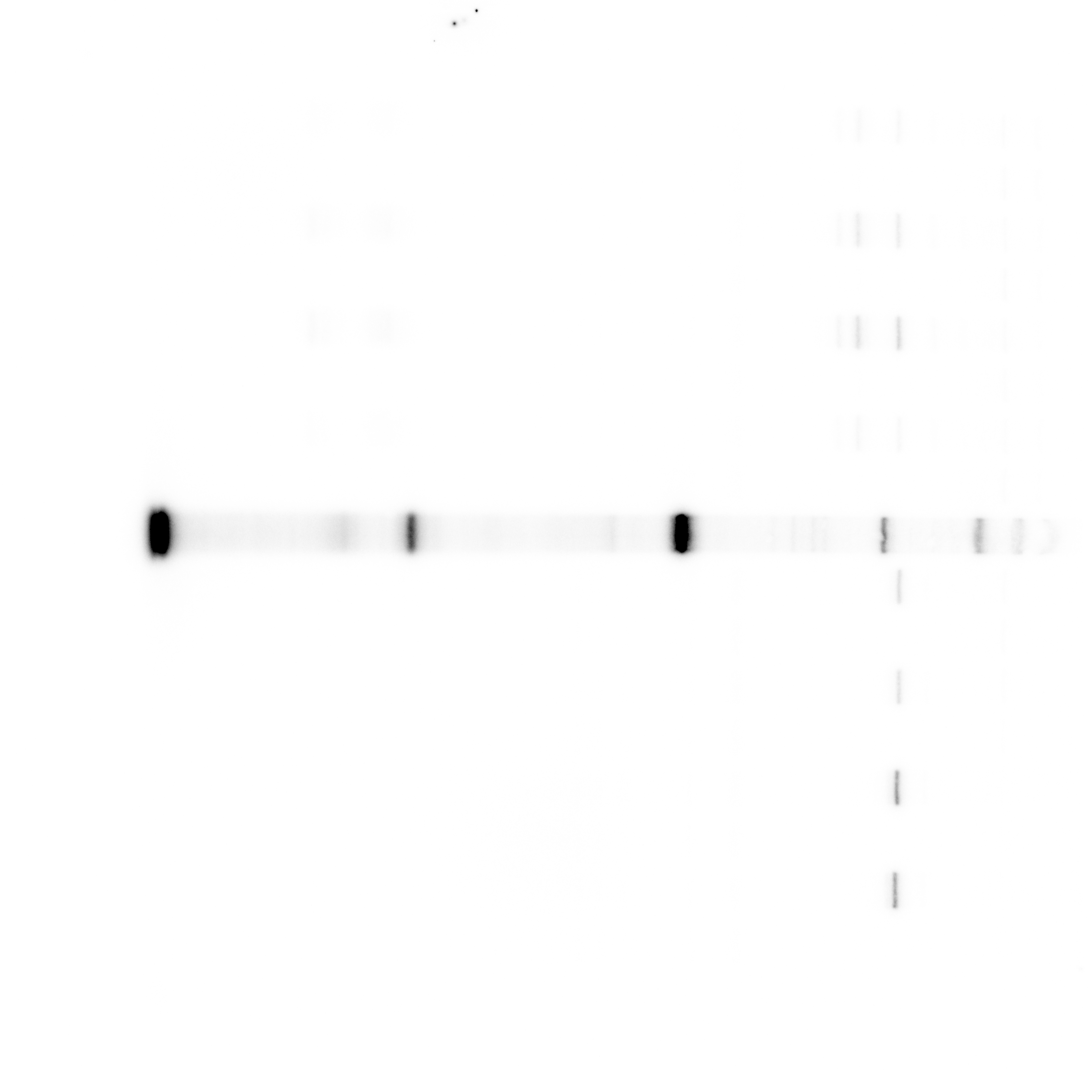

Supplement: Figure 4—source data 2. [file elife-82411-fig4-data2.zip › Fig4_Source/Figure4_GlnZ.tif]

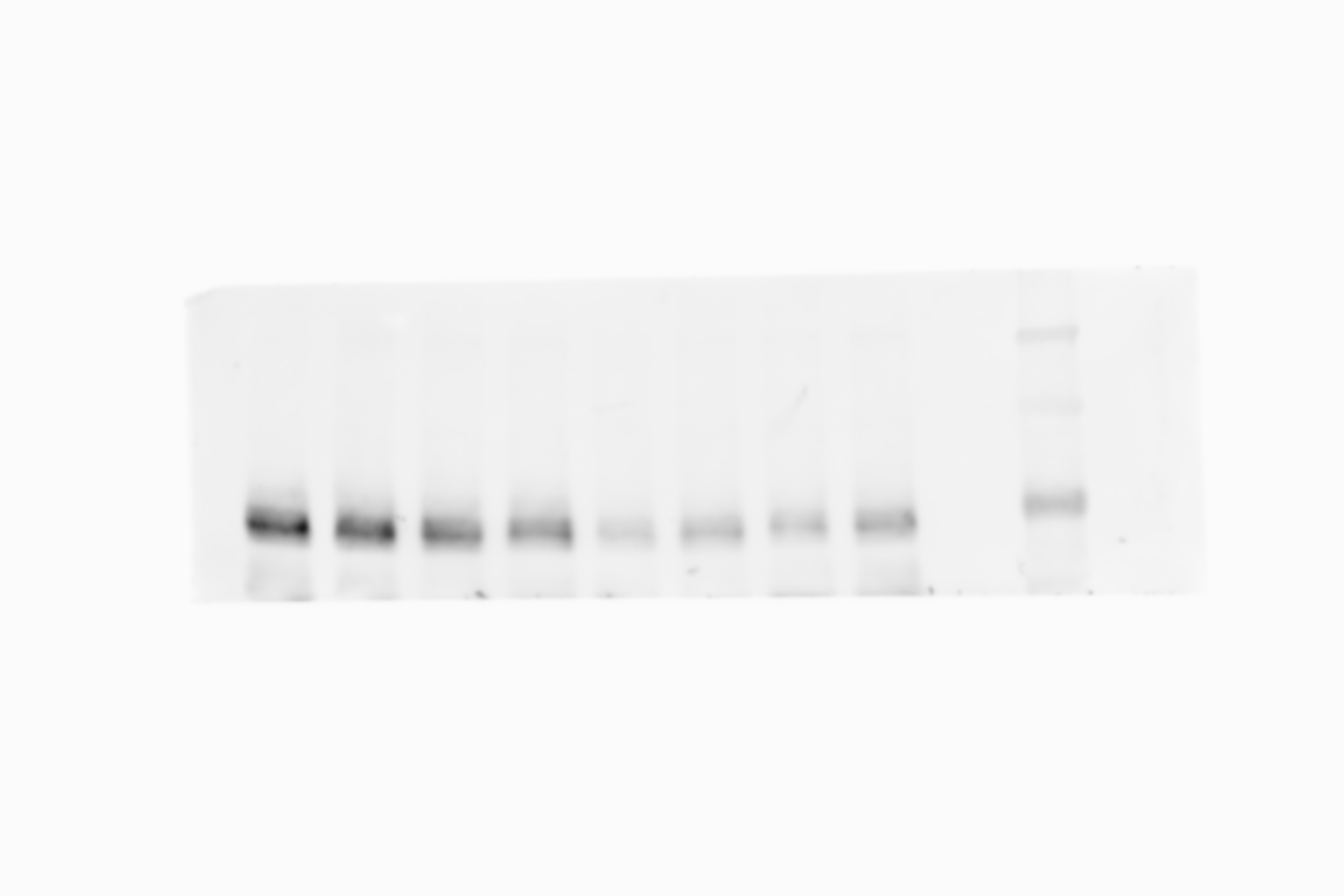

Supplement: Figure 4—source data 2. [file elife-82411-fig4-data2.zip › Fig4_Source/Figure4_SucAeco.tif]

Figure 5A

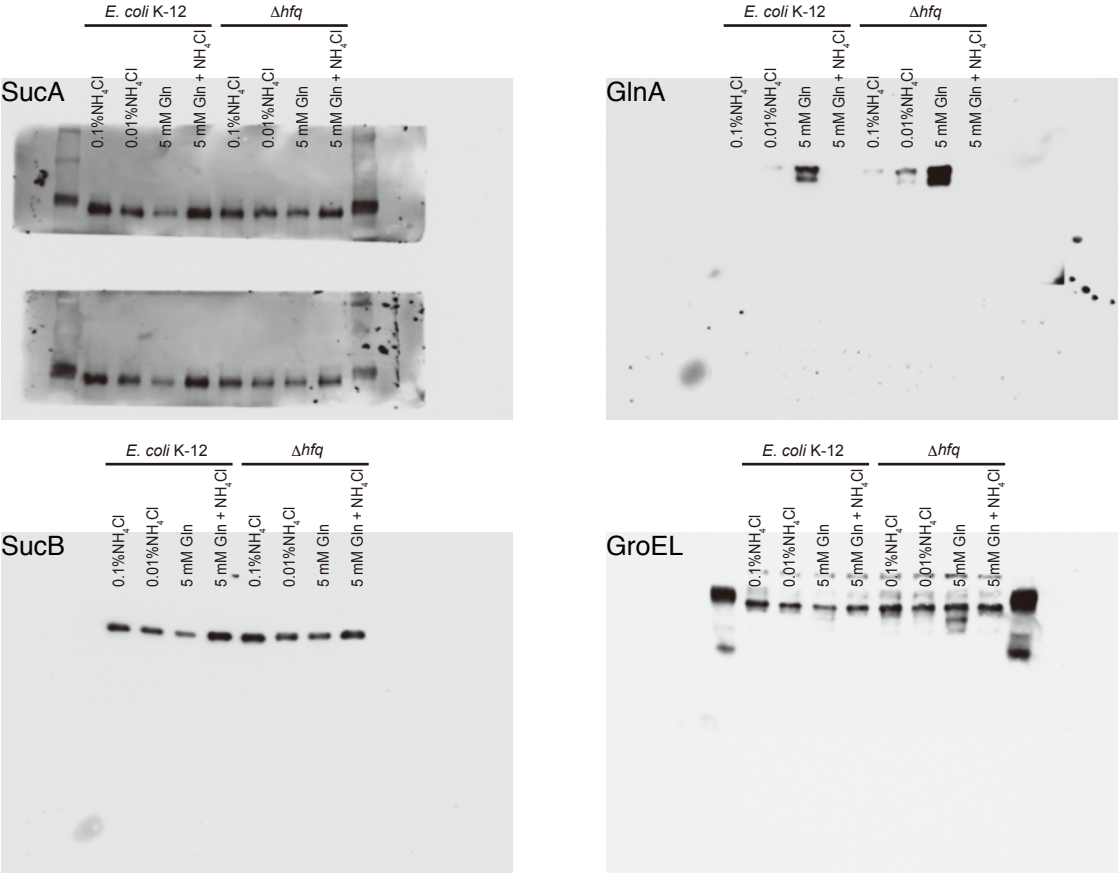

Supplement: Figure 5—source data 1. [file elife-82411-fig5-data1.pdf]

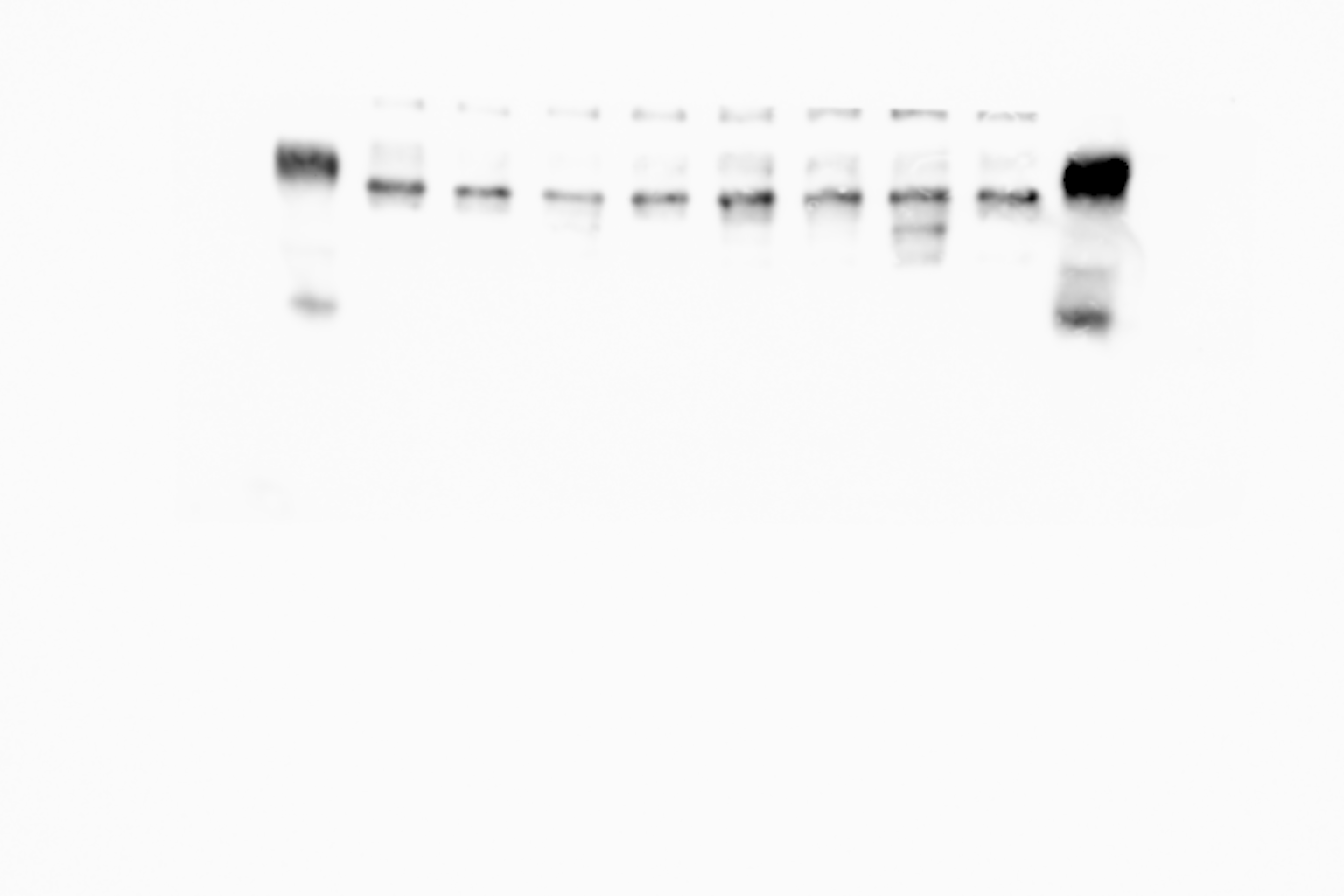

Supplement: Figure 5—source data 2. [file elife-82411-fig5-data2.zip › Fig5A_WB/20221003_1706groEL.tif]

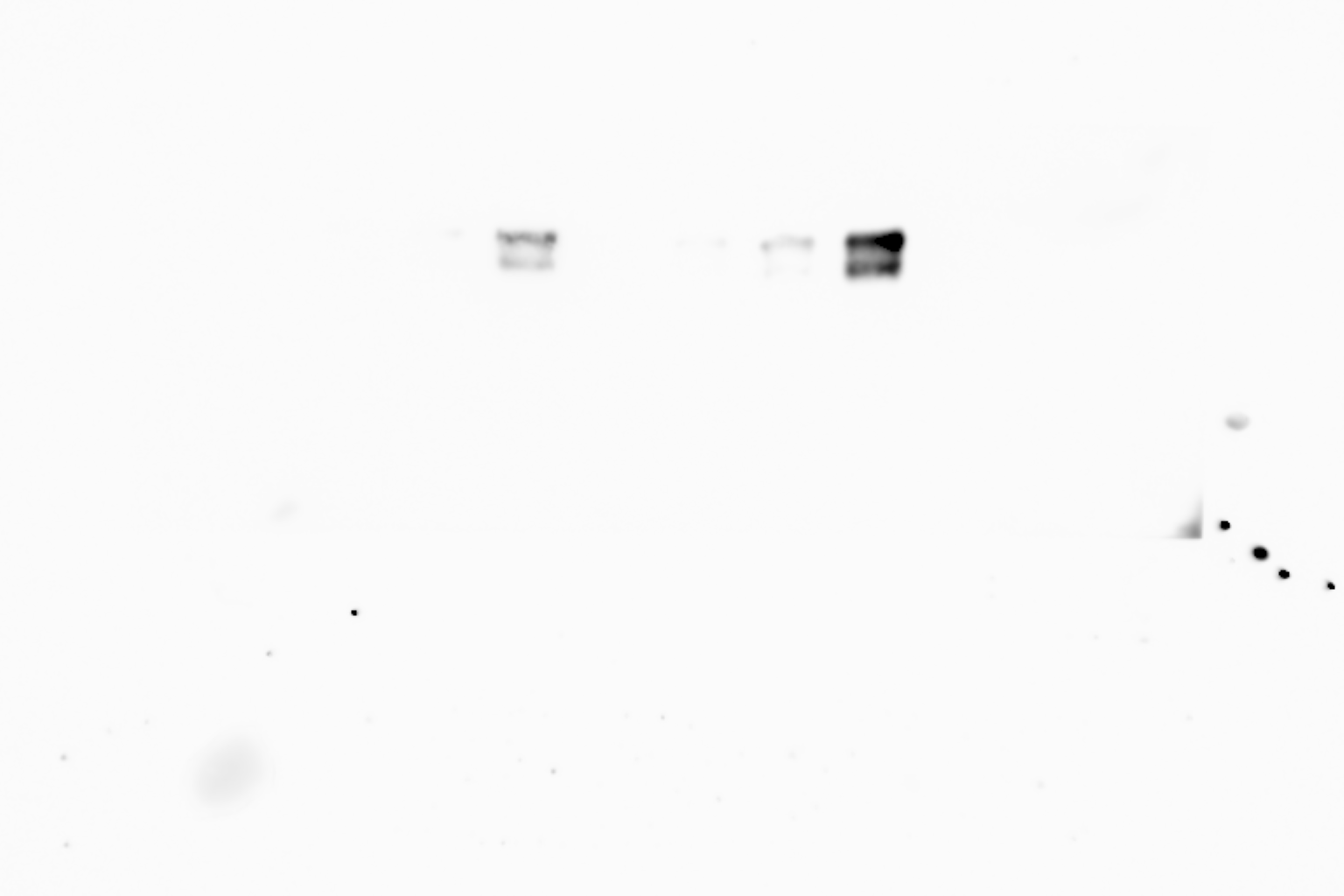

Supplement: Figure 5—source data 2. [file elife-82411-fig5-data2.zip › Fig5A_WB/20221001_1247glnA.tif]

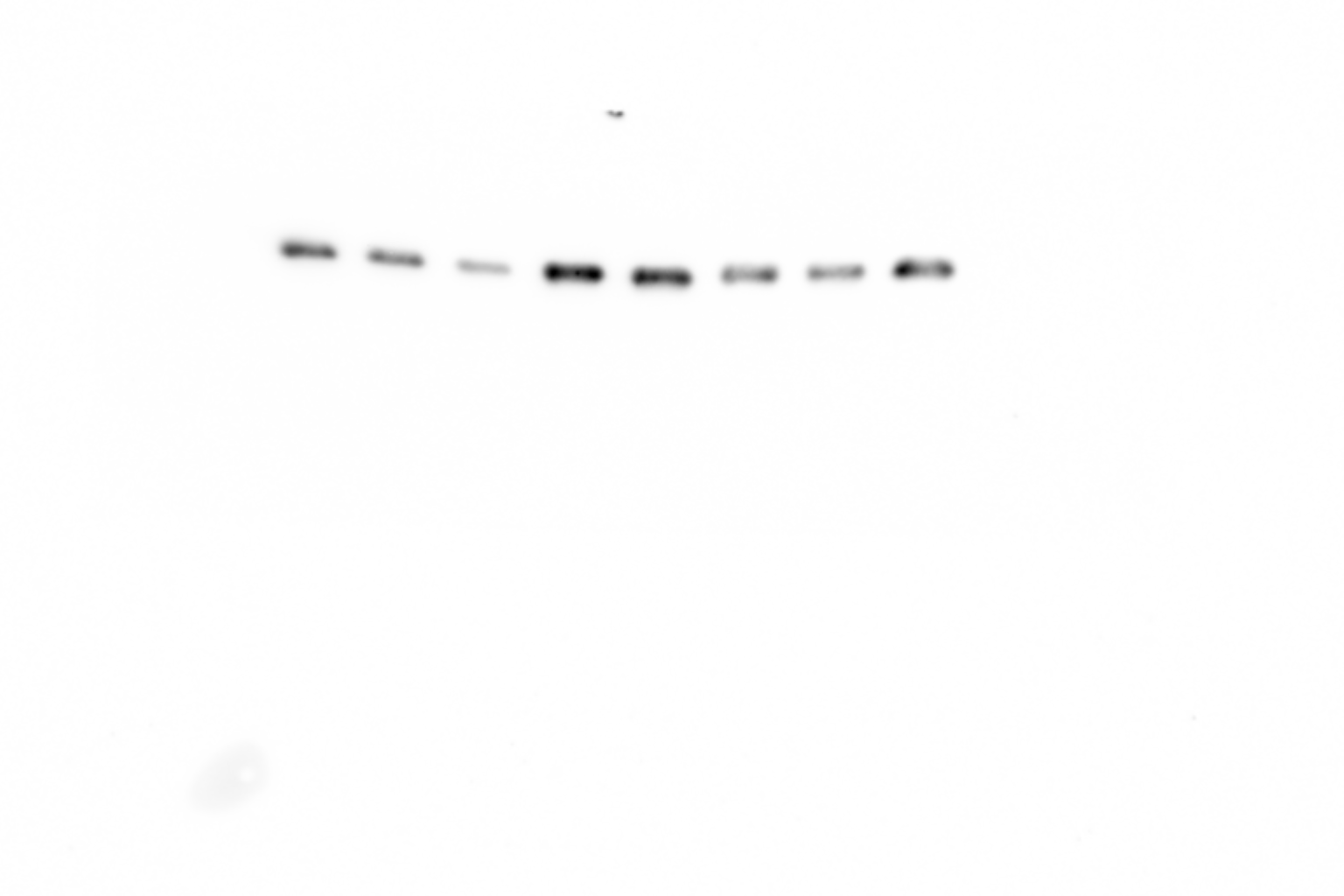

Supplement: Figure 5—source data 2. [file elife-82411-fig5-data2.zip › Fig5A_WB/20221001_1239sucB.tif]

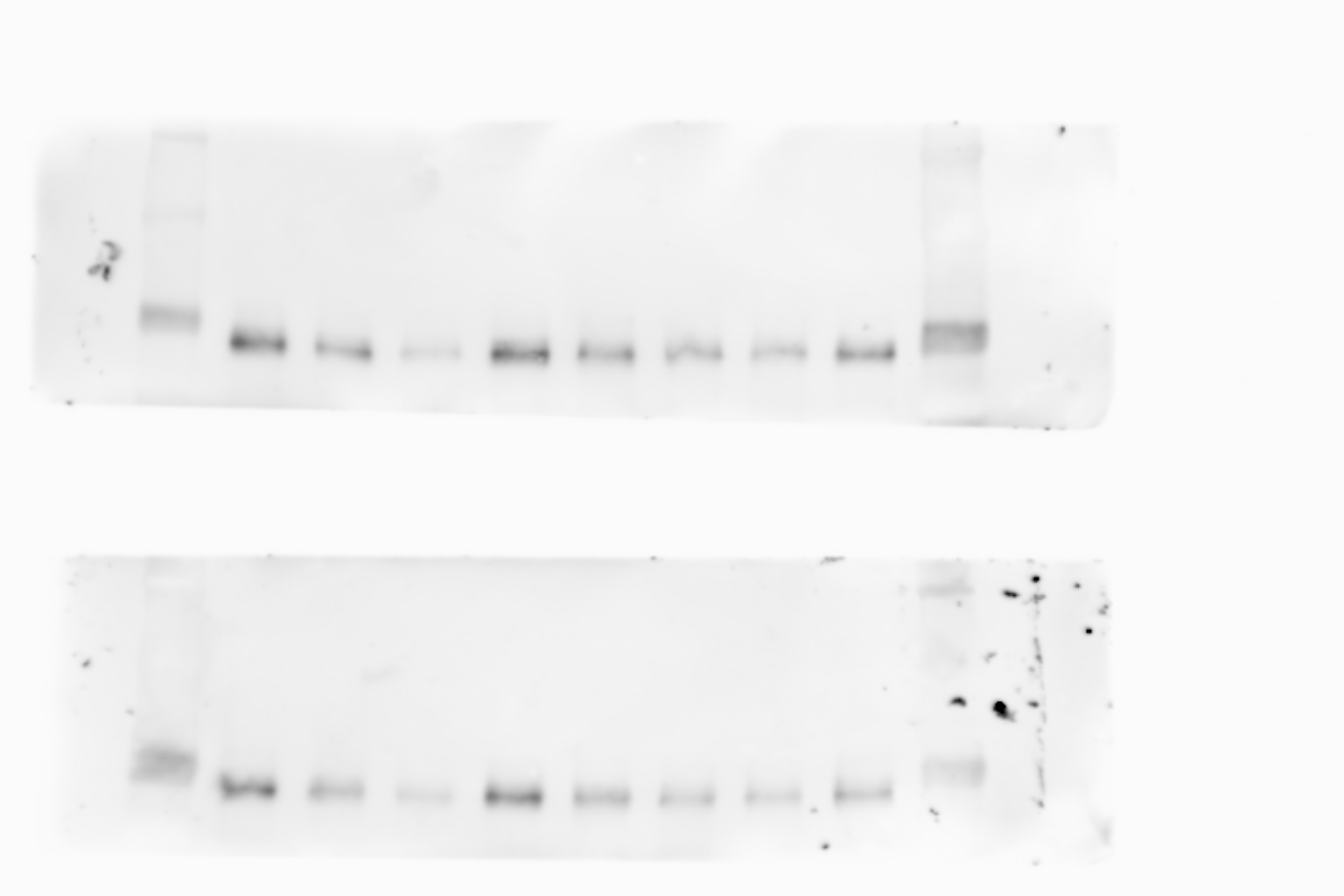

Supplement: Figure 5—source data 2. [file elife-82411-fig5-data2.zip › Fig5A_WB/20221001_1233sucA.tif]

GlnZ MMO-0416

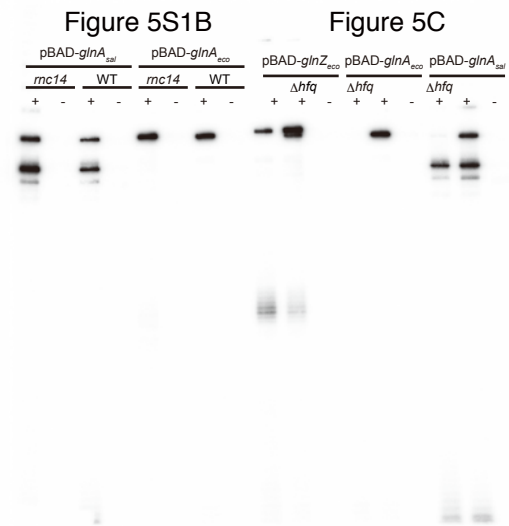

5S rRNA MMO-1056

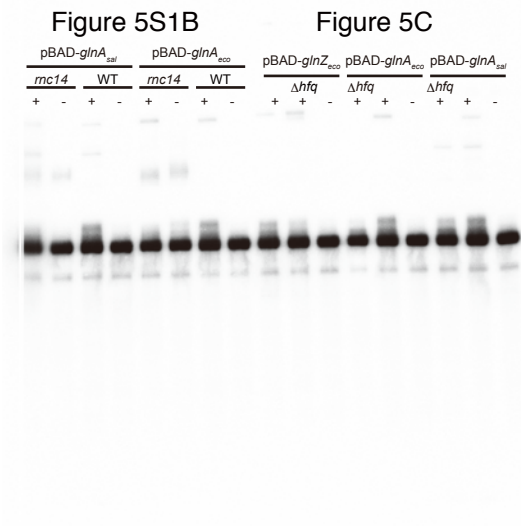

GlnZ MMO-0419

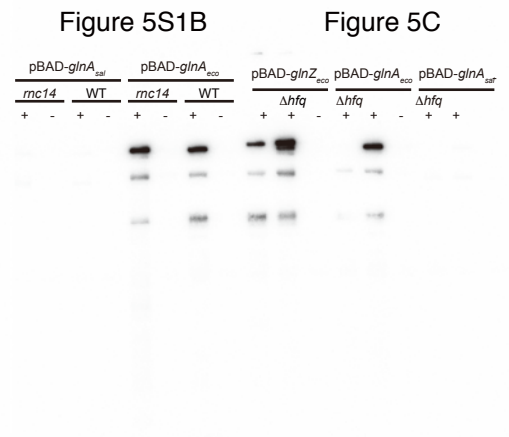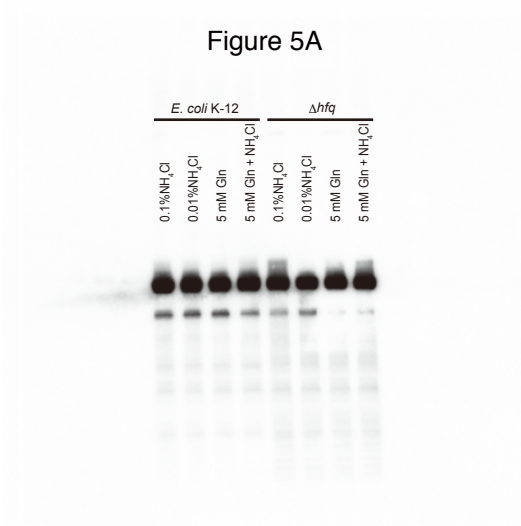

*glnA*

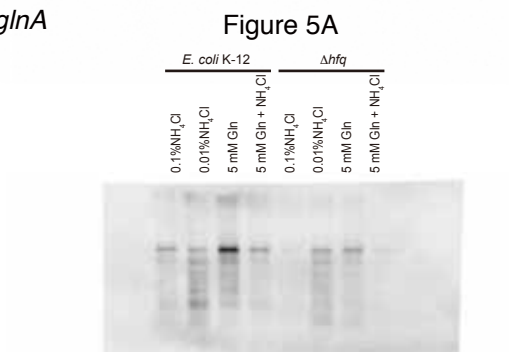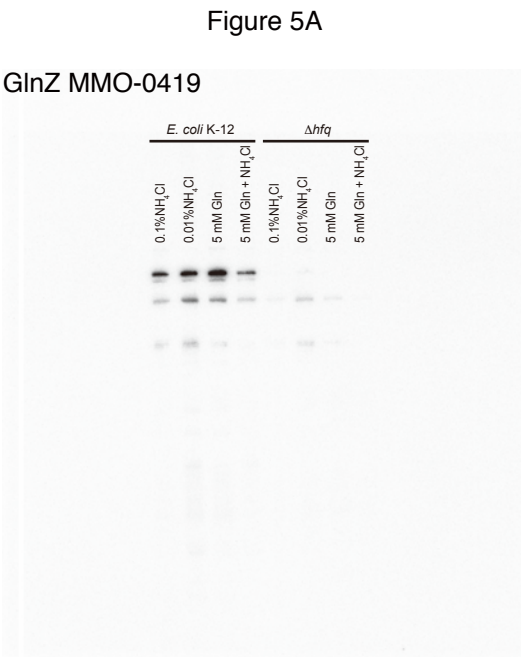

GlnZ MMO-0419

Supplement: Figure 5—source data 3. [file elife-82411-fig5-data3.pdf]

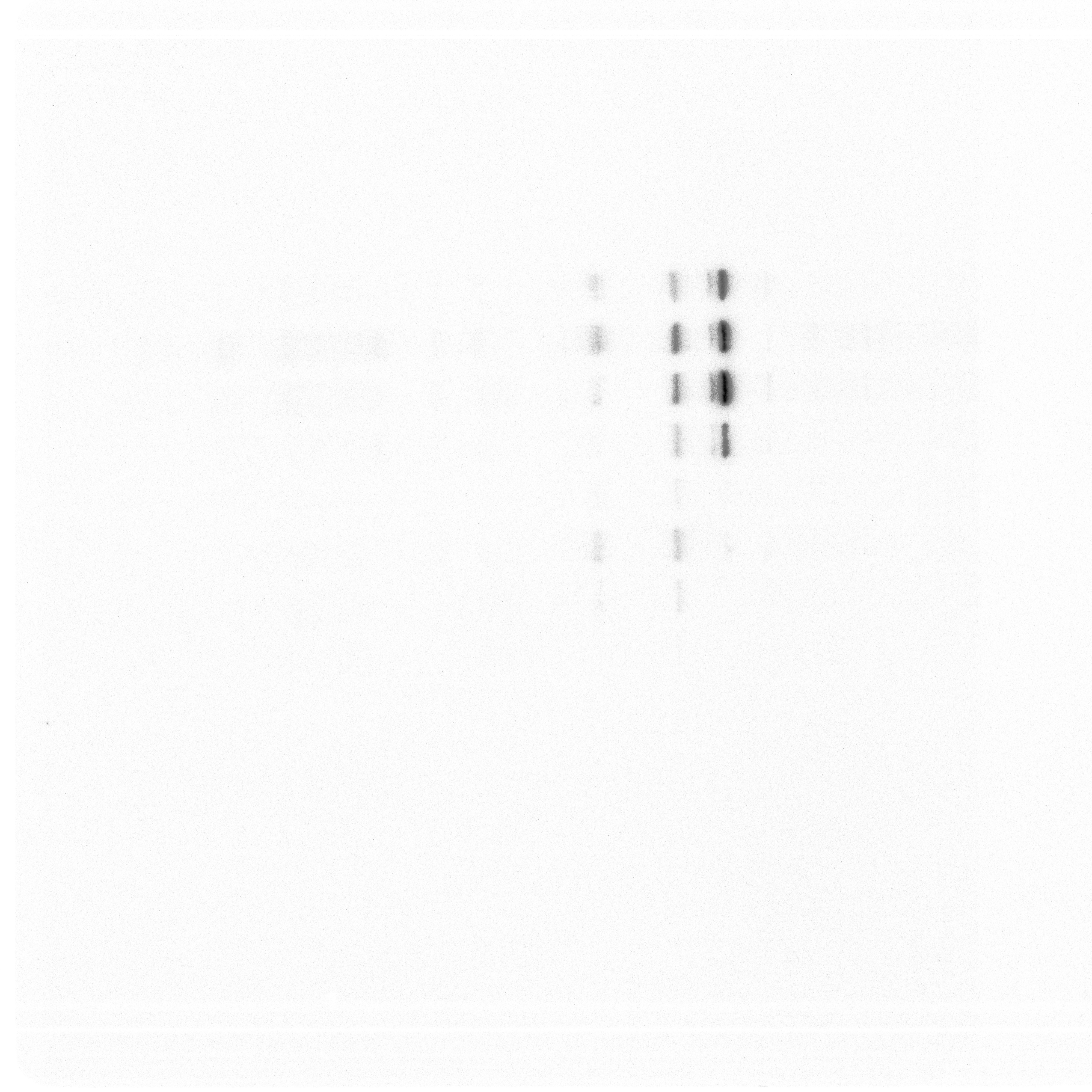

Supplement: Figure 5—source data 4. [file elife-82411-fig5-data4.zip › Fig5AC_NB/Fig5A_MMO-0419.tif]

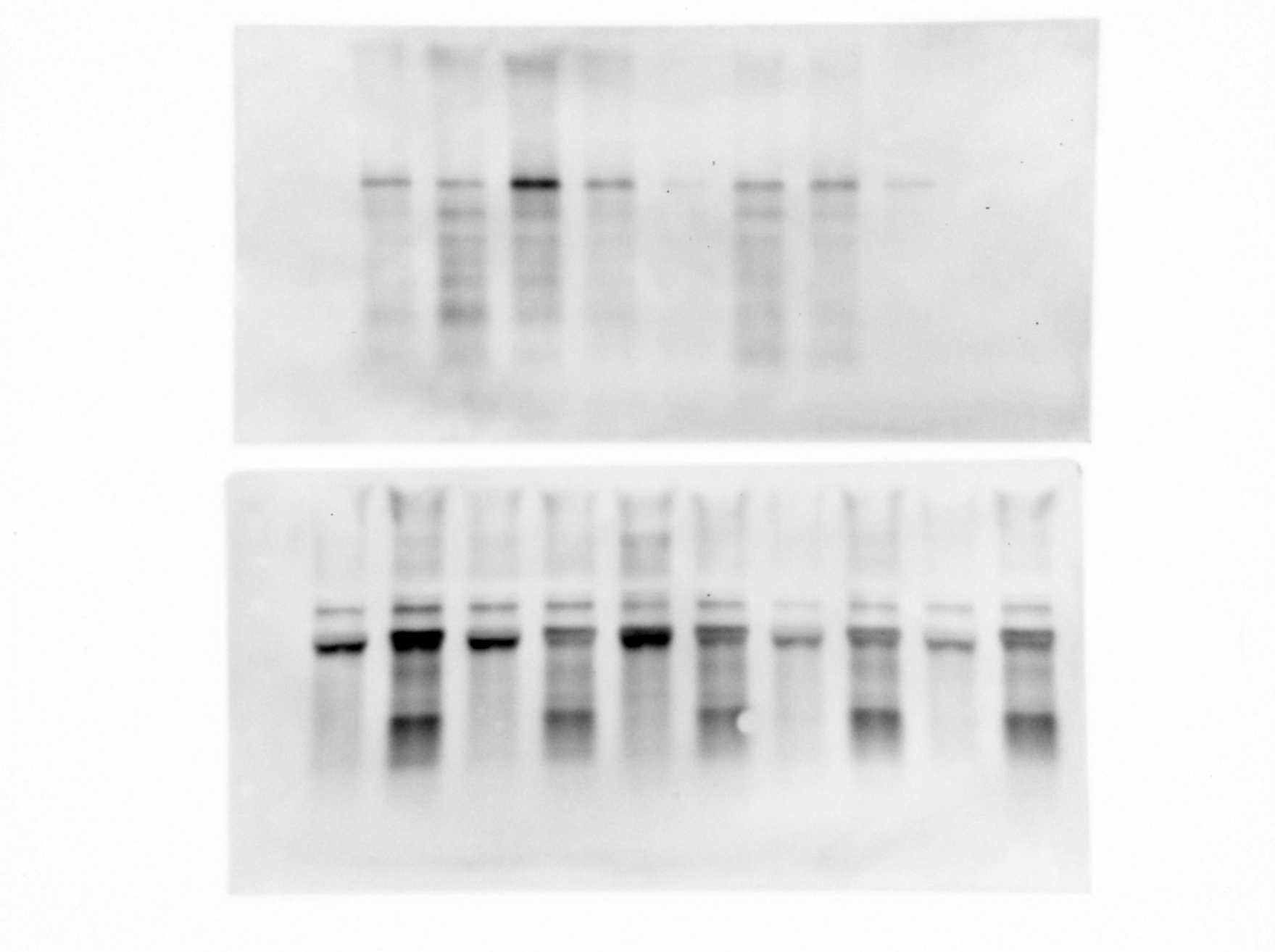

Supplement: Figure 5—source data 4. [file elife-82411-fig5-data4.zip › Fig5AC_NB/Fig5A_glnA.tif]

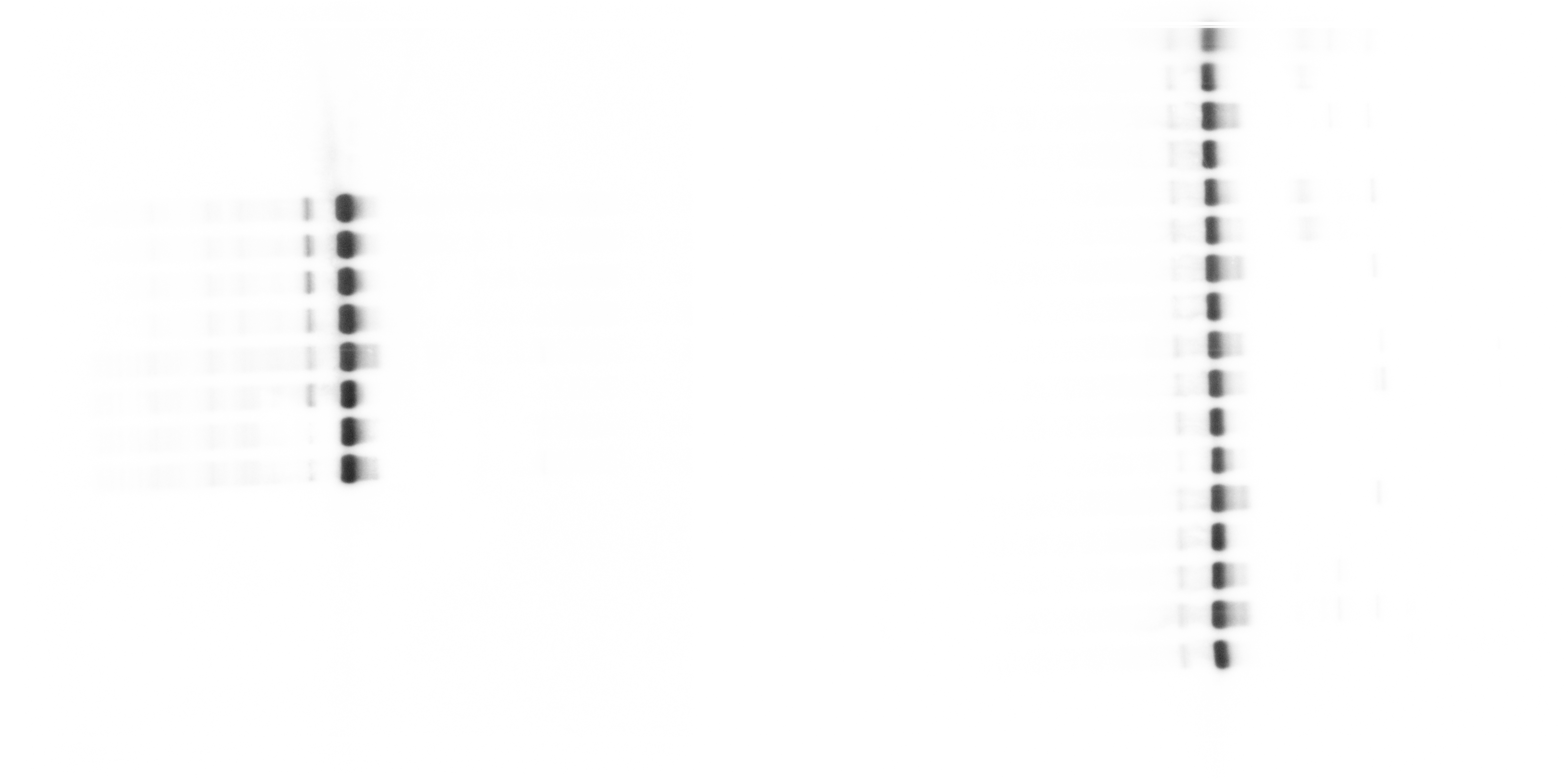

Supplement: Figure 5—source data 4. [file elife-82411-fig5-data4.zip › Fig5AC_NB/Fig5A_MMO-1056.tif]

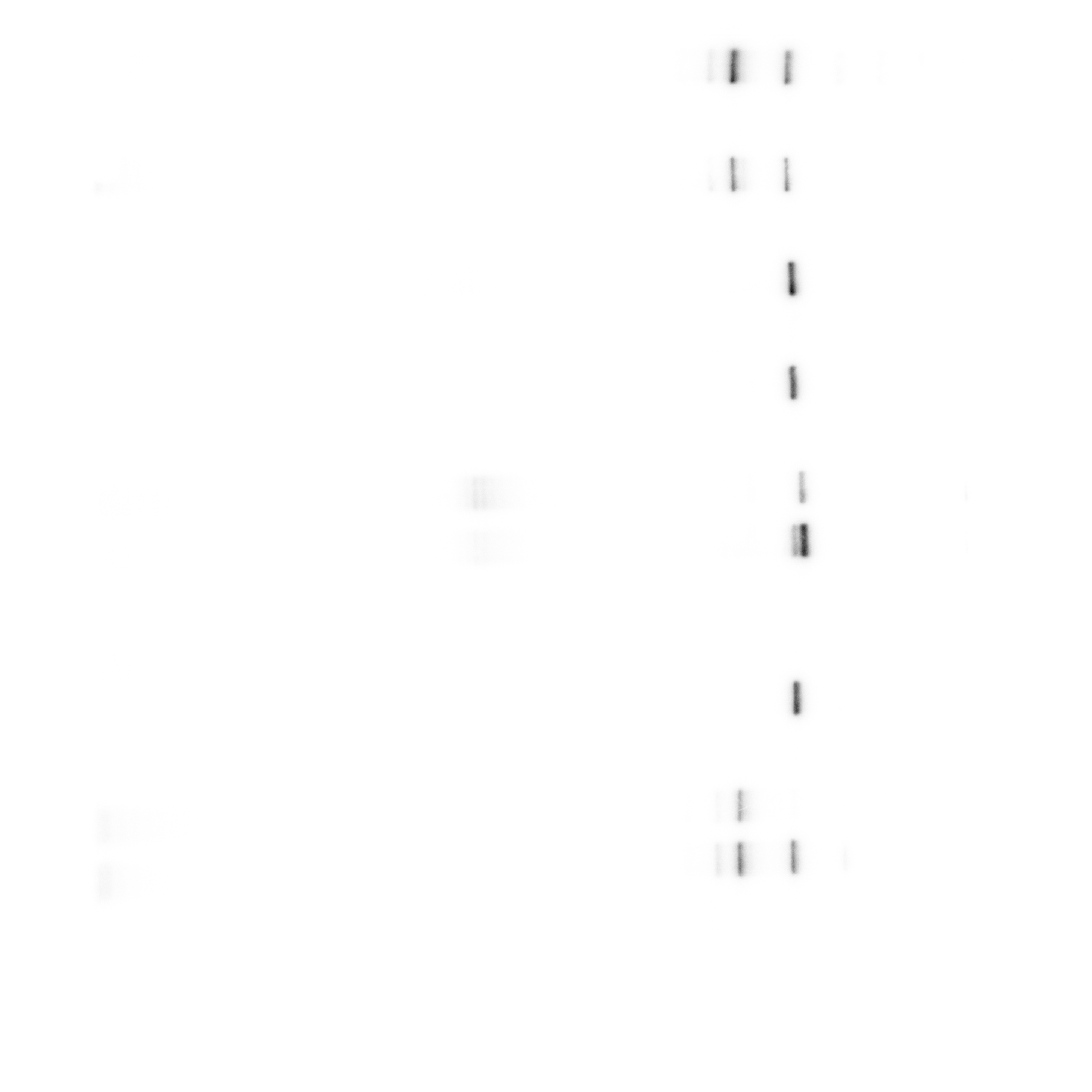

Supplement: Figure 5—source data 4. [file elife-82411-fig5-data4.zip › Fig5AC_NB/Fig5A_MMO-0416.tif]

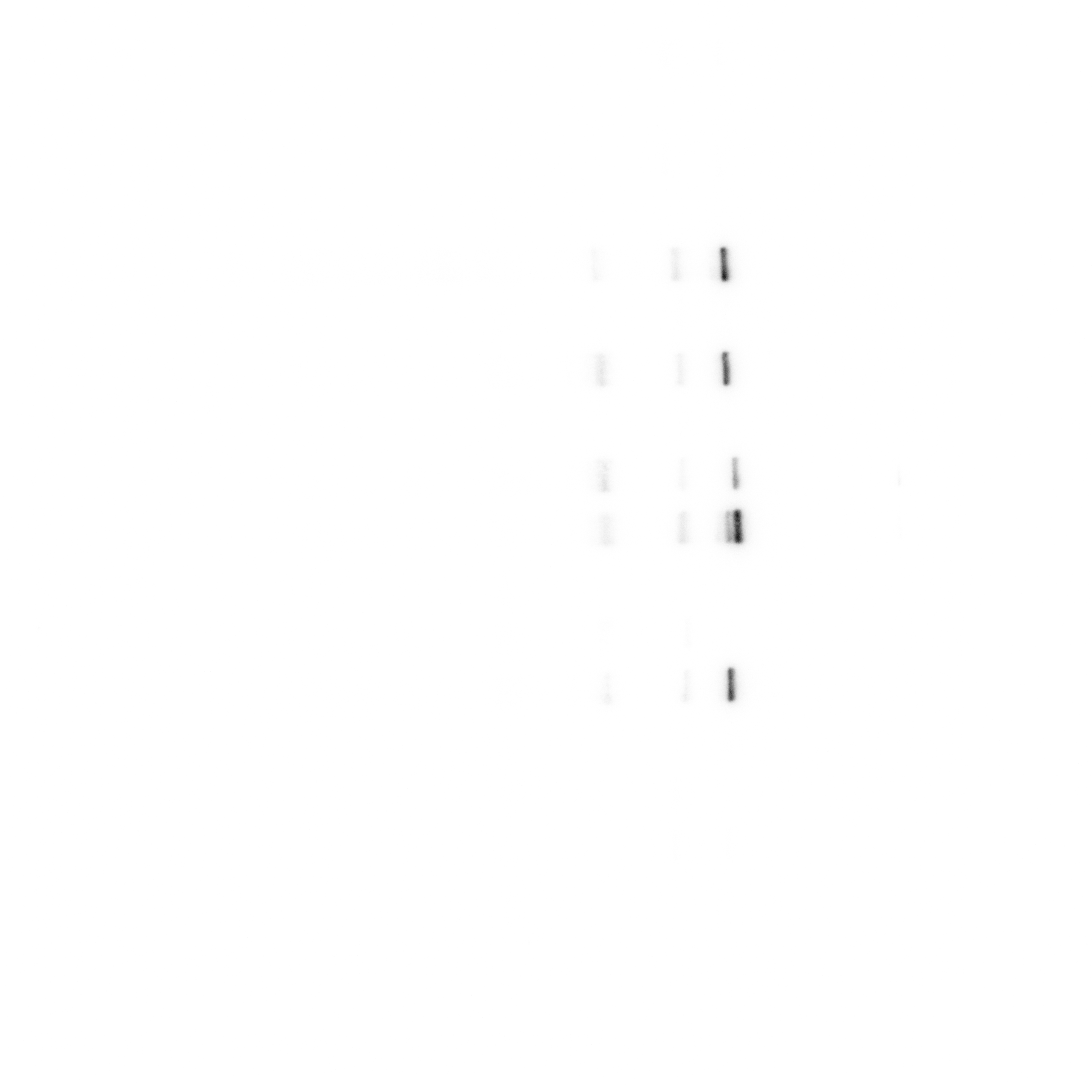

Supplement: Figure 5—source data 4. [file elife-82411-fig5-data4.zip › Fig5AC_NB/Fig5C_MMO-0419.tif]

Figure 5B

SucA

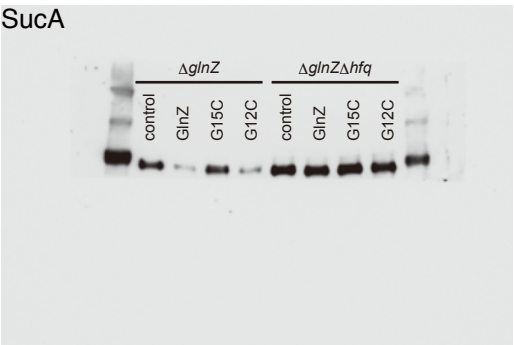

AceE

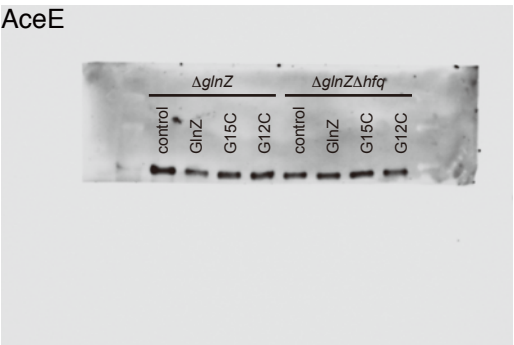

SucB

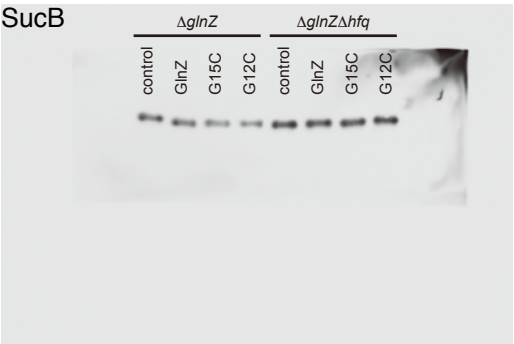

GroEL

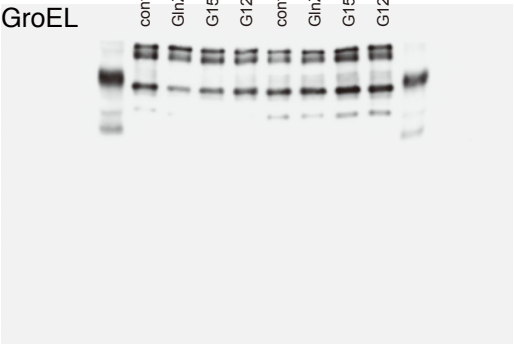

GlnZ MMO-0419

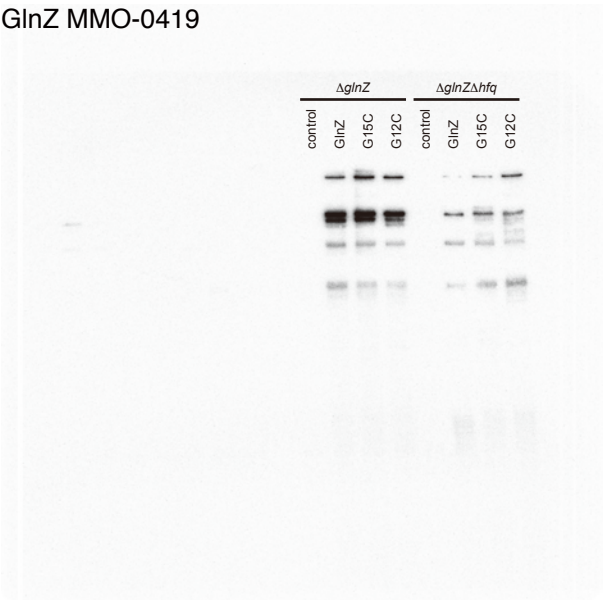

5S rRNA MMO-1056

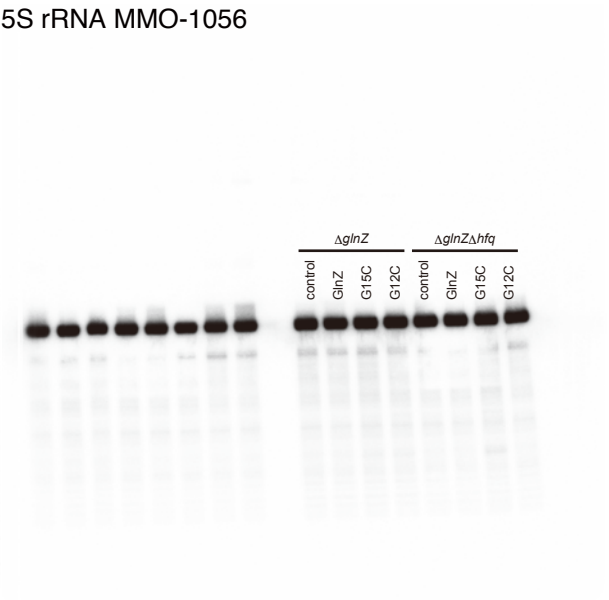

Supplement: Figure 5—source data 5. [file elife-82411-fig5-data5.pdf]

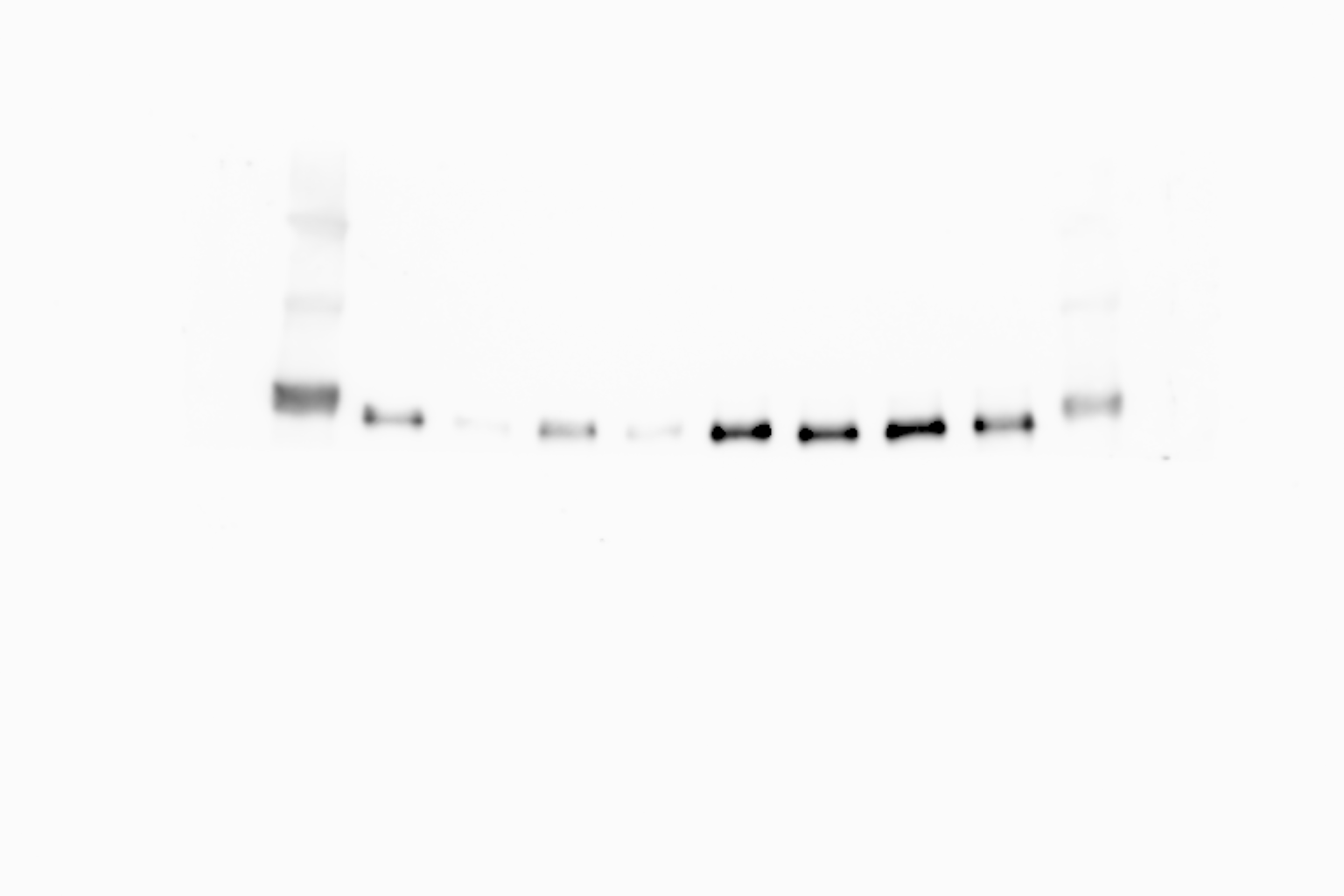

Supplement: Figure 5—source data 6. [file elife-82411-fig5-data6.zip › Fig5B/20221011_1537pPL_sucA.tif]

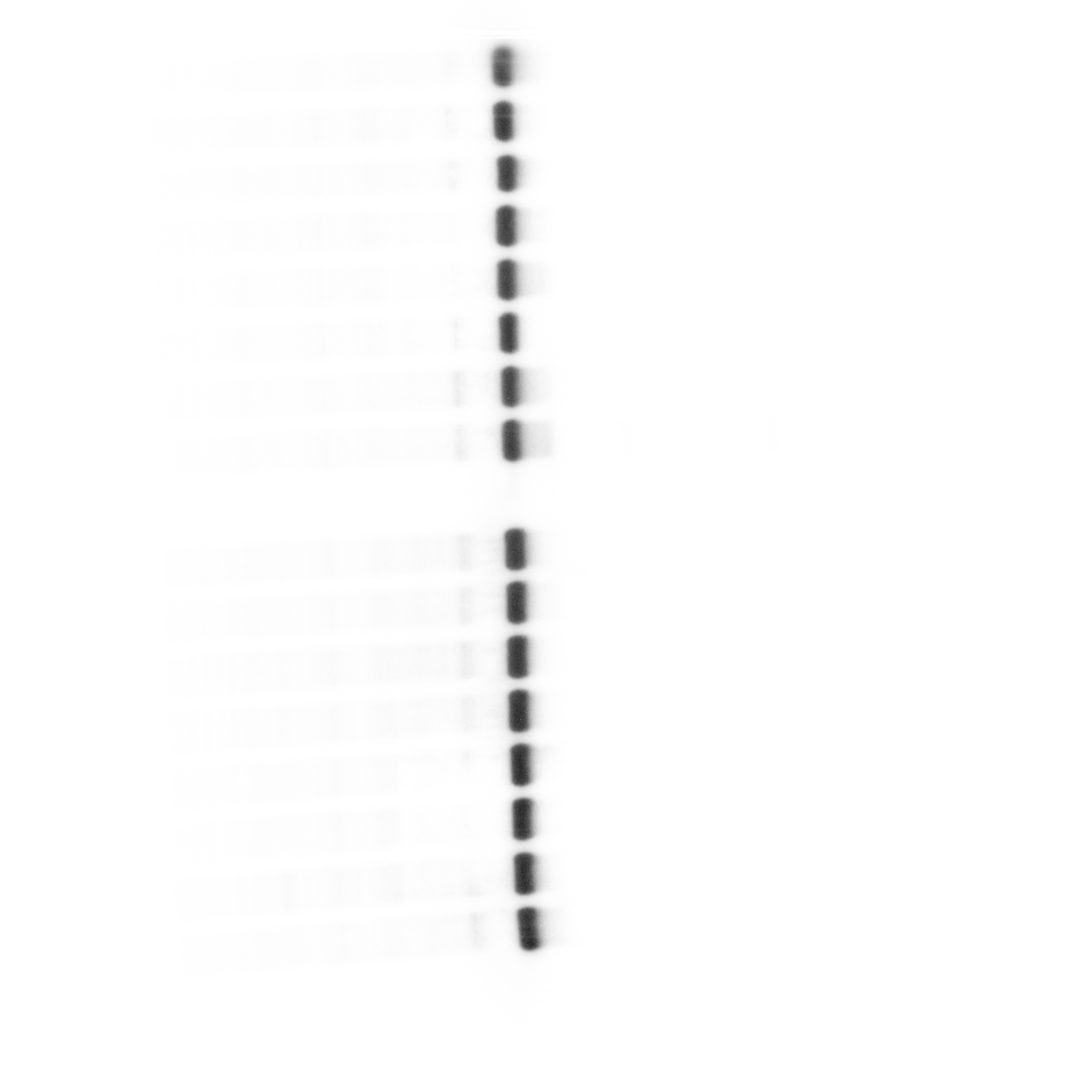

Supplement: Figure 5—source data 6. [file elife-82411-fig5-data6.zip › Fig5B/20221023_MMO1056-[Phosphor].tif]

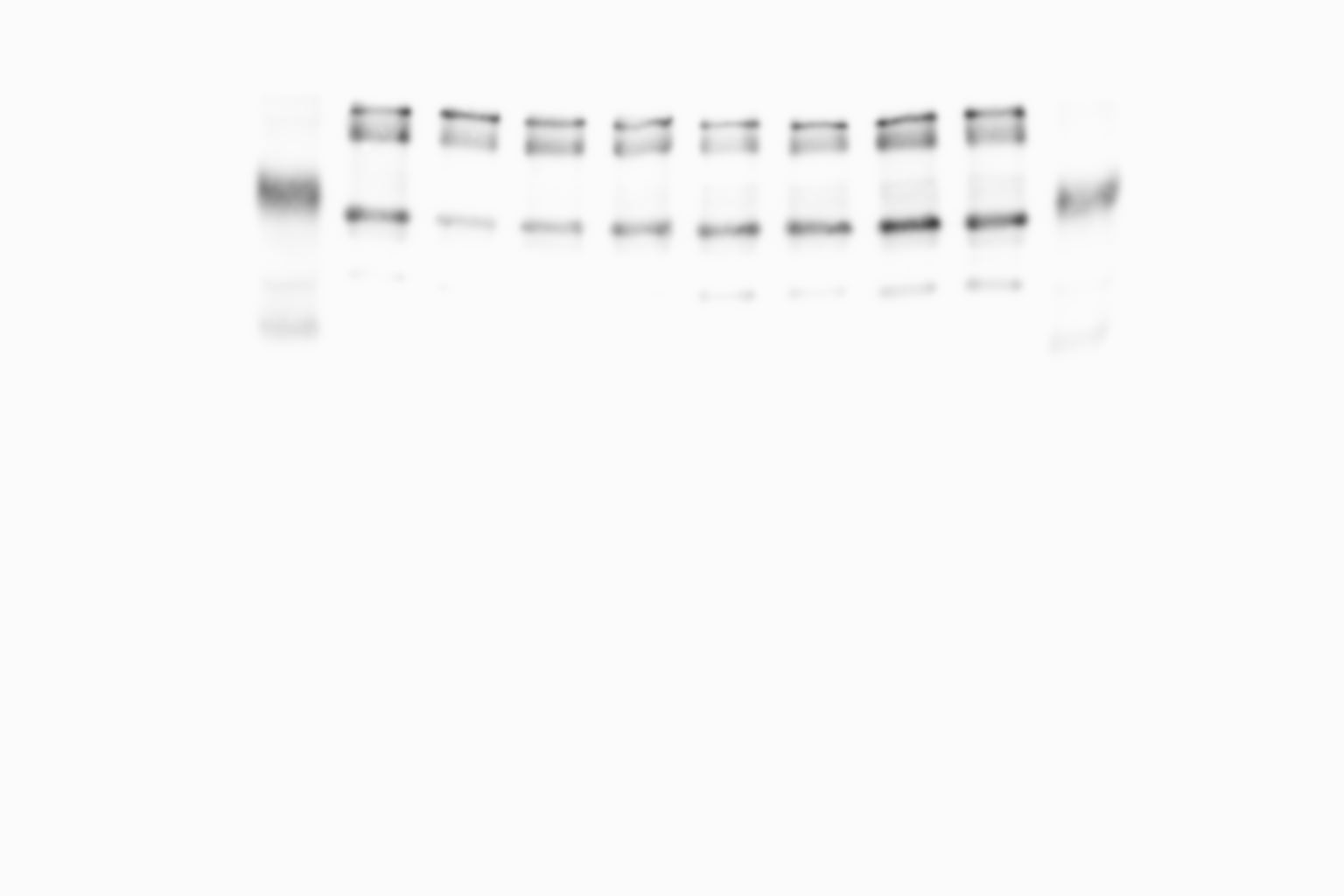

Supplement: Figure 5—source data 6. [file elife-82411-fig5-data6.zip › Fig5B/20221011_1549pPL_groEL.tif]

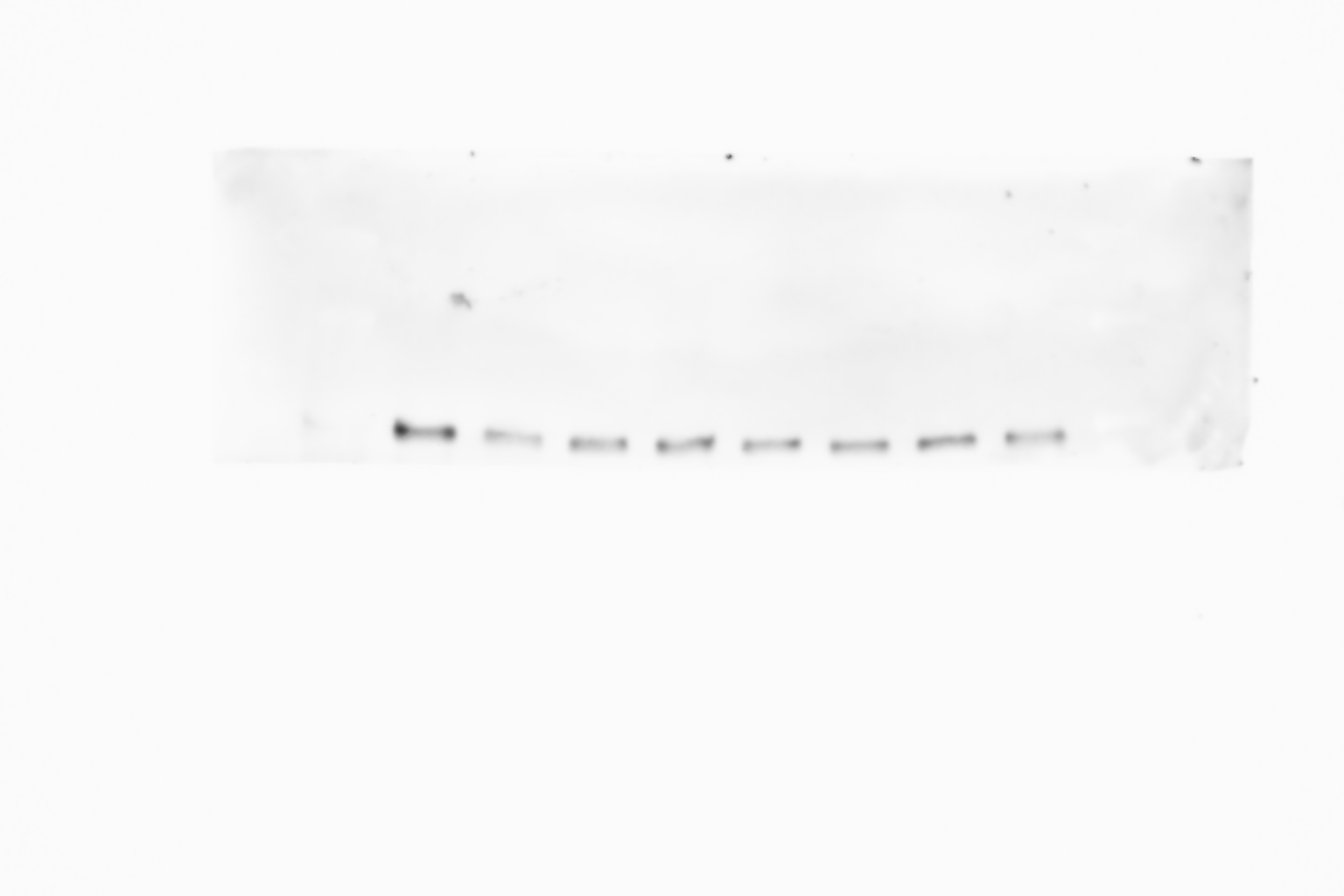

Supplement: Figure 5—source data 6. [file elife-82411-fig5-data6.zip › Fig5B/20221007_1120pPL_aceE.tif]

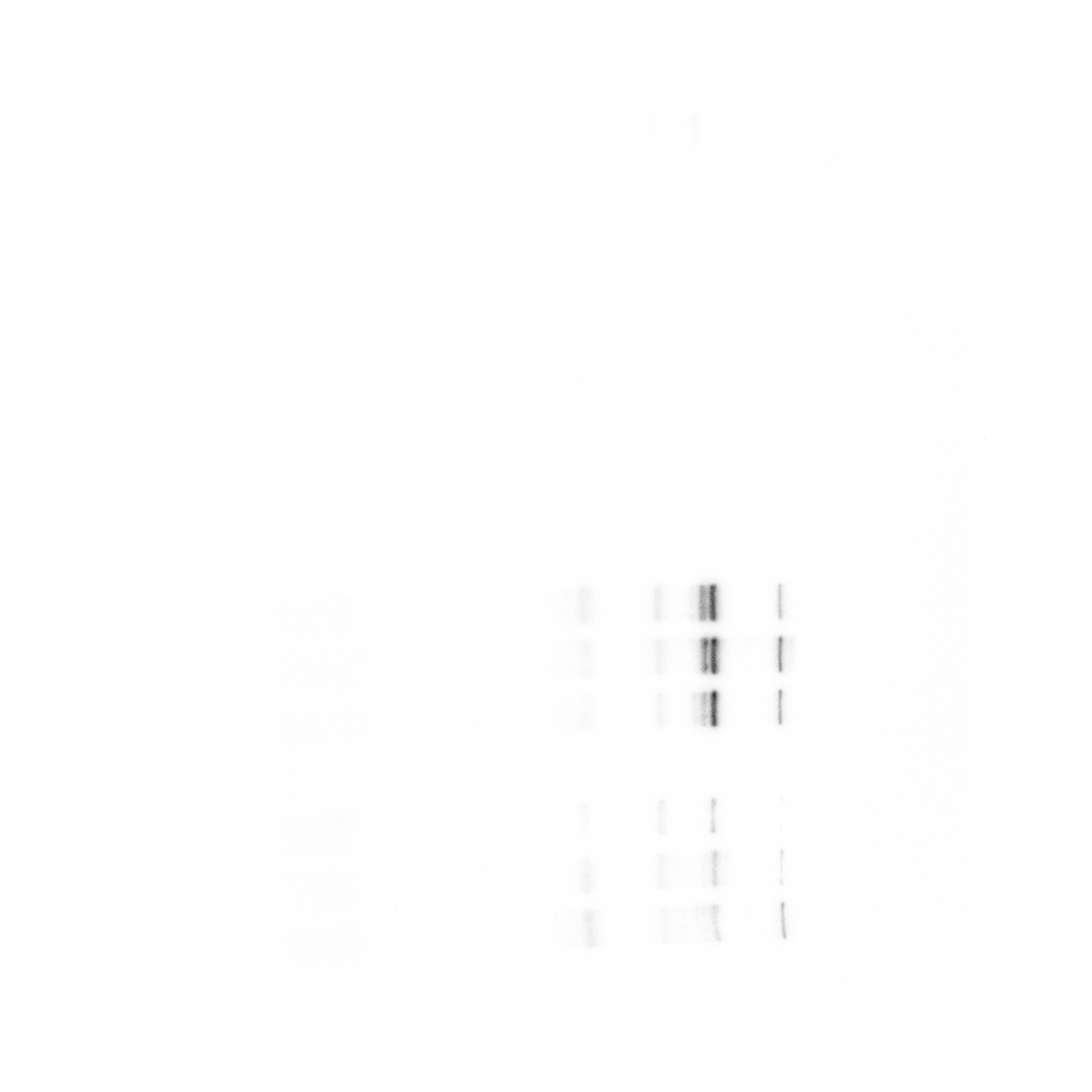

Supplement: Figure 5—source data 6. [file elife-82411-fig5-data6.zip › Fig5B/20221020_MMO0419-[Phosphor].tif]

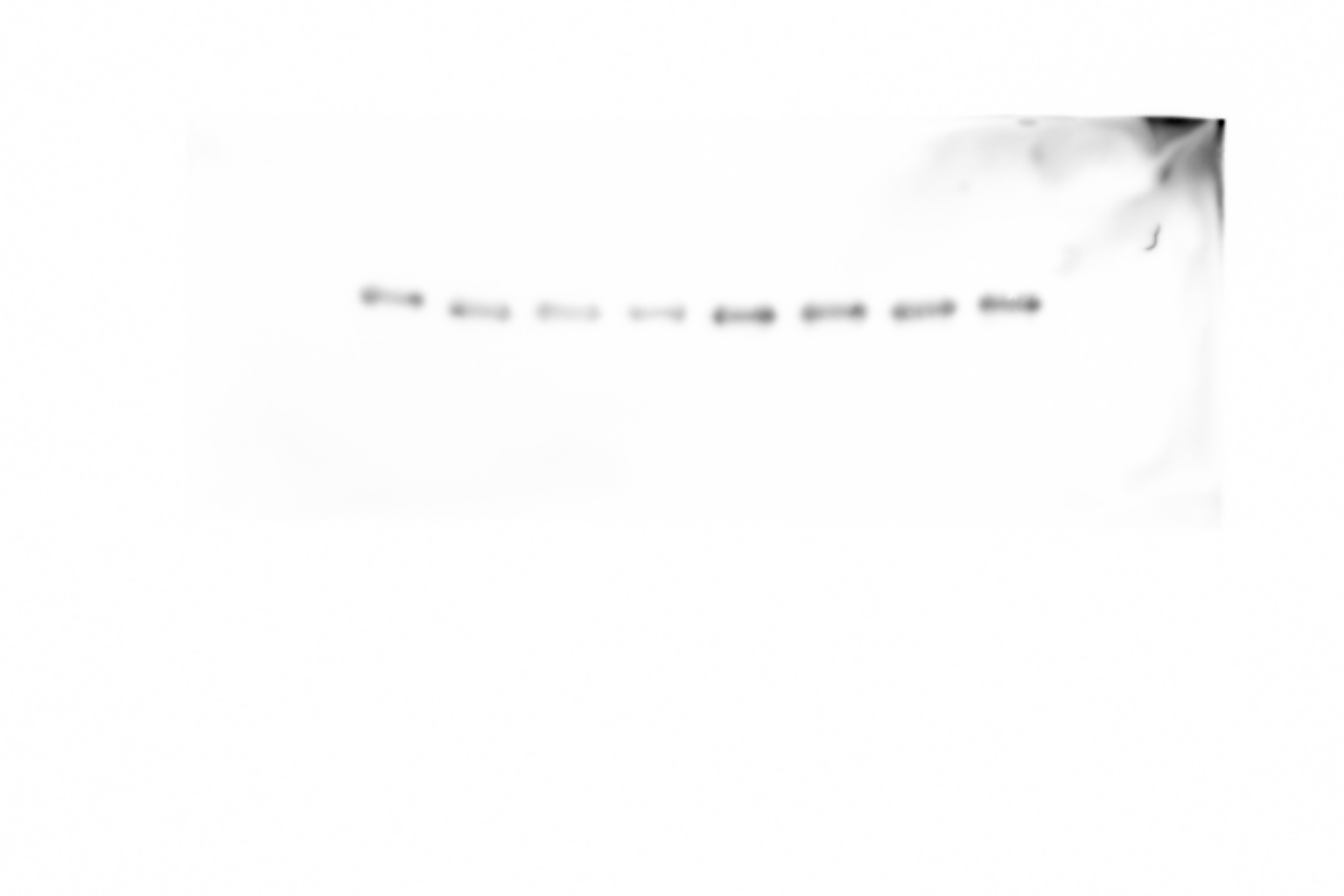

Supplement: Figure 5—source data 6. [file elife-82411-fig5-data6.zip › Fig5B/20221007_1134pPL_sucB.tif]

AceE

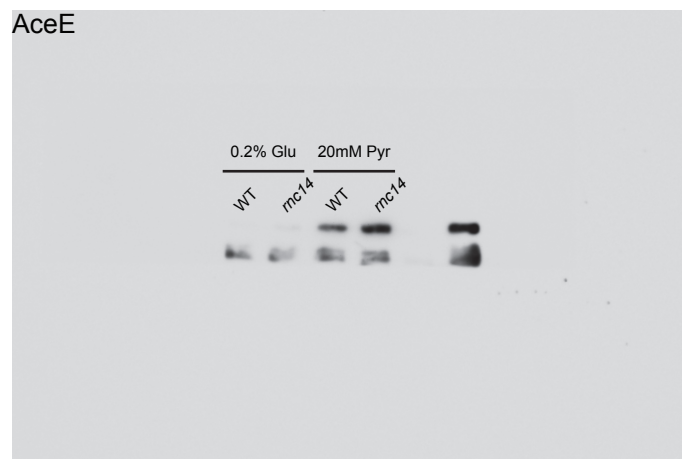

SucA

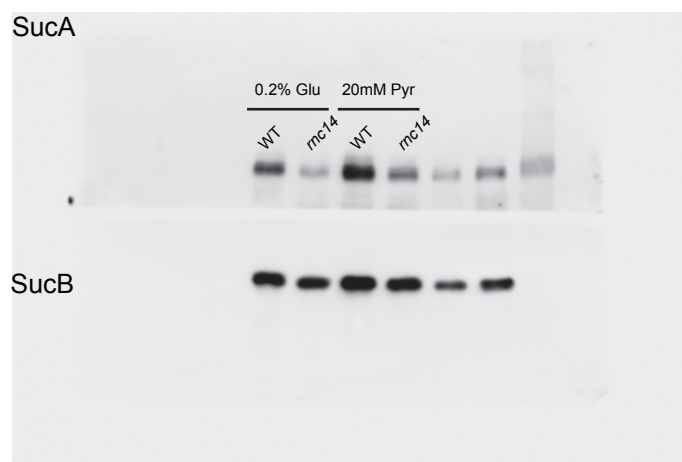

SucB

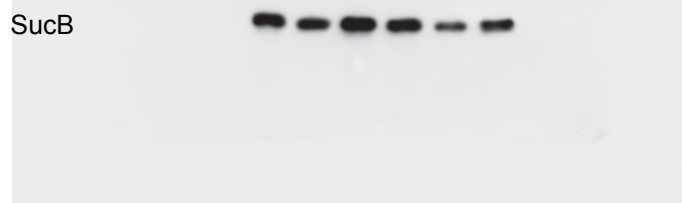

GroEL

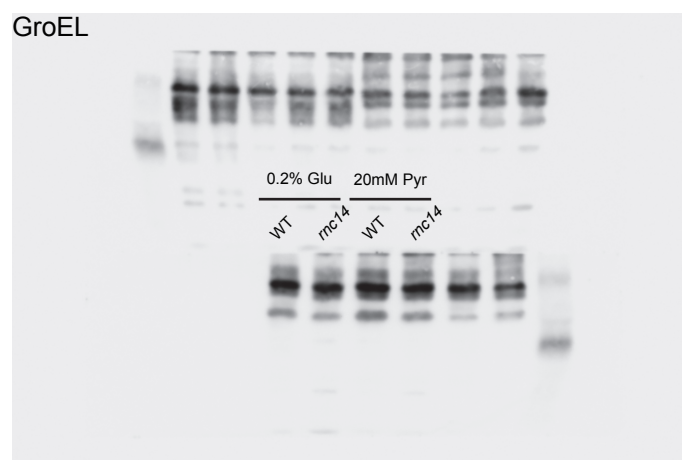

Supplement: Figure 5—source data 7. [file elife-82411-fig5-data7.pdf]

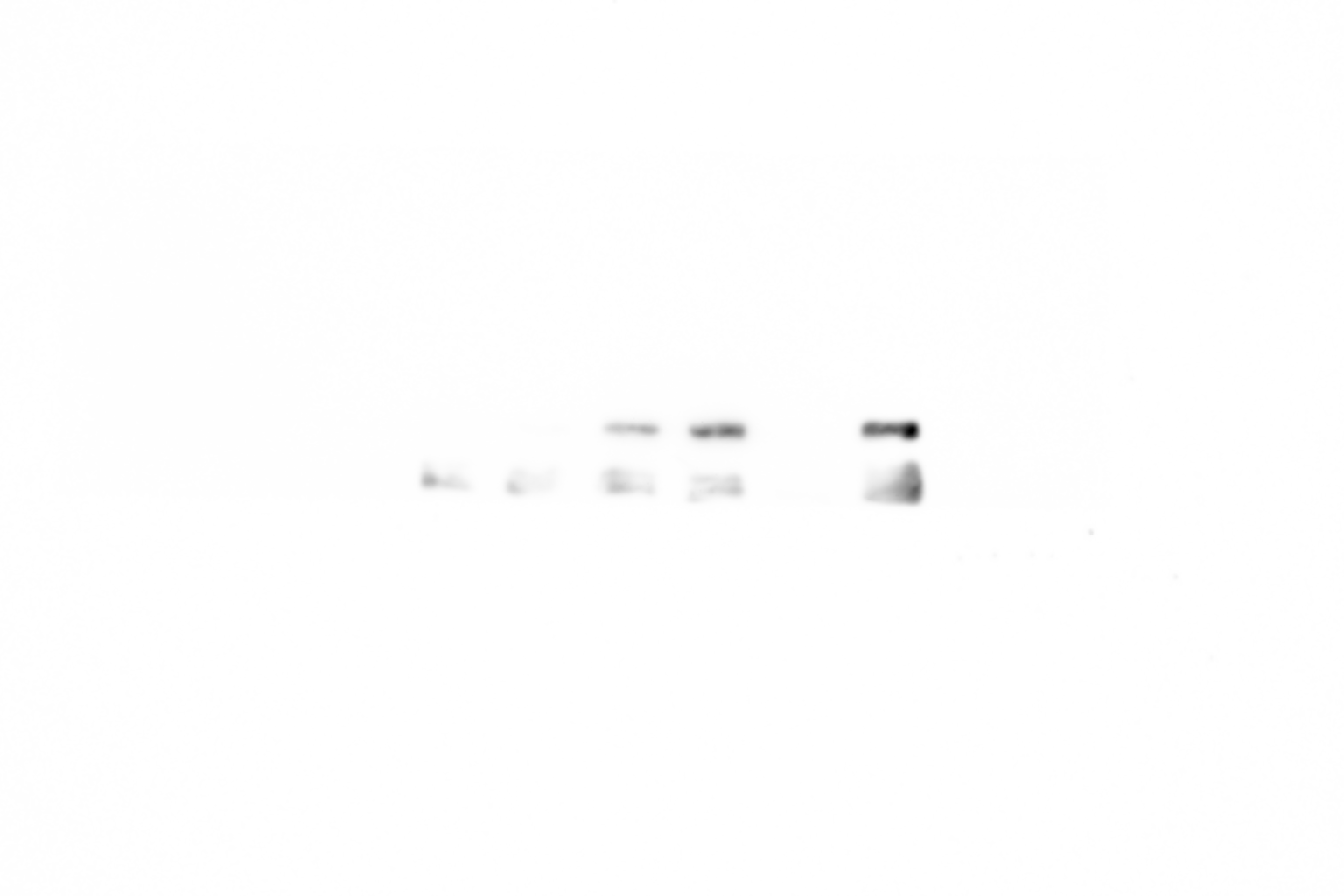

Supplement: Figure 5—source data 8. [file elife-82411-fig5-data8.zip › FigS3_source/FigS3_AceE.tif]

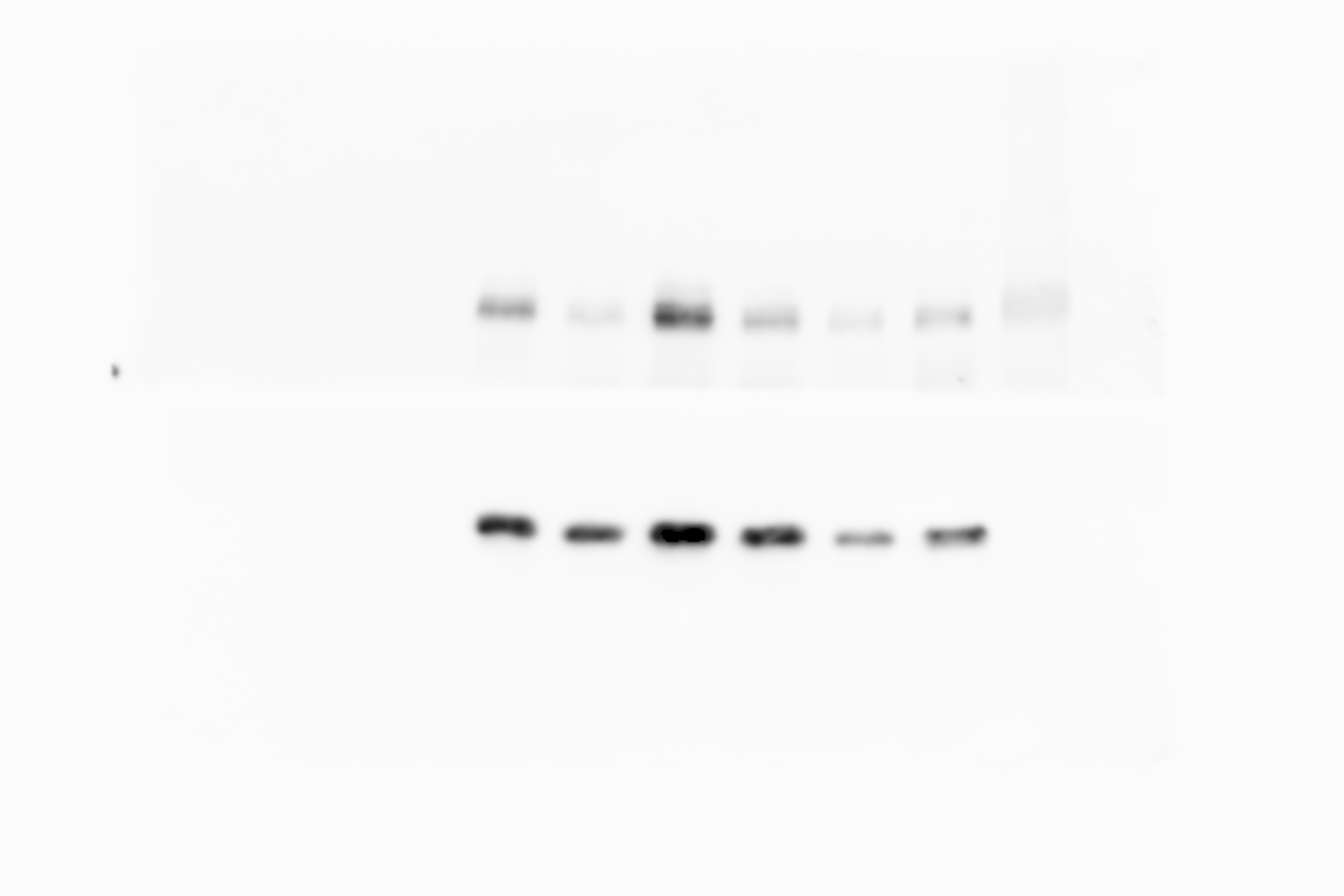

Supplement: Figure 5—source data 8. [file elife-82411-fig5-data8.zip › FigS3_source/FigS3_SucAB.tif]

Figure 6A

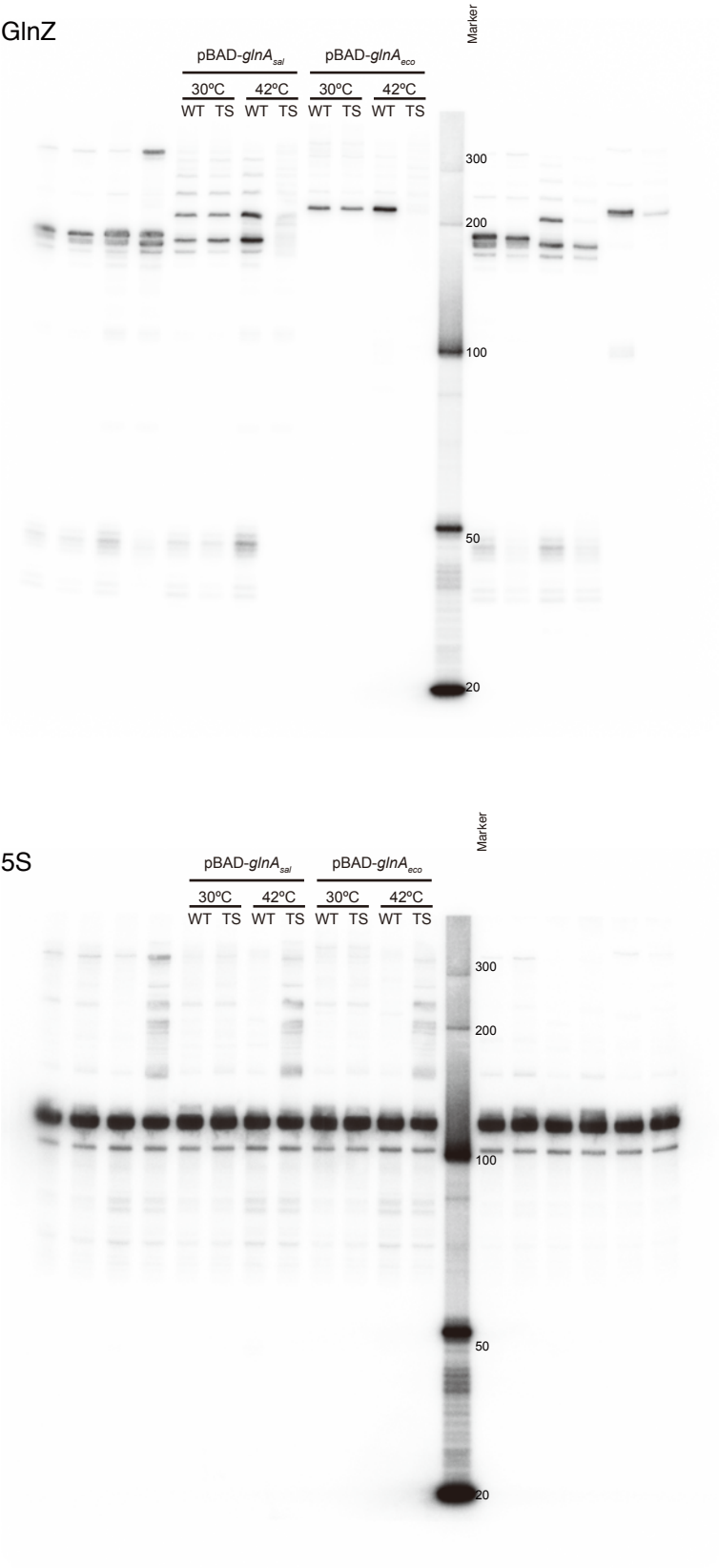

Supplement: Figure 6—source data 1. [file elife-82411-fig6-data1.pdf]

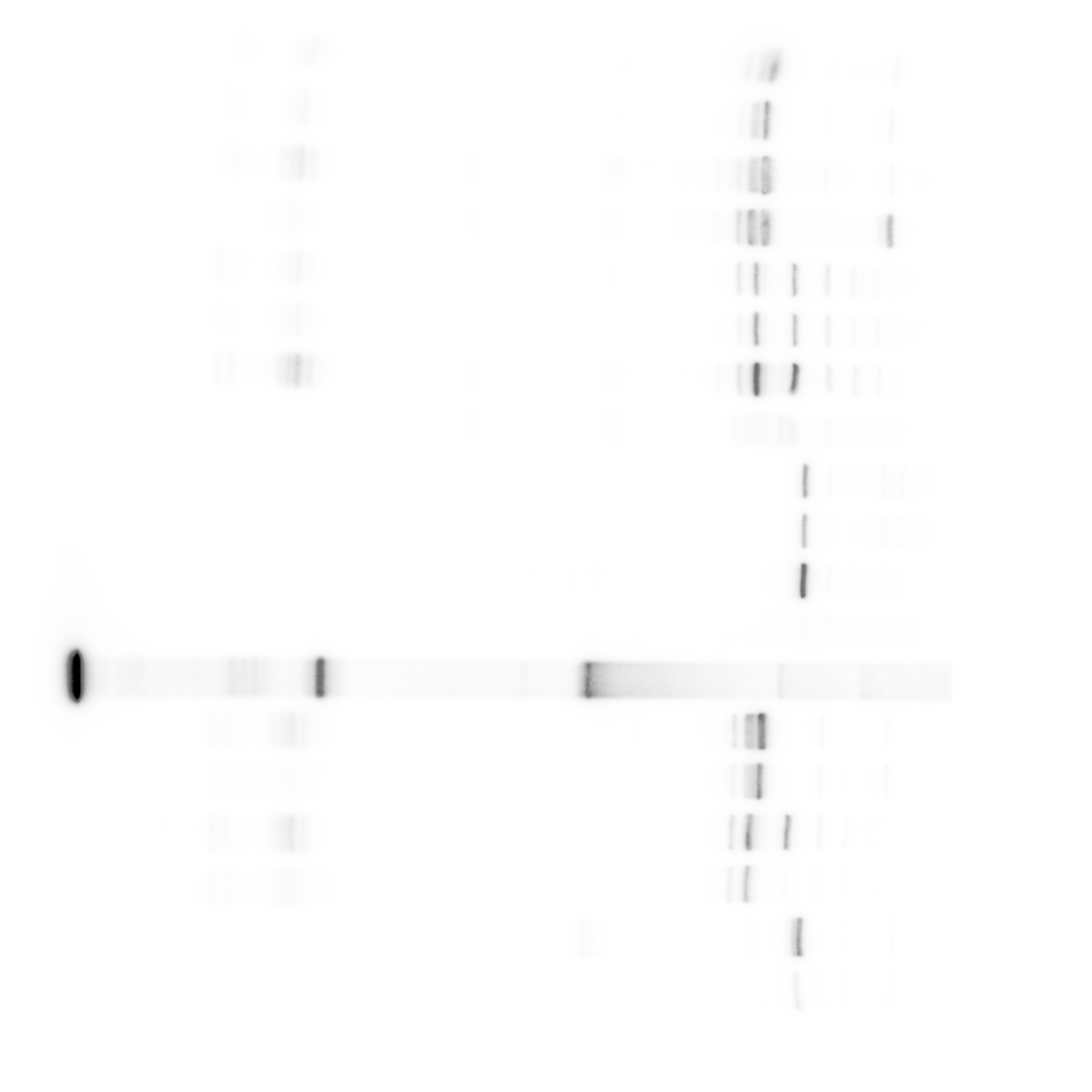

Supplement: Figure 6—source data 2. [file elife-82411-fig6-data2.zip › Fig6A_source/Figure6A_GlnZ.tif]

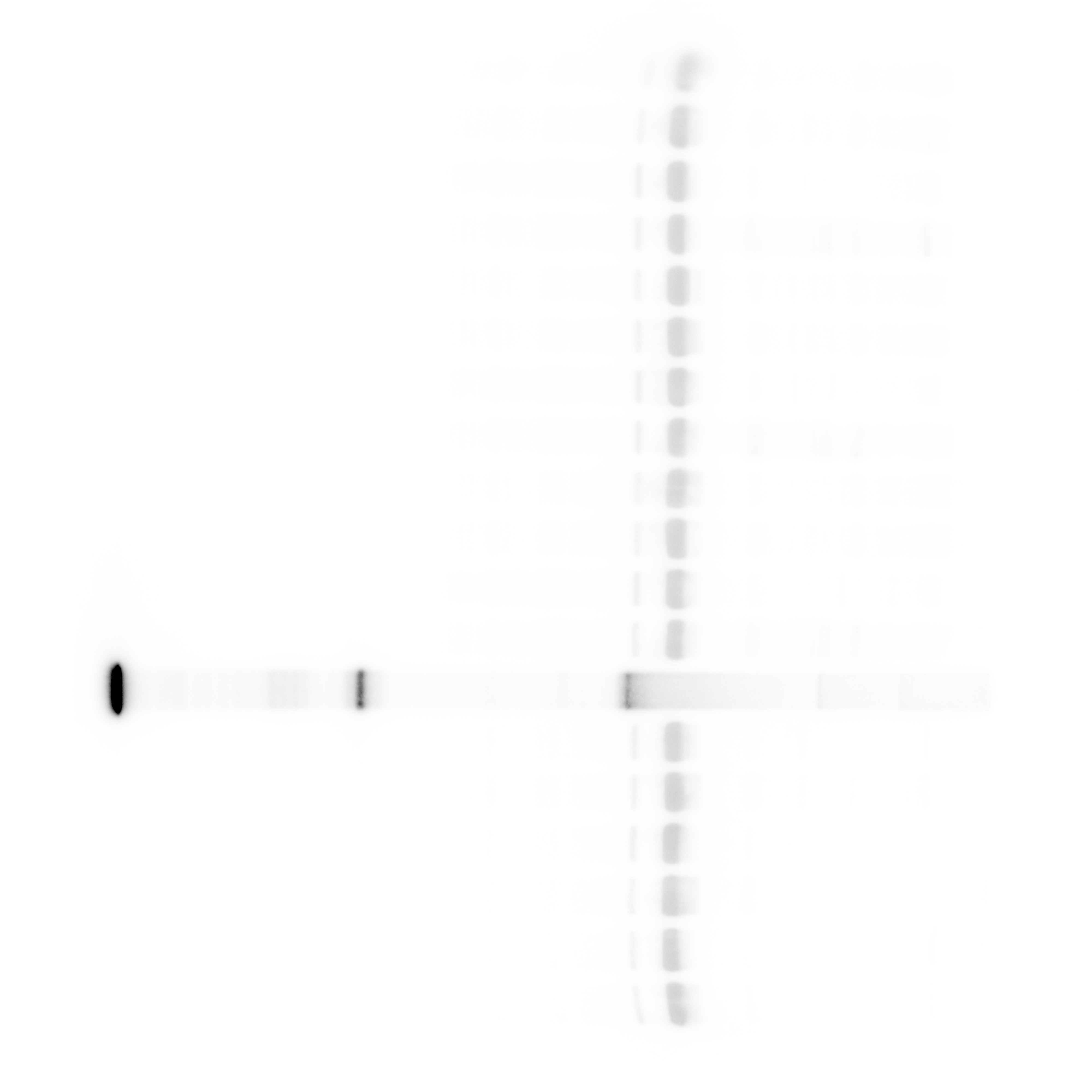

Supplement: Figure 6—source data 2. [file elife-82411-fig6-data2.zip › Fig6A_source/Figure6A_5S.tif]

Figure 6C

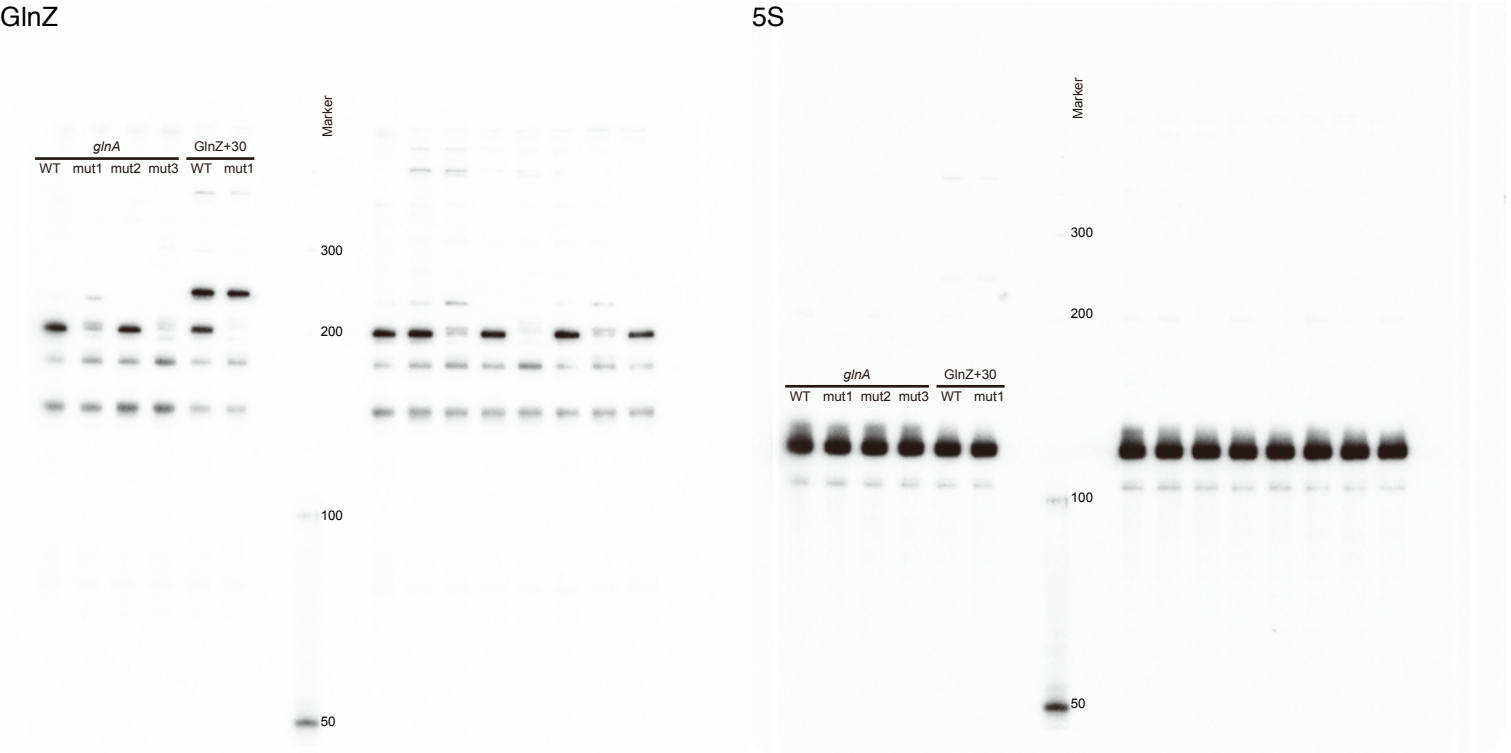

Figure 6D

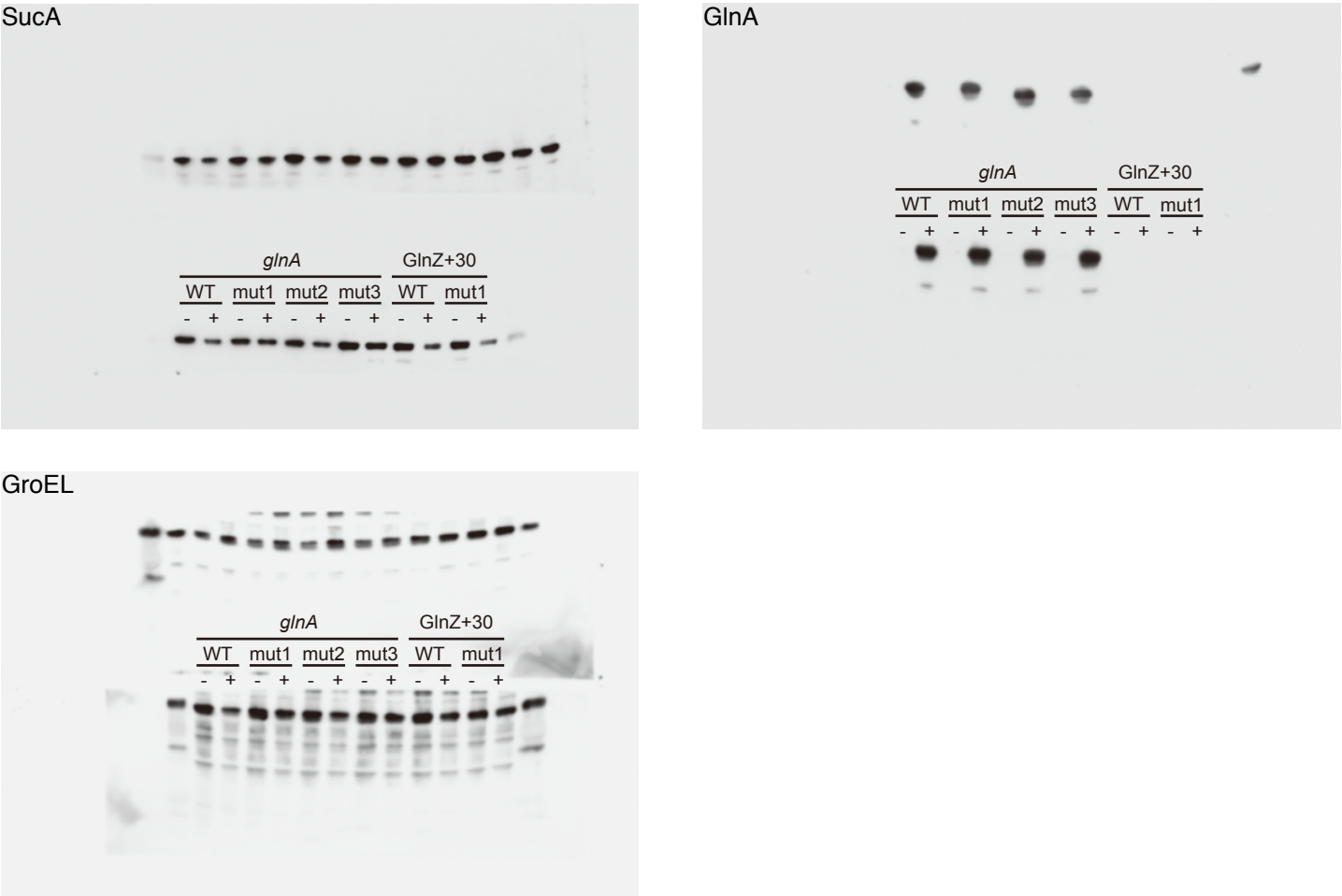

Supplement: Figure 6—source data 3. [file elife-82411-fig6-data3.pdf]

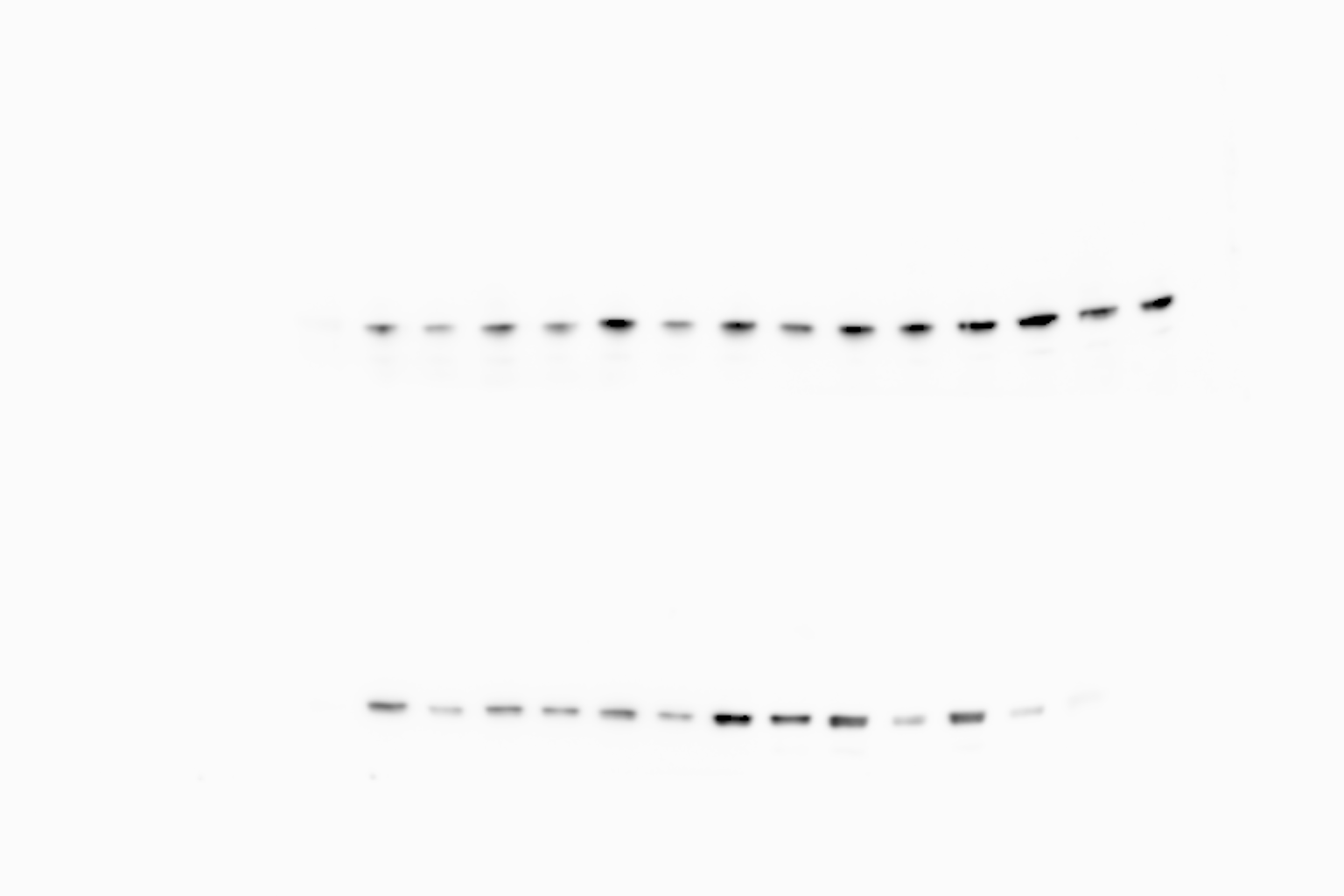

Supplement: Figure 6—source data 4. [file elife-82411-fig6-data4.zip › Fig6_source/Figure6C_SucA.tif]

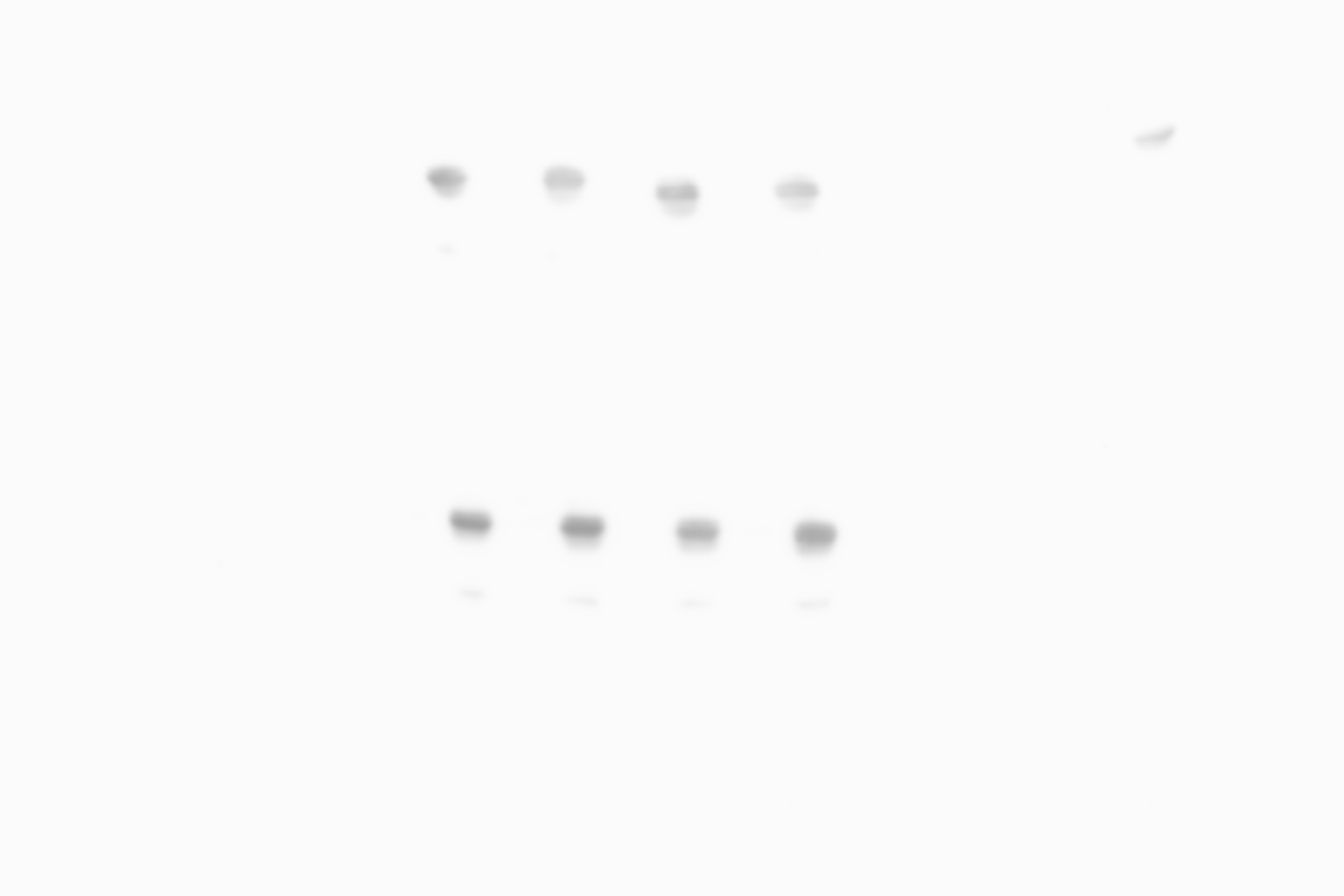

Supplement: Figure 6—source data 4. [file elife-82411-fig6-data4.zip › Fig6_source/Figure6C_GlnA.tif]

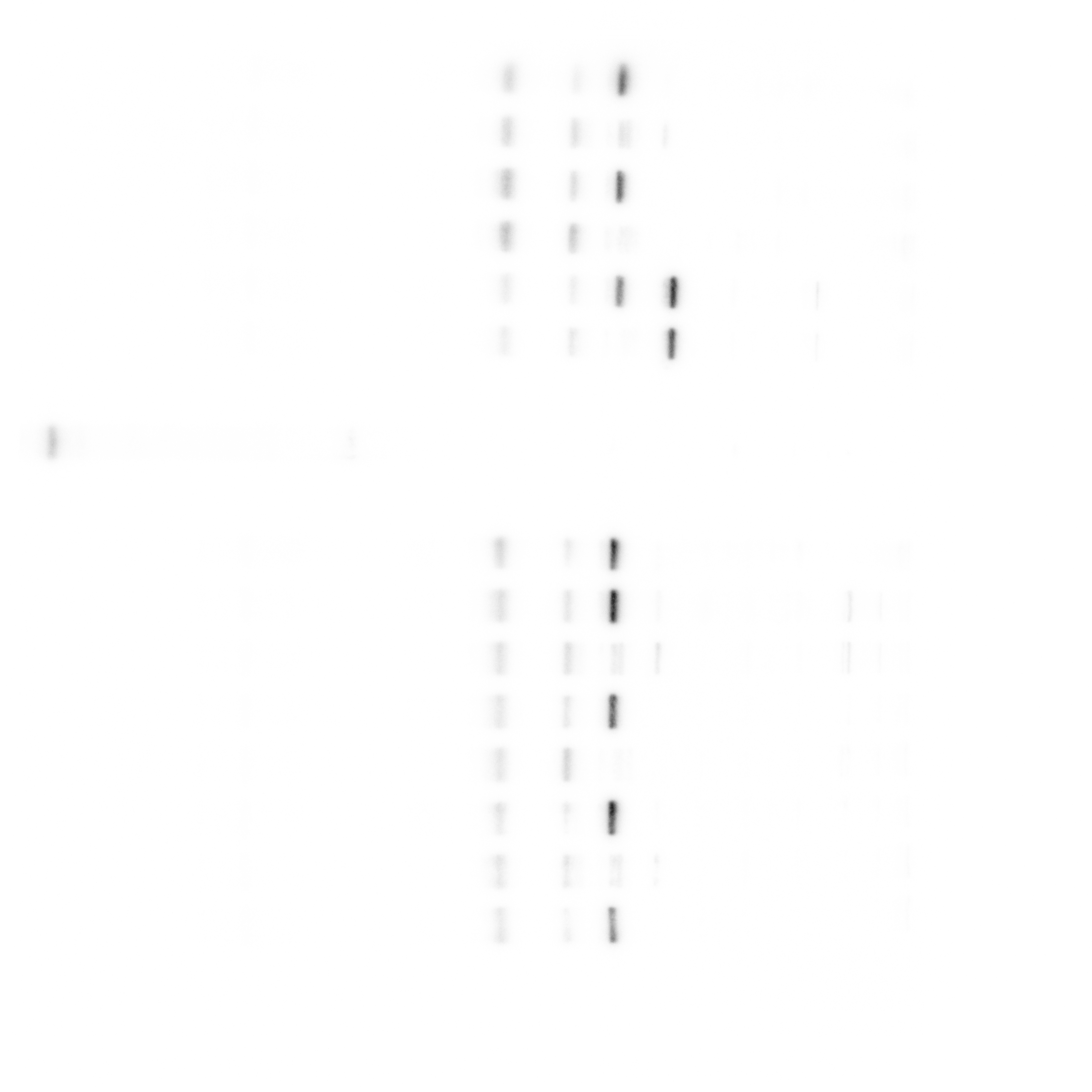

Supplement: Figure 6—source data 4. [file elife-82411-fig6-data4.zip › Fig6_source/Figure6B_GlnZ.tif]

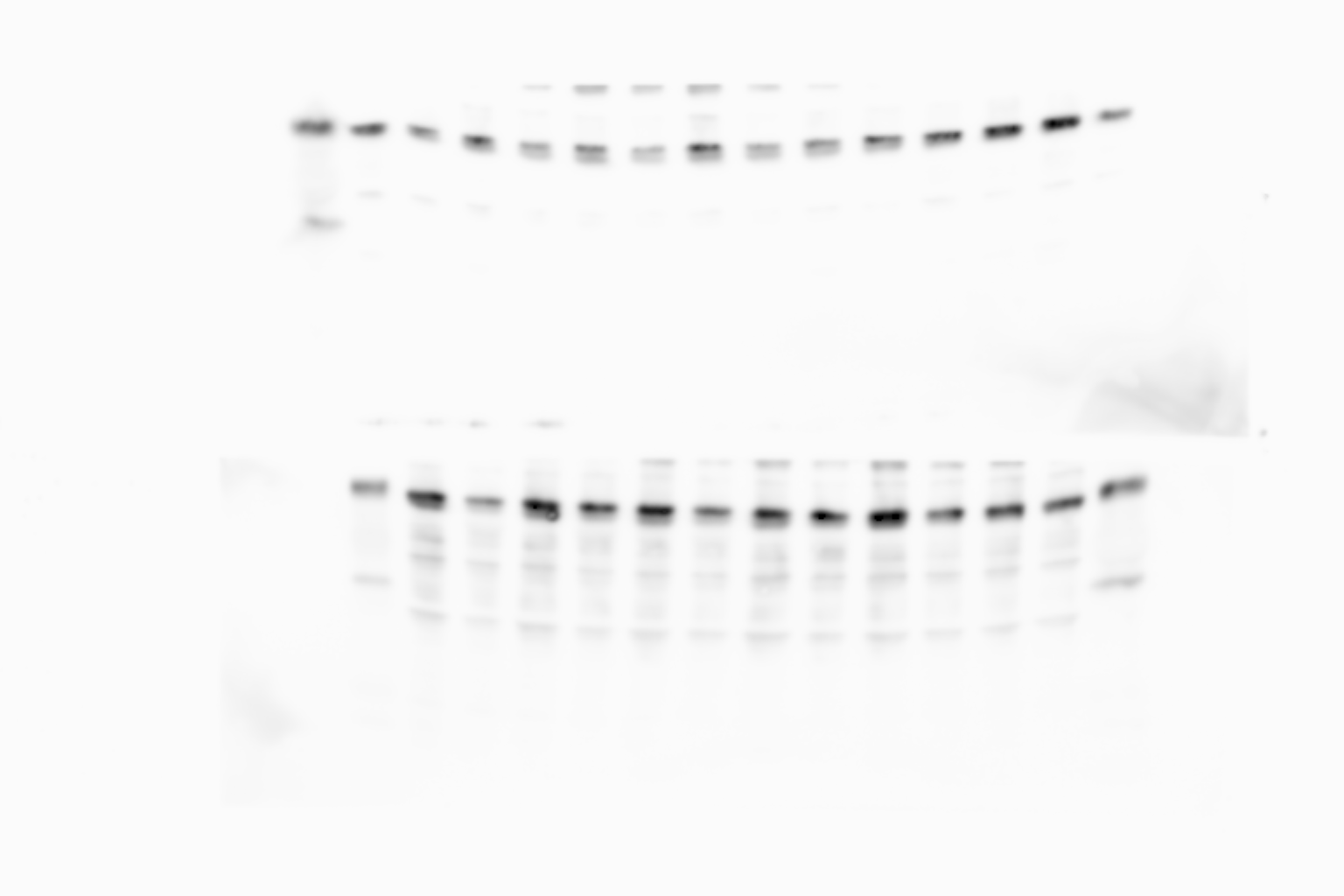

Supplement: Figure 6—source data 4. [file elife-82411-fig6-data4.zip › Fig6_source/Figure6C_GroEL.tif]

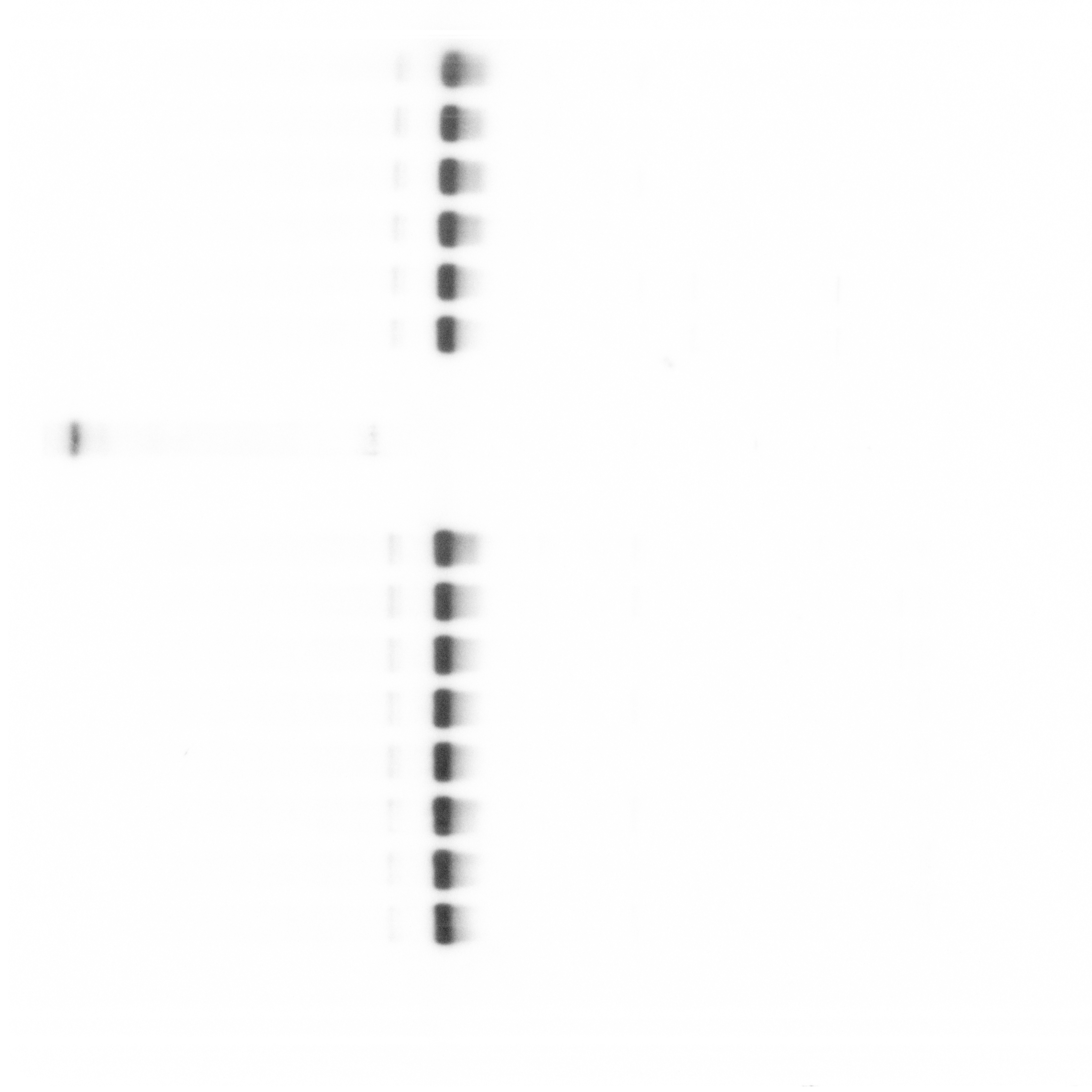

Supplement: Figure 6—source data 4. [file elife-82411-fig6-data4.zip › Fig6_source/Figure6B_5S.tif]
