# Supplementary material for: Glutamine synthetase mRNA releases sRNA from its 3′UTR to regulate carbon/nitrogen metabolic balance in Enterobacteriaceae
Source: eLife. 2022 Nov 28;11:e82411. doi: 10.7554/eLife.82411 (PMC9731577; doi:10.7554/eLife.82411)
Supplement: Supplementary file 1. [file elife-82411-supp1.docx]

**Supplementary File 1.** Genes downregulated upon GlnZ1 overexpression in *S*. Typhimurium SL1344.

| **Name** | **log_2_ fold change** | ***p* value** | **FDR** |
| --- | --- | --- | --- |
| SraB | 3.02 | 3.4E-05 | 3.2E-03 |
| SL1344_4248 | 2.84 | 6.4E-11 | 1.5E-07 |
| *asnA* | 2.71 | 3.6E-09 | 2.8E-06 |
| SL1344_0301 | 2.59 | 1.1E-07 | 4.6E-05 |
| *fabA* | 2.33 | 1.6E-08 | 9.4E-06 |
| *sirC* | 2.31 | 4.6E-06 | 9.1E-04 |
| ***glnP*** | **2.25** | **1.1E-04** | **6.7E-03** |
| ***deoD*** | **2.23** | **1.9E-10** | **3.1E-07** |
| ***glnQ*** | **2.14** | **1.3E-08** | **9.1E-06** |
| *rpsG* | 2.09 | 6.1E-05 | 4.4E-03 |
| *aroQ* | 2.06 | 2.8E-06 | 7.1E-04 |
| *rpsL* | 2.00 | 5.5E-05 | 4.4E-03 |
