# Supplementary material for: Glutamine synthetase mRNA releases sRNA from its 3′UTR to regulate carbon/nitrogen metabolic balance in Enterobacteriaceae
Source: eLife. 2022 Nov 28;11:e82411. doi: 10.7554/eLife.82411 (PMC9731577; doi:10.7554/eLife.82411)
Supplement: Supplementary file 2. [file elife-82411-supp2.docx]

**Supplementary File 2.** Bacterial strains used in this study.

| **Strain** | **Relevant markers/ genotype** | **Reference/ source** |
| --- | --- | --- |
| ***S. enterica* subsp. Typhimurium** | | |
| SL1344 | LT2 *hisG* | Laboratory stock |
| Δ*glnZ*::*kan* | SL1344 Δ*glnA* 3´UTR::*kan* | This study |
| Δ*glnZ* | SL1344 Δ*glnA* 3´UTR::FRT | This study |
| ***E. coli*** | | |
| BW25113 | F^-^ λ^-^ *rrnB3* Δ*lacZ*4787 *hsdR*514 Δ(*araBAD*)567 Δ(*rhaBAD*)568 *rph*-1 | NBPR strain |
| O157 |  | Laboratory stock |
| O111 |  | Laboratory stock |
| Δ*glnZ*::*kan* | BW25113 Δ*glnZ*::*kan* | This study |
| Δ*glnZ*::*cat* I-SceI | BW25113 Δ*glnZ*:: *cat* I-SceI | This study |
| Δ*glnZT*::*kan* | BW25113 Δ*glnZT*::*kan* | This study |
| Δ*glnZ* | BW25113 Δ*glnZ*::FRT | This study |
| Δ*glnZT* | BW25113 Δ*glnZT*::FRT | This study |
| *glnZ*_O157_ | BW25113 *glnZ*_O157_ | This study |
| *glnZ*_O111_ | BW25113 *glnZ*_O111_ | This study |
| *ntrC*::3xFLAG | BW25113 *ntrC*::3xFLAG *kan* | This study |
| Δ*glnZ ntrC*:::3xFLAG | BW25113 Δ*glnZ::* FRT *ntrC*::3xFLAG *kan* | This study |
| ΔΔ*glnZ ntrC*:::3xFLAG | BW25113 ΔΔ*glnZ*::FRT *ntrC*::3xFLAG *kan* | This study |
| *glnZ*_O157_ *ntrC*:::3xFLAG | BW25113 *glnZ*_O157_ *ntrC*::3xFLAG *kan* | This study |
| *glnZ*_O111_ *ntrC*:::3xFLAG | BW25113 *glnZ*_O111_ *ntrC*::3xFLAG *kan* | This study |
| Δ*nac* | BW25113 Δ*nac::kan* (JW1967) | NBPR strain |
| Δ*glnZ* Δ*nac* | BW25113 Δ*glnZ::*FRT Δ*nac::kan* | This study |
| TM587 | W3110 *mlc* Δ*hfq*::*cat* | (Morita *et al.*, 2005) |
| TM151 | W3110 *mlc* *ams-1*::Tn*10* | (Morita *et al.*, 2006) |
| ST201 | W3110 *rnc14*::Tn*10* | (Sunohara *et al.*, 2004) |
| Δ*glnZ* Δ*hfq* | BW25113 Δ*glnZ::*FRT Δ*hfq*::*cat* | This study |
| Δ*glnZ ams-1* | BW25113 Δ*glnZ::*FRT *ams-1*::Tn*10* | This study |
| *rnc14* | BW25113 *rnc14*::Tn*10* | This study |
| Δ*glnZ rnc14* | BW25113 Δ*glnZ::*FRT *rnc14*::Tn*10* | This study |
