# Supplementary material for: Glutamine synthetase mRNA releases sRNA from its 3′UTR to regulate carbon/nitrogen metabolic balance in Enterobacteriaceae
Source: eLife. 2022 Nov 28;11:e82411. doi: 10.7554/eLife.82411 (PMC9731577; doi:10.7554/eLife.82411)
Supplement: Supplementary file 3. [file elife-82411-supp3.docx]

**Supplementary File 3.** Plasmids used in this study.

| **Name** | **Relevant fragment** | **Comment** | **Origin / marker** | **Reference** |
| --- | --- | --- | --- | --- |
| pJV300 | control plasmid | Constitutive expression plasmid | ColE1/ Amp^R^ | (Urban and Vogel, 2007) |
| pP_L_*-*GlnZ1 | P_LlacO_*-glnZ1* | *Salmonella glnZ1* expression plasmid | ColE1/ Amp^R^ | this study |
| pP_L_*-*GlnZ1 G149C | P_LlacO_ *-glnZ1* G149C | *Salmonella glnZ1* G149C mutant | ColE1/ Amp^R^ | this study |
| pP_L_*-*GlnZ1 G146C | P_LlacO_*-glnZ1* G146C | *Salmonella glnZ1* G146C mutant | ColE1/ Amp^R^ | this study |
| pP_L_*-*GlnZ1 G141U | P_LlacO_*-glnZ1* G141U | *Salmonella glnZ1* G141U mutant | ColE1/ Amp^R^ | this study |
| pP_L_*-*GlnZ1 G141U/G146C | P_LlacO_*-glnZ1* G141U/G146C | *Salmonella glnZ1* G141U/G146C mutant | ColE1/ Amp^R^ | this study |
| pP_L_*-*GlnZ2 | P_LlacO_*-glnZ2* | *Salmonella glnZ2* expression plasmid | ColE1/ Amp^R^ | this study |
| pP_L_*-*GlnZ_K12_ | P_LlacO_*- glnZ* _K12_ | *E. coli glnZ* _K12_ expression plasmid | ColE1/ Amp^R^ | this study |
| pP_L_*-*GlnZ_K12_ G15C | P_LlacO_*- glnZ* _K12_ G15C | *E. coli glnZ* _K12_ G15C mutant | ColE1/ Amp^R^ | this study |
| pP_L_*-*GlnZ_K12_ G12C | P_LlacO_*- glnZ* _K12_ G12C | *E. coli glnZ* _K12_ G12C mutant | ColE1/ Amp^R^ | this study |
| pP_L_*-*GlnZ_O157_ | P_LlacO_*-glnZ* _O157_ | *E. coli glnZ* _O157_ expression plasmid | ColE1/ Amp^R^ | this study |
| pP_L_*-*GlnZ_O157_ G49C | P_LlacO_*-glnZ* _O157_ G49C | *E. coli glnZ* _O157_ G49C mutant | ColE1/ Amp^R^ | this study |
| pP_L_*-*GlnZ_O157_ G46C | P_LlacO_*- glnZ* _O157_ G46C | *E. coli glnZ* _O157_ G46C mutant | ColE1/ Amp^R^ | this study |
| pP_L_*-*GlnZ_O157_ U41G | P_LlacO_*- glnZ* _O157_ U41G | *E. coli glnZ* _O157_ U41G mutant | ColE1/ Amp^R^ | this study |
| pP_L_*-*GlnZ_O111_ | P_LlacO_*-glnZ* _O111_ | *E. coli glnZ* _O111_ expression plasmid | ColE1/ Amp^R^ | this study |
| pKP8-35 | control plasmid | Arabinose-inducible pBAD plasmid | pBR322/ Amp^R^ | (Papenfort *et al.*, 2006) |
| pBAD-GlnZ1 | P_araBAD_*-glnZ1* | *Salmonella glnZ1* expression plasmid | pBR322/ Amp^R^ | this study |
| pBAD-*glnA* *sal* | P_araBAD_*-glnA* *sal* | *Salmonella glnA* expression plasmid | pBR322/ Amp^R^ | this study |
| pBAD-GlnZ+90 *sal* | P_araBAD_*-* preGlnZ *sal* | *Salmonella* premature *glnZ* expression plasmid | pBR322/ Amp^R^ | this study |
| pBAD-*glnA* *eco* | P_araBAD_*-glnA* *eco* | *E. coli glnA* _K12_ expression plasmid | pBR322/ Amp^R^ | this study |
| pBAD-*glnA* mut1 | P_araBAD_*-glnA* *eco* mut1 | *E. coli glnA* _K12_ mutant in site 1 | pBR322/ Amp^R^ | this study |
| pBAD-*glnA* mut2 | P_araBAD_*-glnA* *eco* mut2 | *E. coli glnA* _K12_ mutant in site 2 | pBR322/ Amp^R^ | this study |
| pBAD-*glnA* mut3 | P_araBAD_*-glnA* *eco* mut3 | *E. coli glnA* _K12_ mutant in site 1 and 2 | pBR322/ Amp^R^ | this study |
| pBAD-GlnZ+90 *eco* | P_araBAD_*-* preGlnZ *eco* | *E. coli* premature *glnZ* (90-nt extension) expression plasmid | pBR322/ Amp^R^ | this study |
| pBAD-GlnZ+30 *eco* | P_araBAD_*-* GlnZ_227_ *eco* | *E. coli* premature *glnZ* (30-nt extension) expression plasmid | pBR322/ Amp^R^ | this study |
| pBAD-GlnZ+30 *eco* mut1 | P_araBAD_*-* GlnZ_227_ *eco* mut1 | *E. coli* premature *glnZ* (30-nt extension) mutant in site 1 | pBR322/ Amp^R^ | this study |
| pXG-30sf | P_LtetO_-FLAG::*glmU*-*glmS::gfp* | Translational sfGFP fusion plasmid for dicistronic targets | pSC101* / Cm^R^ | (Corcoran *et al.*, 2012) |
| pXG-30sf-*sucA sal* | P_LtetO_-FLAG::*sdhB*-*sucA::gfp* | *S. enterica* *sdhB*-*sucA* translational fusion plasmid | pSC101*/ Cm^R^ | this study |
| pXG-30sf-*sucA eco* | P_LtetO_-FLAG::*sdhB*-*sucA::gfp* | *E. coli* *sdhB*-*sucA* translational fusion plasmid | pSC101*/ Cm^R^ | this study |
| pXG-30sf-*deoD sal* | P_LtetO_-FLAG::*deoB*-*deoD::gfp* | *S. enterica deoBD* translational GFP fusion plasmid | pSC101*/ Cm^R^ | this study |
| pXG-30sf-*glnP sal* | P_LtetO_-FLAG::*glnH*-*glnP::gfp* | *S. enterica glnHP* translational GFP fusion plasmid | pSC101*/ Cm^R^ | this study |
| pXG-30sf-*aceE sal* | P_LtetO_-FLAG::*pdhR*-*aceE::gfp* | *S. enterica* *pdhR*-*aceE* translational fusion plasmid | pSC101*/ Cm^R^ | this study |
| pXG-30sf-*aceE eco* | P_LtetO_-FLAG::*pdhR*-*aceE::gfp* | *E. coli* *pdhR*-*aceE* translational fusion plasmid | pSC101*/ Cm^R^ | this study |
