# Supplementary material for: Glutamine synthetase mRNA releases sRNA from its 3′UTR to regulate carbon/nitrogen metabolic balance in Enterobacteriaceae
Source: eLife. 2022 Nov 28;11:e82411. doi: 10.7554/eLife.82411 (PMC9731577; doi:10.7554/eLife.82411)
Supplement: Supplementary file 4. [file elife-82411-supp4.docx]

**Supplementary File 4.** DNA oligonucleotides used in this study.

| **Name** | **Sequence (5´ -> 3´ direction)** | **Used for** |
| --- | --- | --- |
| **Northern blot** | | |
| MMO-0416 | aaagtttccacggcaact | Probe for GlnZ |
| MMO-0417 | atcctgggatgggctgaaag | Probe for GlnZ2 |
| MMO-0418 | aactcctgacgcctttcacg | Probe for GlnZ1 |
| MMO-0419 | atgcagagatgggctacaga | Probe for GlnZ K12 |
| MMO-1056 | ACTACCATCGGCGCTACGGC | Probe for 5S rRNA |
| JVO-2907 | GAAGATTGTTGCCCGGCGATTTG | Probe for SroC sal |
| JVO-5622 | GAAGATTGTTACCCAGCGTATTG | Probe for SroC eco |
| SP6-AS *glnA*sal | ATTGTCGTTAGAACGCGGCTACAATTAATACATAACCTTATGTATCATACACATACGATTTAGGTGACACTATAGacgcacgcggtcatcttcttcgcgacgcagcgcaatatacgcatcgatcgcttcatcagtgaacacgccgcctgctttcaggaactcgcggtccaggtccagcgcgttcagcgcttcttccagagaacccgctacctgtgggatctctttcgcttcttccggcggcaggtcatacaggtttttgtccatggcttcgcccg | Probe for *glnA* sal |
| SP6-AS *glnA*eco | ATTGTCGTTAGAACGCGGCTACAATTAATACATAACCTTATGTATCATACACATACGATTTAGGTGACACTATAGacgcacgcggtcatcttcttcgcgacgcagagcgatgtacgcatcaattgcttcgtcagtgaacacgccaccggctttcaggaactcgcggtccagatccagttcgttcagtgcttcttccagagagcctgcaacctgtgggatctctttcgcttcttctggcggcaggtcatacaggtttttgtccatggcttcgcccg | Probe for *glnA* eco |
| ***glnA* and GlnZ cloning** | | |
| MMO-0354 | gtttttTCTagattgttggtggagaaaaaag | *glnA*/GlnZ sal |
| MMO-0355 | atcgtatattaaaaatccgacaaatttc | GlnZ1 sal |
| MMO-0356 | agtttttgagttgccgtgga | GlnZ2 sal |
| MMO-0384 | acggcgacacggccag | *glnA* sal |
| MMO-0386 | GTAGAGTTTGAGCTGTACTACAGC | GlnZ+30 |
| MMO-0399 | acggcgacacggccaaaataattg | *glnA* eco |
| MMO-0405 | Agtgttttagttgccgtgg | GlnZ K12 |
| MMO-0406 | gtttttTCTagaattgacggagaaaaaag | *glnA* /GlnZ eco |
| MMO-0786 | gatgcgtacatcgctctg | GlnZ+90 |
| MMO-0787 | CATaaagcagtctcctgaaca | *glnL* eco from ATG |
| MMO-0788 | atagttgaagttgtactaccc | GlnZ O157/O111 |
| ***glnA* and GlnZ mutagenesis** | | |
| MMO-0361 | gttgccCtggaaactttcagcccat | GlnZ sal G149C |
| MMO-0362 | tttccaGggcaactcaaaaactcctg | GlnZ sal G149C |
| MMO-0694 | tgagttCcgtggaaactttcagcc | GlnZ sal G146C |
| MMO-0695 | ccacggGaactcaaaaactcctgacgc | GlnZ sal G146C |
| MMO-0857 | gttttTagttgccgtggaaactttc | GlnZ sal G141U |
| MMO-0858 | gcaactAaaaactcctgacgcctttc | GlnZ sal G141U |
| MMO-0859 | gttttTagttCccgtggaaactttc | GlnZ sal G141U/G146C |
| MMO-0860 | gGaactTaaaactcctgacgcctttc | GlnZ sal G141U/G146C |
| MMO-0411 | gttgccCtggaaacttttcgcctgt | GlnZ K12 G15C |
| MMO-0412 | tttccaGggcaactaaaacacTgtgctc | GlnZ K12 G15C |
| MMO-0718 | ttagttCccgtggaaacttttcgcc | GlnZ K12 G12C |
| MMO-0719 | ccacggGaactaaaacactGTGCTCAGT | GlnZ K12 G12C |
| MMO-1168 | gggattGagttgccgtggaaactttc | GlnZ O157 U41G |
| MMO-1169 | gcaactCaatcccggcgttgttgc | GlnZ O157 U41G |
| MMO-1180 | gttgccCtggaaactttcagcccatc | GlnZ O157 G49C |
| MMO-1181 | tttccaGggcaactaaatcccggc | GlnZ O157 G49C |
| MMO-1182 | ttagttCccgtggaaactttcagcc | GlnZ O157 G46C |
| MMO-1183 | ccacggGaactaaatcccggcgttg | GlnZ O157 G46C |
| MMO-0617 | gtagagCGGgagctgtactacagcgtcta | *glnA* K12 RNase E mut2 |
| MMO-0618 | cagctcCCGctctaccggatgcggag | *glnA* K12 RNase E mut2 |
| MMO-0621 | aagtgtGGtagttgccgtggaaacttttc | *glnA* K12 RNase E mut1 |
| MMO-0622 | caactaCCacacttagacgctgtagtacag | *glnA* K12 RNase E mut1 |
| **GlnZ target cloning** | | |
| MMO-0325 | gtttttATGCATgcattcagcgtattccg | *sdhB-sucA* GFP fusion |
| MMO-0326 | gtttttGCTAGCCAACCAGGCTTTCAAAGC | *sdhB-sucA* GFP fusion |
| MMO-0529 | gtttttATGCATccgatggactacggtaaaa | *deoBDsal* GFP fusion |
| MMO-0530 | gtttttGCTAGCcggcatcaatacgacgt | *deoBDsal* GFP fusion |
| MMO-0594 | gtttttATGCATAACGAAATCTACAAAAAATGGT | *glnHP* GFP fusion |
| MMO-0595 | gtttttgctagcccagtcaaactgcatatgt | *glnHP* GFP fusion |
| MMO-0701 | gtttttATGCATgagatgctgccgctggtg | *pdhR-aceE* GFP fusion |
| MMO-0702 | gtttttGCTAGCaacaccttcttcacggatgacc | *pdhR-aceE* GFP fusion |
| **GlnZ target mutagenesis** | | |
| MMO-0363 | tatccaGggcgaaatactcgtcatag | *sucAsal* C-166G |
| MMO-0364 | ttcgccCtggatactaaccacgcatac | *sucAsal* C-166G |
| MMO-0369 | tatccaGggcgaagtaagcataaaaaag | *sucAeco* C-38G |
| MMO-0370 | ttcgccCtggatactaccacgcacag | *sucAeco* C-38G |
| MMO-0691 | acaccaGggtaacaggaacgacatatg | *glnPsal* C-18G |
| MMO-0692 | gttaccCtggtgtaaatagtcaaatgct | *glnPsal* C-18G |
| MMO-0697 | agatggGaactccacatattaatgcagaaatg | *deoDsal* C5G |
| MMO-0698 | ggagttCccatcttcagttccctttaaatttg | *deoDsal* C5G |
| MMO-0720 | gcgcggGaactaaacgcagaacctgtcttattaagc | *aceEsal* C-99G |
| MMO-0721 | ttagttCccgcgcacatttttgcgc | *aceEsal* C-99G |
| MMO-0722 | gcgcggGaactaaacgtagaacctgtcttattg | *aceEeco* C-99G |
| MMO-0723 | ttagttCccgcgcttttatatgcgc | *aceEeco* C-99G |
| MMO-1431 | atgcttaagggatcacgatg | *sucA* deletion |
| MMO-1432 | ttcgccgtggatactacc | *sucAeco* deletion |
| MMO-1433 | ttcgccgtggatactaacc | *sucAsal* deletion |
| MMO-1434 | aatactctaaataggtcacgtttc | *sucAsal* deletion |
| MMO-1437 | aaataagcagaaaagatgcttaagg | *sucAsal* deletion |
| **Lambda Red recombination** | | |
| MMO-0371 | CCCGCACCCGGTAGAGTTTGAGCTGTACTACAGCGTTTAAGTGTAGGCTGGAGCTGCTTC | Sense oligo to construct *Salmonella* Δ*glnZ*::*kan* |
| MMO-0372 | agattgttggtggagaaaaaagcccatcctgggatgggctggtccatatgaatatcctccttag | Antisense oligo to construct *Salmonella* Δ*glnZ*::*kan* |
| MMO-0401 | TCCGCATCCGGTAGAGTTTGAGCTGTACTACAGCGTCTAAGTGTAGGCTGGAGCTGCTTC | Sense oligo to construct *E. coli* Δ*glnZ*::*kan* |
| MMO-0402 | agagaattgacggagaaaaaagcccatgcagagatgggctggtccatatgaatatcctccttag | Antisense oligo to construct *E. coli* Δ*glnZ*::*kan* |
| MMO-0451 | agagaattgacggagaaaaaagcccatgcagagatgggctCTAGACTATATTACCCTGTT | Antisense oligo to construct *E. coli* Δ*glnZ*::*cat* I-SceI |
| MMO-0583 | ttacggataaaaagcgcgaagcatcagagaattgacggagggtccatatgaatatcctccttag | Antisense oligo to construct *E. coli* Δ*glnZT* ::*kan* |
| MMO-0757 | caacaccctgacgcgtaagttaaaagagctggggatggagGACTACAAAGACCATGACGG | *ntrC*::3xFLAG insertion with pSUB13 |
| MMO-0758 | caatttgcgctcaataatcaatctttacacacaagctgtgTCCATATGAATATCCTCCTTAG | *ntrC*::3xFLAG insertion with pSUB13 |
