# Supplementary material for: Glutamine synthetase mRNA releases sRNA from its 3′UTR to regulate carbon/nitrogen metabolic balance in Enterobacteriaceae
Source: eLife. 2022 Nov 28;11:e82411. doi: 10.7554/eLife.82411 (PMC9731577; doi:10.7554/eLife.82411)
Supplement: Supplementary file 5. [file elife-82411-supp5.docx]

**Supplementary File 5.** Inserts of GlnZ mutant plasmids.

The modified nucleotides are highlighted in magenta. XbaI sites introduced for cloning were highlighted in yellow.

| **Plasmid** | **Insert from +1 to end of *gcvB* terminator** | **Positions deleted or mutated** |
| --- | --- | --- |
| pP_L_*-*GlnZ1 | atcgtatattaaaaatccgacaaatttcgcgttgctgcaaggcagcaactgagcacatccccaggagcatagatagcgatgtgactggggtaagcgaaggcagccaacgcagcagcagcgtgaaaggcgtcaggagtttttgagttgccgtggaaactttcagcccatcccaggatgggcttttttctccaccaacaatctaga |  |
| pP_L_*-*GlnZ1 G149C | atcgtatattaaaaatccgacaaatttcgcgttgctgcaaggcagcaactgagcacatccccaggagcatagatagcgatgtgactggggtaagcgaaggcagccaacgcagcagcagcgtgaaaggcgtcaggagtttttgagttgccCtggaaactttcagcccatcccaggatgggcttttttctccaccaacaatctaga | G149C |
| pP_L_*-*GlnZ1 G146C | atcgtatattaaaaatccgacaaatttcgcgttgctgcaaggcagcaactgagcacatccccaggagcatagatagcgatgtgactggggtaagcgaaggcagccaacgcagcagcagcgtgaaaggcgtcaggagtttttgagttCccgtggaaactttcagcccatcccaggatgggcttttttctccaccaacaatctaga | G146C |
| pP_L_*-*GlnZ1 G141U | atcgtatattaaaaatccgacaaatttcgcgttgctgcaaggcagcaactgagcacatccccaggagcatagatagcgatgtgactggggtaagcgaaggcagccaacgcagcagcagcgtgaaaggcgtcaggagtttttagttgccgtggaaactttcagcccatcccaggatgggcttttttctccaccaacaatctaga | G141U |
| pP_L_*-*GlnZ1 G141U/G146C | atcgtatattaaaaatccgacaaatttcgcgttgctgcaaggcagcaactgagcacatccccaggagcatagatagcgatgtgactggggtaagcgaaggcagccaacgcagcagcagcgtgaaaggcgtcaggagtttttagttCccgtggaaactttcagcccatcccaggatgggcttttttctccaccaacaatctaga | G141U/G146C |
| pP_L_*-*GlnZ2 | agtttttgagttgccgtggaaactttcagcccatcccaggatgggcttttttctccaccaacaatctaga |  |
| pP_L_*-*GlnZ_K12_ | agtgttttagttgccgtggaaacttttcgcctgtctctggcaggcctgggatcggtggcaagcacatcacgccggatgcgacgcaaatgcgtcttatccggcctacacggtgatgatgtggtaggccggagcaggtgagtcgctctccaacgtgaagtttgtcagctatctgtagcccatctctgcatgggcttttttctccgtcaattctaga |  |
| pP_L_*-*GlnZ_K12_ G15C | agtgttttagttgccCtggaaacttttcgcctgtctctggcaggcctgggatcggtggcaagcacatcacgccggatgcgacgcaaatgcgtcttatccggcctacacggtgatgatgtggtaggccggagcaggtgagtcgctctccaacgtgaagtttgtcagctatctgtagcccatctctgcatgggcttttttctccgtcaattctaga | G15C |
| pP_L_*-*GlnZ_K12_ G12C | agtgttttagttCccgtggaaacttttcgcctgtctctggcaggcctgggatcggtggcaagcacatcacgccggatgcgacgcaaatgcgtcttatccggcctacacggtgatgatgtggtaggccggagcaggtgagtcgctctccaacgtgaagtttgtcagctatctgtagcccatctctgcatgggcttttttctccgtcaattctaga | G12C |
| pP_L_*-*GlnZ_O157_ | atagttgaagttgtactacccggcgcaacaacgccgggatttagttgccgtggaaactttcagcccatctctgcatgggcttttttctccgtcaattctaga |  |
| pP_L_*-*GlnZ_O157_ G49C | atagttgaagttgtactacccggcgcaacaacgccgggatttagttgccCtggaaactttcagcccatctctgcatgggcttttttctccgtcaattctaga | G49C |
| pP_L_*-*GlnZ_O157_ G46C | atagttgaagttgtactacccggcgcaacaacgccgggatttagttCccgtggaaactttcagcccatctctgcatgggcttttttctccgtcaattctaga | G46C |
| pP_L_*-*GlnZ_O157_ U41G | atagttgaagttgtactacccggcgcaacaacgccgggattgagttgccgtggaaactttcagcccatctctgcatgggcttttttctccgtcaattctaga | U41G |
| pP_L_*-*GlnZ_O111_ | atagttgaagttgtactacccggcgcaacaacgccggaggttcaaaccaggcccatctggcgtaattgttgcagccagtttgaacacggacagcgcgcagaacccggagcgtacactggtacgtgaggagttcgagcactgcccaggttcaaaatggcaaataaaatagcctgatgggactggtttttagttgccgtggaaatttcagcccatctctgcatgggcttttttctccgtcaattctaga |  |
