# Supplementary material for: Glutamine synthetase mRNA releases sRNA from its 3′UTR to regulate carbon/nitrogen metabolic balance in Enterobacteriaceae
Source: eLife. 2022 Nov 28;11:e82411. doi: 10.7554/eLife.82411 (PMC9731577; doi:10.7554/eLife.82411)
Supplement: Supplementary file 6. [file elife-82411-supp6.docx]

**Supplementary File 6.** Details of GFP fusion plasmids.

| **Target gene** | **Vector** | **Oligonucleotide** | **Upstream ORF [bp]** | **Intergenic region [bp]** | **Downstream ORF [bp]** | **Insert length [bp]** | **Translational fusion to N-terminal FLAG [aa]** | **Translational fusion to C-terminal GFP [aa]** |
| --- | --- | --- | --- | --- | --- | --- | --- | --- |
| *sdhC-sucAsal* | pXG-30sf | MMO-0325 x MMO-0326 | 120 | 523 | 30 | 673 | 40 | 10 |
| *sdhC-sucAeco* | pXG-30sf | MMO-0325 x MMO-0326 | 120 | 300 | 30 | 450 | 40 | 10 |
| *deoBDsal* | pXG-30sf | MMO-0529 x MMO-0530 | 30 | 209 | 60 | 299 | 10 | 20 |
| *glnHPsal* | pXG-30sf | MMO-0594 x MMO-0595 | 42 | 143 | 15 | 200 | 14 | 5 |
| *pdhR-aceEsal* | pXG-30sf | MMO-0701 x MMO-0702 | 189 | 159 | 90 | 438 | 63 | 30 |
| *pdhR-aceEeco* | pXG-30sf | MMO-0701 x MMO-0702 | 189 | 160 | 90 | 439 | 63 | 30 |
