# Supplementary material for: Glutamine synthetase mRNA releases sRNA from its 3′UTR to regulate carbon/nitrogen metabolic balance in Enterobacteriaceae
Source: eLife. 2022 Nov 28;11:e82411. doi: 10.7554/eLife.82411 (PMC9731577; doi:10.7554/eLife.82411)
Supplement: Supplementary file 7. [file elife-82411-supp7.docx]

**Supplementary File 7.** Inserts of GFP fusion plasmids.

Nucleotide sequences of upstream ORF fused with FLAG, intergenic region, and downstream ORF fused with GFP are indicated in blue, black, and red, respectively. The mutated nucleotides in the GlnZ target region are highlighted in magenta. The deleted nucleotides in *sucA* reporter constructs are highlighted in gray. NsiI and NheI sites used for cloning are highlighted in bold in cyan and green, respectively.

| **GFP fusion** | **Insert** |
| --- | --- |
| *sdhC-sucAsal* | ATGCATgcattcagcgtattccgctgtcacagcattatgaactgcgtcagtgtatgtcctaagggactgaacccgacgcgcgctatcggccatattaagtcgatgctgttgcagcgtagcgcataagtagttgtagtggttgccggatggcgacgtacagtcttatccggcctacaggcgcaaccccgtaggcctgataagcgcagcgccatcaggcaatagaagtgcaggaaacctctaaaaactgccatatgattagtaataatcgagatgttcagagcgagacaaggcgcggtctgaacgaatcttcgggagcatagttaactatgtgaccggagtgagcgaaggccgcaacgcagtcgtagcctgaacagcgaagattattagacagtttttaaaggttccttagcggacgcagatgacacgacagtccgcaacaagtgaaccccggcacgcatacagcgtatgcgtggttagtatccacggcgaaatactcgtcatagttcacgttgcatgtgcgttggctgcacttgctcaccccggtcacttactggatgtaagctcccagggattcgcaagcttgccgccttcctgaaacgtgacctatttagagtattaaataagcagaaaagatgcttaagggatcacgatgcagaacagcgctttgaaagcctggttggctagc |
| *sdhC-sucAeco* | ATGCATgcattcagcgtattccgctgtcacagcatcatgaactgcgtcagtgtatgtccgaaggggctgaacccgacgcgcgccatcggccatatcaagtcgatgttgttgcaacgtaatgcgtaaaccgtaggcctgataagacgcgcaagcgtcgcatcaggcaaccagtgccggatgcggcgtgaacgccttatccggcctacaagtcattacccgtaggcctgataagcgcagcgcatcaggcgtaacaaagaaatgcaggaaatctttaaaaactgcccctgacactaagacagtttttaaaggttccttcgcgagccactacgtagacaagagctcgcaagtgaaccccggcacgcacatcactgtgcgtggtagtatccacggcgaagtaagcataaaaaagatgcttaagggatcacgatgcagaacagcgctttgaaagcctggttggctagc |
| *deoBDsal* | ATGCATccgatggactacggtaaaaacatgctgtgatacctgttgtctttcgcgctgtagatgcgttgacttcattcgctcaccccggtcacatagttaatctatgctcccggggattcacgaattcgtcgccttcctacaacgcgaaatacttagggtatttcactgttttgacacacgttttgtaggcctgataaggcgtagccgccatcaggcaataacaacaaatttaaagggaactgaagatggcaactccacatattaatgcagaaatgggtgatttcgctgacgtcgtattgatgccg  gctagc |
| *glnHPsal* | ATGCATaacgaaatctacaaaaaatggttcggtacagaacctaaataaaacgacctgatgttggcttgtggcccggtagtgaggacgctcaccgggcctacagctctttccgtgacggataagacactttccgccatttccgcgccggatagagcatttgactatttacaccacggtaacaggaacgacatatgcagtttgactgggctagc |
| *pdhR-aceEsal* | ATGCATgagatgctgccgctggtgagtacgcatcgcacccgtatatttgaagccattatggccggaaaaccagaagaagcgcgtgaagcgtcacaccgacatctggcgtttatcgaagagattatgctggacagaagccgtgaggagagccgtcgtgaacgcgctttacgccgcctggaacagcgcaagaattagttttttctggcagaacgttaaccagaagatgttgtgaataacgcgcaaaaatgtgcgcggcaactaaacgcagaacctgtcttattaagctttctcacgaaagtttaatgggacaggttccagataactcaacgtattagatagataaggaatacccccatgtcagaacgtttccaaaatgacgtggatccgatcgaaactcgcgactggctacaggcgatcgaatcggtcatccgtgaagaaggtgttgctagc |
| *pdhR-aceEeco* | ATGCATgagatgctgccgctggtgagtagtcaccgcacccgcatatttgaagcgattatggccggtaagccggaagaagcgcgcgaagcatcgcatcgccatctggcctttatcgaagaaattttgctcgacagaagtcgtgaagagagccgccgtgagcgttctctgcgtcgtctggagcaacgaaagaattagtgatttttctggtaaaaattatccagaagatgttgtaaatcaagcgcatataaaagcgcggcaactaaacgtagaacctgtcttattgagctttccggcgagagttcaatgggacaggttccagaaaactcaacgttattagatagataaggaataacccatgtcagaacgtttcccaaatgacgtggatccgatcgaaactcgcgactggctccaggcgatcgaatcggtcatccgtgaagaaggtgttgctagc |
| *sdhC-sucAsal* Δ15 | ATGCATgcattcagcgtattccgctgtcacagcattatgaactgcgtcagtgtatgtcctaagggactgaacccgacgcgcgctatcggccatattaagtcgatgctgttgcagcgtagcgcataagtagttgtagtggttgccggatggcgacgtacagtcttatccggcctacaggcgcaaccccgtaggcctgataagcgcagcgccatcaggcaatagaagtgcaggaaacctctaaaaactgccatatgattagtaataatcgagatgttcagagcgagacaaggcgcggtctgaacgaatcttcgggagcatagttaactatgtgaccggagtgagcgaaggccgcaacgcagtcgtagcctgaacagcgaagattattagacagtttttaaaggttccttagcggacgcagatgacacgacagtccgcaacaagtgaaccccggcacgcatacagcgtatgcgtggttagtatccacggcgaaatactcgtcatagttcacgttgcatgtgcgttggctgcacttgctcaccccggtcacttactggatgtaagctcccagggattcgcaagcttgccgccttcctgaaacgtgacctatttagagtattaaataagcagaaaagatgcttaagggatcacgatgcagaacagcgctttgaaagcctggttggctagc |
| *sdhC-sucAsal* Δ127 | ATGCATgcattcagcgtattccgctgtcacagcattatgaactgcgtcagtgtatgtcctaagggactgaacccgacgcgcgctatcggccatattaagtcgatgctgttgcagcgtagcgcataagtagttgtagtggttgccggatggcgacgtacagtcttatccggcctacaggcgcaaccccgtaggcctgataagcgcagcgccatcaggcaatagaagtgcaggaaacctctaaaaactgccatatgattagtaataatcgagatgttcagagcgagacaaggcgcggtctgaacgaatcttcgggagcatagttaactatgtgaccggagtgagcgaaggccgcaacgcagtcgtagcctgaacagcgaagattattagacagtttttaaaggttccttagcggacgcagatgacacgacagtccgcaacaagtgaaccccggcacgcatacagcgtatgcgtggttagtatccacggcgaaatactcgtcatagttcacgttgcatgtgcgttggctgcacttgctcaccccggtcacttactggatgtaagctcccagggattcgcaagcttgccgccttcctgaaacgtgacctatttagagtattaaataagcagaaaagatgcttaagggatcacgatgcagaacagcgctttgaaagcctggttggctagc |
| *sdhC-sucAsal* Δ142 | ATGCATgcattcagcgtattccgctgtcacagcattatgaactgcgtcagtgtatgtcctaagggactgaacccgacgcgcgctatcggccatattaagtcgatgctgttgcagcgtagcgcataagtagttgtagtggttgccggatggcgacgtacagtcttatccggcctacaggcgcaaccccgtaggcctgataagcgcagcgccatcaggcaatagaagtgcaggaaacctctaaaaactgccatatgattagtaataatcgagatgttcagagcgagacaaggcgcggtctgaacgaatcttcgggagcatagttaactatgtgaccggagtgagcgaaggccgcaacgcagtcgtagcctgaacagcgaagattattagacagtttttaaaggttccttagcggacgcagatgacacgacagtccgcaacaagtgaaccccggcacgcatacagcgtatgcgtggttagtatccacggcgaaatactcgtcatagttcacgttgcatgtgcgttggctgcacttgctcaccccggtcacttactggatgtaagctcccagggattcgcaagcttgccgccttcctgaaacgtgacctatttagagtattaaataagcagaaaagatgcttaagggatcacgatgcagaacagcgctttgaaagcctggttggctagc |
| *sdhC-sucAeco* Δ15 | ATGCATgcattcagcgtattccgctgtcacagcatcatgaactgcgtcagtgtatgtccgaaggggctgaacccgacgcgcgccatcggccatatcaagtcgatgttgttgcaacgtaatgcgtaaaccgtaggcctgataagacgcgcaagcgtcgcatcaggcaaccagtgccggatgcggcgtgaacgccttatccggcctacaagtcattacccgtaggcctgataagcgcagcgcatcaggcgtaacaaagaaatgcaggaaatctttaaaaactgcccctgacactaagacagtttttaaaggttccttcgcgagccactacgtagacaagagctcgcaagtgaaccccggcacgcacatcactgtgcgtggtagtatccacggcgaagtaagcataaaaaagatgcttaagggatcacgatgcagaacagcgctttgaaagcctggttggctagc |
